# GLP-1 Receptor Agonists and Noncardiometabolic Outcomes: An Umbrella Review of Meta-Analyses

**DOI:** 10.1001/jamanetworkopen.2026.4722

**Published:** 2026-03-31

**Authors:** Kaijie Yang, Changyuan Liu, Qiqiang Guo, Yongze Li

**Affiliations:** 1 Department of Endocrinology and Metabolism and the Institute of Endocrinology, National Health Commission Key Laboratory of Diagnosis and Treatment of Thyroid Diseases, First Hospital of China Medical University, Shenyang, China; 2 The College of Basic Medical Science, Health Sciences Institute, China Medical University, Shenyang, China

## Abstract

**Importance:**

Glucagon-like peptide-1 receptor agonists (GLP-1 RAs) are foundational therapies for type 2 diabetes and obesity. Beyond established cardiometabolic benefits, GLP-1 RAs’ associations with noncardiometabolic outcomes remain uncertain.

**Objective:**

To evaluate the associations between GLP-1 RAs and noncardiometabolic outcomes, and to appraise the certainty and credibility of the supporting evidence.

**Data Sources:**

A systematic search of PubMed, Web of Science, Embase, Scopus, and the Cochrane Database of Systematic Reviews was conducted from database inception to January 15, 2026.

**Study Selection:**

Eligible studies were systematic reviews with meta-analyses of randomized clinical trials evaluating GLP-1 RAs and outcomes beyond glycemic control, weight management, and major cardiorenal end points.

**Data Extraction and Synthesis:**

This umbrella review adhered to the Preferred Reporting Items for Systematic Reviews and Meta-Analyses reporting guideline. For each eligible meta-analysis, relevant data were extracted by 2 independent reviewers at both the meta-analysis level and individual study level. Data were reanalyzed using random-effects models to estimate odds ratios (ORs) and 95% CIs.

**Main Outcomes and Measures:**

The primary outcomes were noncardiometabolic outcomes across gastrointestinal, adverse event (AE), cancer, fracture, respiratory, neurologic, psychiatric, hepatic, and endocrine domains. Methodological quality was appraised via AMSTAR 2 (A Measurement Tool to Assess Systematic Reviews). Evidence certainty was categorized using the GRADE (Grading of Recommendations Assessment, Development and Evaluation) framework and prespecified credibility criteria.

**Results:**

A total of 60 meta-analyses representing 116 unique adverse health outcomes were included, comprising 1751 randomized clinical trials and 3 580 616 participants. The study populations primarily involved people with type 2 diabetes (43 [71.7%]) and obesity (20 [33.3%]), with follow-up durations ranging from 3 months to 5.4 years or longer. The most consistent signals were for gastrointestinal AEs, with higher odds of nausea (OR, 2.47 [95% CI, 1.84-3.34]; GRADE: high quality of evidence), vomiting (OR, 2.78 [95% CI, 1.91-4.06]; GRADE: moderate quality of evidence), and diarrhea (OR, 1.94 [95% CI, 1.52-2.49]; GRADE: high quality of evidence), although between-study heterogeneity and 95% prediction intervals suggested residual uncertainty. Infection-related outcomes suggested possible protective associations, particularly for serious infections (OR, 0.89 [95% CI, 0.87-0.92]; GRADE: high quality of evidence), and a suggestive association was observed for incident respiratory disease (OR, 0.85 [95% CI, 0.80-0.92]). Other outcomes, including gastrointestinal disease and biliary events (eg, gallbladder or biliary disease: OR, 1.34 [95% CI, 1.16-1.55]), did not meet stringent credibility thresholds and should be considered exploratory.

**Conclusions and Relevance:**

In this umbrella review of meta-analyses, evidence for most noncardiometabolic outcomes associated with GLP-1 RAs was of lower certainty. Potential safety signals were observed for gastrointestinal AEs, while suggested protective associations with respiratory diseases and serious infections require further confirmation.

## Key Points

**Question:**

What are the associations between glucagon-like peptide-1 receptor agonists (GLP-1 RAs) and noncardiometabolic outcomes, and how credible is the available evidence?

**Findings:**

In this umbrella review of 60 meta-analyses of 1751 randomized clinical trials involving approximately 3 million participants, the most consistent associations with GLP-1 RAs were increased gastrointestinal adverse events (including nausea and vomiting). Suggested protective associations were reported for serious infections and respiratory disease, while possible associations with pancreatitis and gallbladder or biliary disease remained exploratory.

**Meaning:**

This review observed signals of an association between GLP-1 RAs and lower risks of respiratory disease and serious infections; however, the credibility of the evidence was limited and require further confirmation.

## Teaser

This meta-analysis evaluates the reported outcomes of glucagon-like peptide-1 (GLP-1) receptor agonists beyond glycemic control, weight management, and cardiorenal protection and assesses the credibility of the evidence.

## Introduction

Since their introduction in the early 21st century, glucagon-like peptide-1 receptor agonists (GLP-1 RAs) have emerged as cornerstone therapies for type 2 diabetes.^1^ Their clinical value is now widely recognized to extend well beyond glycemic control,^2^ with the past decade witnessing a paradigm shift in their role as central agents in the management of metabolic diseases.^3^ In weight management, GLP-1 RAs demonstrate exceptional efficacy, achieving reductions of up to 14.03 kg,^4^ and have been approved for the treatment of obesity.^5^

Robust evidence also supports their cardiovascular and kidney benefits. A meta-analysis of 8 cardiovascular outcomes trials involving 60 080 participants reported a 13% relative reduction in cardiovascular mortality and a 17% reduction in broad renal composite outcomes.^6^ Semaglutide has been shown to lower the risk of major adverse cardiovascular events by 26%,^7^ and GLP-1 RAs have been associated with substantial reductions in composite kidney outcomes among patients with chronic kidney disease (odds ratio [OR], 0.85; 95% CI, 0.77-0.94).^8^ Collectively, these high-quality data have firmly established weight loss and cardiorenal protection as hallmark nonglycemic benefits of GLP-1 RAs. However, their potential role in a broader spectrum of health outcomes remain incompletely characterized.

To address this gap, we conducted an umbrella review, integrating evidence from randomized clinical trial (RCT)–based systematic reviews and meta-analyses. Our aim was to evaluate the associations between GLP-1 RAs and noncardiometabolic outcomes and to appraise the certainty and credibility of the supporting evidence.

## Methods

This umbrella review systematically synthesized and evaluated evidence from published systematic reviews and meta-analyses. We followed the Preferred Reporting Items for Systematic Reviews and Meta-Analyses (PRISMA) reporting guideline.^9^ The study protocol was prospectively registered on PROSPERO (identifier: CRD420251114177).^10^

### Literature Search and Selection Criteria

A comprehensive search strategy (eTable 1 in Supplement 1) was systematically implemented to identify peer-reviewed meta-analyses of RCTs that examined the association between GLP-1 RAs and noncardiometabolic clinical outcomes. Our search spanned from database inception through January 15, 2026, and included PubMed, Web of Science, Embase, Scopus, and the Cochrane Database of Systematic Reviews.

Eligibility criteria were defined according to the Population, Intervention, Comparisons, Outcomes, and Study (PICOS) reporting framework (eTable 2 in Supplement 1),^11^ and we included meta-analyses of RCTs evaluating the noncardiometabolic outcomes of GLP-1 RAs, defined as clinical end points beyond their established benefits in glycemic control, weight management, and cardiorenal protection. For outcomes captured as adverse events (AEs) in the underlying RCTs and synthesized in the source meta-analyses, we extracted outcome data as reported and classified them as AE-based. We did not redefine, readjudicate, or harmonize AE definitions across trials.

### Study Selection and Data Extraction

Selection of systematic reviews and meta-analyses was independently conducted by 2 investigators (K.Y. and C.L.) in a 2-stage process (combined title and abstract screening, and full-text evaluation). Discrepancies were resolved via discussion to reach consensus, with another author (Y.L.) being consulted for arbitration if necessary. Data extraction was also performed independently by the same investigators (K.Y. and C.L.) using a standardized form; disagreements were resolved by consensus or third-author adjudication. For overlapping meta-analyses addressing identical outcomes, the most recent or largest study was prioritized, which is consistent with established methods for evidence synthesis.^12,13,14^

Data extraction was systematically performed using Microsoft Excel, version 16.0 (Microsoft Corp). For each eligible meta-analysis, relevant data were extracted at both the meta-analysis level and individual study level, including the first author’s name, publication year, number of included studies, outcomes investigated, sample size, and event counts. The extracted data encompassed several outcomes. Where available, we also captured related treatment characteristics, with emphasis on agent class (particularly tirzepatide), dose, and treatment duration.

### Credibility and Quality Assessment of Evidence and Methods

According to established criteria used in previously published umbrella reviews,^15,16^ we classified the credibility of evidence using prespecified umbrella-review criteria (eTable 3 in Supplement 1) and categorized associations as convincing (class I), highly suggestive (class II), suggestive (class III), weak (class IV), or nonsignificant. To support a more cautious interpretation of findings, we highlighted only associations with a summary *P* < .001 and a 95% prediction interval (PI) excluding the null. Nominally significant findings (*P* < .05) that did not meet these thresholds were reported cautiously.

We used GRADE (Grading of Recommendations Assessment, Development and Evaluation) to evaluate the quality of evidence for each unique pooled analysis.^17^ GRADE categorized the overall quality of evidence for each outcome as high, moderate, low, or very low (eTable 4 in Supplement 1).

We evaluated the methodological quality of the included systematic reviews with meta-analyses using the AMSTAR 2 (A Measurement Tool to Assess Systematic Reviews) instrument (eTable 5 in Supplement 1).^18^ This tool comprises 16 items designed to appraise the rigor of meta-analytical methods, with particular emphasis on critical domains that may affect the reliability of the findings. Consistent with recommendations from recent literature, we applied AMSTAR 2 to conduct a qualitative appraisal, taking into account the potential implications of low ratings for individual items, especially within the critical domains outlined in eTable 5 in Supplement 1. Rather than assigning numerical scores or generating an overall summary rating, we focused on evaluating the methodological strengths and weaknesses across these domains.^18^

### Statistical Analysis

#### Summary Effect Estimation

All outcomes were dichotomous. We extracted trial-level data from the included RCTs and reanalyzed them using a random-effects model. The between-study variance (τ^2^) was estimated using the restricted maximum likelihood estimator.^19^ Treatment effects were expressed as ORs, with 95% CIs derived using Wald-type intervals based on the standard normal distribution. Event counts for each trial contributing to each outcome are provided in eTable 4 in Supplement 1. This strategy ensured methodological consistency across outcomes, regardless of the analytic approach used in the original meta-analyses. All statistical tests were 2-sided, and statistical significance was defined as *P* < .05. Analyses were performed using R, version 4.4.2 (R Foundation for Statistical Computing).

Between-study heterogeneity was reassessed using the *I*^2^ statistic, and 95% PIs were calculated to estimate the potential distribution of true associations in future studies. Given that all outcomes were dichotomous, publication bias was assessed using the Harbord test, while small-study advantages and excess significance bias were also examined as part of the reanalysis (eMethods in Supplement 1).

#### Overlap Across Reviews

Overlap of primary RCTs across reviews was quantified using corrected covered area (CCA). CCA ranges from 0% (no overlap) to 100% (complete overlap) and was interpreted as slight (0%-5%), moderate (6%-10%), high (11%-15%), or very high (>15%).^20^ Outcome-specific CCA visualizations are presented in eFigures 1 to 3 in Supplement 1.

#### Sensitivity Analyses

Given the low event rates and limited follow-up in cancer outcomes, we performed prespecified rare-event sensitivity analyses. Trials with 0 events in both arms (double-zero trials) were excluded from OR pooling because they contributed no information to relative associations. For trials with 0 events in 1 arm (single-zero trials), we applied the Mantel-Haenszel method with a treatment arm continuity correction and additionally conducted the Peto method as an alternative rare-event approach. Robustness of pooled estimates was further evaluated using leave-one-trial-out (LOTO) analyses (eTable 6 in Supplement 1).

## Results

**Figure 1.**
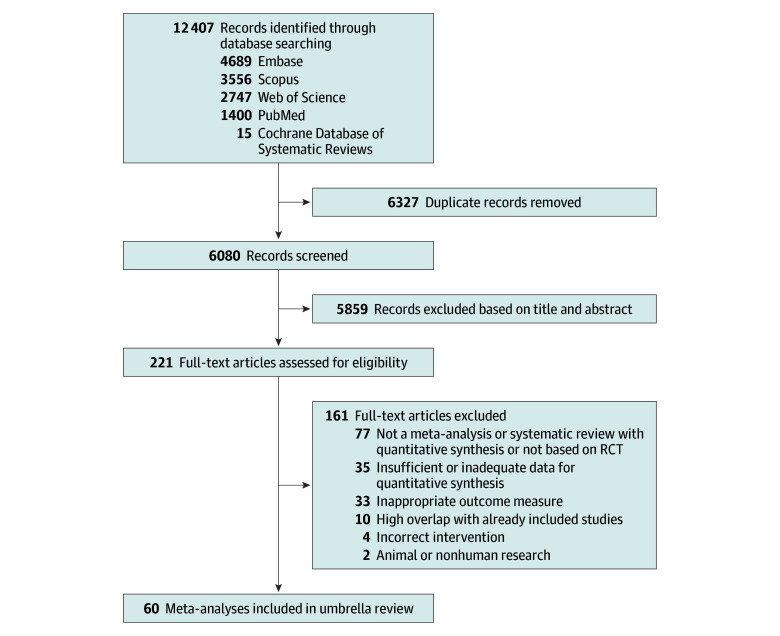
Flowchart of Systematic Search and Selection Process RCT indicates randomized clinical trial.

Our systematic literature search initially identified 12 407 records after the removal of duplicates. Following title and abstract screening, 221 full-text articles were thoroughly evaluated for eligibility, resulting in the preliminary inclusion of 70 review articles. We subsequently excluded 10 reviews due to duplication of primary study data. Ultimately, a total of 60 meta-analyses^21,22,23,24,25,26,27,28,29,30,31,32,33,34,35,36,37,38,39,40,41,42,43,44,45,46,47,48,49,50,51,52,53,54,55,56,57,58,59,60,61,62,63,64,65,66,67,68,69,70,71,72,73,74,75,76,77,78,79,80^ comprising 1751 randomized clinical trials and 3 580 616 participants were included in the final analysis (Figure 1 and Table). A comprehensive list of the excluded studies is provided in eTable 7 in Supplement 1.

**Table.** Characteristics of the Systematic Reviews With Meta-Analyses Included in the Umbrella Review

Source	Study population diagnosis	Population age group, y	Associations, No.	Intervention	Comparator	Outcomes	Follow-up duration
Tan et al,^40^ 2025	T2D	≥18	25	GLP-1 RA	Placebo or active control	Fracture incidence	12-156 wk
Zhang et al,^39^ 2025	T2D	51.0-64.6	44	GLP-1 RA	Placebo or active control	Fracture risk	24-156 wk
Cheng et al,^43^ 2019	T2D	NA	38	GLP-1 RA	Placebo or active control	Fracture risk	NA
Su et al,^42^ 2015	Diabetes or nondiabetes	45.9-59.5	16	GLP-1 RA	Placebo or active control	Incidence of bone fractures	12-104 wk
Mabilleau et al,^41^ 2014	T2D	Mean (SD): 56.4 (1.9)	7	GLP-1 RA	Placebo or active control	Incidence of bone fractures	26-104 wk
Zhang et al,^77^ 2024	T2D or obesity	NA	39	GLP-1 RA	Placebo or active control	Asthma	NA
Yu et al,^78^ 2023	T2D, overweight or obesity	46.0-74.2	28	GLP-1 RA; GLP-1 RA can also be used as add-on therapy	Placebo or active control	Respiratory diseases	24 wk to 5.4 y
Wei et al,^79^ 2021	T2D	NA	7	GLP-1 RA	Placebo	Respiratory disorders	NA
Seminer et al,^71^ 2025	T2D	60.3-66.2	10	GLP-1 RA	Placebo	All-cause dementia	9-45.6 mo
Sindhu et al,^72^ 2024	T2D	60.3-66.2	8	GLP-1 RA	Placebo	Epilepsy or seizure AEs	15.9-64.8 mo
Tang et al,^73^ 2023	T2D	62-72	8	GLP-1 RA	Placebo	Parkinson disease	1.6-5.4 y
Chen et al,^74^ 2025	T2D or obesity	14.0-66.2	25	GLP-1 RA	Placebo or active control for T2D, other weight-loss medications or placebo for obesity	Suicidal behavior	NA
Ebrahimi et al,^75^ 2025	Diabetes, overweight or obesity	Mean (SD): 59.5 (XX)	27	GLP-1 RA	Placebo	Self-harm and/or suicide-related AEs	Mean (SD): 59.5 (9) wk
Alves et al,^66^ 2012	T2D	NA	37	GLP-1 RA	Placebo or active control	Effect estimates on acute pancreatitis or cancer	NA
Mantovani et al,^78^ 2025	Overweight or obesity, MASLD or MASH	≥18	13	GLP-1 RA	Placebo	MASH resolution without worsening of liver fibrosis	24-72 wk for RCTs with liver biopsy data and RCTs using MRI-based data
Chiang et al,^67^ 2025	T2D, overweight or obesity, or MASH or MASLD	NA	55	GLP-1 RA	Placebo	At least 1 GI or biliary conditions	26-238 wk
Safwan et al,^58^ 2025	Obesity: BMI ≥30 (or ≥27 with comorbidity)	44-49	13	GLP-1 RA	Placebo	GI AEs, hepatic AEs, new cases of pancreatitis, and gallbladder and biliary disorders	12-104 wk
Cao et al,^57^ 2020	T2D	NA	4	GLP-1 RA	Placebo	Acute pancreatitis and pancreatic cancer	1.3-5.4 y
Masson et al,^68^ 2024	NA	NA	21	Semaglutide	Placebo	Incidence of acute pancreatitis	≥3 mo
Cao et al,^52^ 2019	T2D	NA	37	GLP-1 RA	Non-GLP-1 RA active comparator and/or placebo	Overall incidence of any cancer	At least 52 wk
Wang et al,^59^ 2024	T2D or obesity	NA	8	GLP-1 RA	Placebo or active comparator	GI AEs	NA
Dutta et al,^35^ 2022	MASLD and/or T2D	48-64.6	4	Semaglutide	Placebo or active control	Liver enzymes, hepatic ultrasonography scores, liver stiffness, and any AEs	NA
Dutta et al,^30^ 2021	T2D	NA	NA	Tirzepatide	Placebo or any other active comparator	Changes in HbA_1c_	NA
He et al,^62^ 2022	NA	Mean (SD): 57.8 (6.2)	76	GLP-1 RA	Placebo or active control	Composite of gallbladder or biliary diseases	NA
Hu et al,^80^ 2022	T2D, T1D, or prediabetes, or overweight or obesity	41.6-66.2	45	GLP-1 RA	Placebo or active control	Overall thyroid disorders	≥24 wk
Duchemin et al,^51^ 2025	Diabetes, overweight or obesity	NA	44	GLP-1 RA	Non-GLP-1 RA active comparator and/or placebo	Thyroid cancer	NA
Ko et al,^55^ 2026	T2D or overweight or obesity	≥18	48	GLP-1 RA	Placebo	Thyroid, pancreatic, colorectal, gastric, esophageal, liver, gallbladder, breast, ovarian, endometrial, or kidney cancer; multiple myeloma; or meningioma	24-281 wk
Rao et al,^56^ 2025	T2D	52.0-71.0	30	GLP-1 RA	Placebo	Bladder cancer	68-282 wk
Monami et al,^64^ 2017	T2D	≥18	113	GLP-1 RA	Non-GLP-1 RA active comparator and/or placebo	Pancreatitis, pancreatic cancer, and cholelithiasis	12-208 wk
Monami et al,^65^ 2014	T2D	NA	41	GLP-1 RA	Placebo or active control	Pancreatitis	>12 wk
Wu et al,^44^ 2025	T2D or obesity	Mean (SD): 62.4 (9.9)	5	GLP-1 RA	Placebo or active control	Esophageal cancer	52-156 wk
Figlioli et al,^53^ 2024	No restrictions	Mean (SD): 59.7 (XX)	90	GLP-1 RA	Placebo or active control	GI cancer	≥24 wk
Muhammed et al,^60^ 2024	T2D	NA	54	Antidiabetic medications, alone or in combination	Placebo or active control	Pancreatitis and pancreatic cancer	NA
Nagendra et al,^46^ 2023	T2D	NA	37	Semaglutide	Placebo or active control	Pancreatic cancers and thyroid cancers, any other types of malignant neoplasms, or any other severe AEs	NA
Nreu et al,^61^ 2023	T2D	NA	43	GLP-1 RA	Placebo or active control	Pancreatitis	≥52 wk
Nreu et al,^63^ 2020	NA	NA	43	GLP-1 RA	Placebo or active control	Cholelithiasis	≥52 wk
Piccoli et al,^47^ 2021	T2D, prediabetes, obesity or overweight, and/or metabolic syndrome	NA	52	GLP-1 RA	Non-GLP-1 RA antidiabetic or weight loss medications or placebo	Breast cancer	24 wk to 7.5 y
Silverii et al,^76^ 2024	T2D or obesity	≥18	31	GLP-1 RA	Placebo or active control	Psychiatric disorder, suicidal behavior, and depression and anxiety	52-198 wk
Silverii et al,^45^ 2025	Diabetes or obesity	45-69	50	GLP-1 RA	Placebo or any comparator, except other GLP-1 RAs and GLP-1/GIP and GLP-1/glucagon dual agonists	Incidence of any malignant neoplasia	52-281 wk
Liu et al,^48^ 2019	T2D	NA	34	GLP-1 RA	Placebo or active control	All types of malignant tumors	24-198 wk
Pinto et al,^54^ 2019	T2D	Mean (SD): 58 (4.3)	12	GLP-1 RA	Placebo or active control	Pancreatic cancer	1-3.5 y
Guo et al,^49^ 2016	T2D	NA	26	GLP-1 RA	Placebo or active control	Cancer	16-156 wk
Silverii et al,^50^ 2024	T2D or obesity	45-66	26	GLP-1 RA	Placebo or active control	Overall thyroid cancer	53-281 wk
Arrowaili et al,^23^ 2025	Metabolic bariatric surgery and suboptimal clinical response or weight recurrence	>40	5	GLP-1 RA	Placebo or no drugs	Metabolic outcomes and AEs	6-24 mo
Badran et al,^28^ 2025	Parkinson disease	57-62	4	GLP-1 RA	Placebo	Effectiveness and safety	3-24 mo
Han et al,^22^ 2025	No restriction	Mean (SD): 56.2 (5.87)	136	GLP-1 RA	Placebo or active control	Serious AEs of infections and infestations	12-225 wk
Helal et al,^26^ 2025	Parkinson disease	NA	5	GLP-1 RA	Placebo or usual care or no treatment	Motor impairment in Parkinson disease, motor experiences of daily living, and incidence of GI and systemic adverse effects	NA
Huang et al,^27^ 2025	MASLD	NA	10	GLP-1 RA	Placebo or active control	Occurrence of various types of AEs	24-72 wk
Khan et al,^36^ 2025	Parkinson disease	Mean (SD): 60.8 (8.6)	5	GLP-1 RA	Placebo or standard treatment	Scores in MDS-UPDRS parts I-IV; secondary outcomes included AEs	NA
Mostafa et al,^34^ 2025	Any age with IBS	18-70	4	GLP-1 RA	Placebo or standard treatment	Pain and symptoms of IBS relief; frequency of adverse effects	24 h to 3 mo
Sillassen et al,^37^ 2025	T2D or overweight, CKD, HFpEF, or MASH	60-65	48	Semaglutide	Placebo	Serious AEs	NA
Taj et al,^25^ 2026	Diabetes and/or obesity	49-63.5	38	GLP-1 RA	Placebo or no drugs	Dermatologic reactions	5-156 wk
Hu et al,^33^ 2023	Females with PCOS	26-32	9	Exenatide alone or plus metformin	Exenatide vs metformin	Pregnancy rate, sex hormone levels, change in body weight, and metabolic disorders and safety assessed by incidence of adverse effects	12-24 wk
Ye et al,^21^ 2023	PCOS	20-40	9	Exenatide	Exenatide vs metformin	Pregnancy rate, ovulation rate, BMI, homeostasis model assessment of insulin resistance, and AEs	12-25 wk
Wen et al,^69^ 2025	GLP-1 RA use	14.4-68	62	GLP-1 RA	Placebo or active control	Pancreatitis and pancreatic cancer	1-198 wk
Konwar et al,^32^ 2022	Overweight or obesity	≥18	14	Liraglutide	Placebo or active control	Overall effect of liraglutide in weight reduction	20-160 wk
Li et al,^31^ 2021	T2D	54-71	10	Oral semaglutide	Placebo or active control	Any specific outcomes and safety end points	>12 wk
Patoulias et al,^24^ 2022	T2D	NA	6	GLP-1 RA	Placebo	Respiratory tract infections and ARDS	NA
Dimitrios et al,^29^ 2020	T1D	NA	6	Liraglutide	Placebo	Vomiting and nausea	12-52 wk
Avgerinos et al,^38^ 2020	T2D	47-68.6	6	GLP-1 RA, oral semaglutide (3, 7, 14 mg daily)	Placebo	Vomiting and nausea	12-72 wk

Abbreviations: AE, adverse event; ARDS, acute respiratory distress syndrome; BMI, body mass index (calculated as weight in kilograms divided by height in meters squared); CKD, chronic kidney disease; GI, gastrointestinal; GIP, glucose-dependent insulinotropic polypeptide; GLP-1 RA, glucagon-like peptide-1 receptor agonist; HbA_1c_, hemoglobin A_1c_; HFpEF, heart failure with preserved ejection fraction; IBS, irritable bowel syndrome; MASH, metabolic dysfunction–associated steatohepatitis; MASLD, metabolic dysfunction–associated steatotic liver disease; MDS-UPDRS, Movement Disorder Society Unified Parkinson Disease Rating Scale; MRI, magnetic resonance imaging; NA, not applicable; PCOS, polycystic ovary syndrome; RCT, randomized clinical trial; T1D, type 1 diabetes; T2D, type 2 diabetes.

### Study Characteristics

Across all 60 meta-analyses, 116 adverse health outcomes were associated with GLP-1 RAs exposure (eTables 3 and 8 in Supplement 1). The outcomes were most frequently assessed in meta-analyses of AEs (18 [30.0%])^21,22,23,24,25,26,27,28,29,30,31,32,33,34,35,36,37,38^ followed by cancer outcomes (14 [23.3%])^44,45,46,47,48,49,50,51,52,53,54,55,56,57^ and gastrointestinal outcomes (12 [20.0%]).^58,59,60,61,62,63,64,65,66,67,68,69^ The remaining domains included fracture (5 [8.3%]),^39,40,41,42,43^ respiratory (3 [5.0%]),^77,78,79^ neurological (3 [5.0%]),^71,72,73^ psychiatric (3 [5.0%]),^74,75,76^ hepatic (1 [1.7%]),^70^ and endocrine or metabolic outcomes (thyroid diseases: 1 [1.7%]^80^). The majority of these meta-analyses (56 [93.3%])^21,22,23,24,25,26,27,28,29,30,31,32,33,34,35,36,37,38,39,40,43,44,45,46,47,48,49,50,51,52,53,54,55,56,57,58,59,60,61,62,63,64,67,68,69,70,71,72,73,74,75,76,77,78,79,80^ were published within the past decade, reflecting the recent surge of research interest in this area. The Table outlines the core characteristics of each meta-analysis, including study population, age range, and follow-up duration. Most meta-analyses focused on individuals with diabetes (44 [73.3%]),^24,25,27,29,30,31,35,37,38,39,40,41,42,43,44,45,46,47,48,49,50,51,52,54,55,56,57,59,60,61,64,65,66,67,71,72,73,74,75,76,77,78,79,80^ one-third involved populations with obesity (20 [33.3%]).^23,25,32,37,44,45,47,50,51,55,58,59,67,70,74,75,76,77,78,80^ Follow-up periods varied substantially, ranging from 3 months to 5.4 years or longer.

### Summary Findings

We assessed overlap of primary RCTs across included meta-analyses using an RCT-by-review matrix and quantified it using CCA after removing fully overlapping reviews. Liver-related^70^ and endocrine-related^80^ outcomes each included only 1 systematic review and therefore did not allow within-category overlap estimation. For the remaining outcome categories, CCA values were 1.0% for AEs,^21,22,23,24,25,26,27,28,29,30,31,32,33,34,35,36,37,38^ 3.8% for respiratory,^77,78,79^ 15.0% for fracture,^39,40,41,42,43^ 4.7% for cancer,^44,45,46,47,48,49,50,51,52,53,54,55,56,57^ 5.3% for digestive,^58,59,60,61,62,63,64,65,66,67,68,69^ 11.9% for neurologic,^71,72,73^ and 4.6% for psychiatric.^74,75,76^ Overall, most categories showed slight overlap (CCA ≤5%), whereas neurologic and fracture outcomes indicated high overlap (11.9% and 15.0%, respectively) (eFigures 1-3 in Supplement 1).

Gastrointestinal symptoms showed the most consistent signals among AE outcomes, although between-study heterogeneity and 95% PIs suggested residual uncertainty. Among gastrointestinal events, GLP-1 RAs were most consistently associated with higher odds of nausea^28^ (OR, 2.47 [95% CI, 1.84-3.34]; GRADE: high quality of evidence; weak association [class IV]), vomiting^38^ (OR, 2.78 [95% CI, 1.91-4.06]; GRADE: moderate quality of evidence; highly suggestive association [class II]), and diarrhea^38^ (OR, 1.94 [95% CI, 1.52-2.49]; GRADE: high quality of evidence; highly suggestive association [class II]). Infection-related outcomes suggested a possible association with lower odds, particularly for serious infections^22^ (OR, 0.89 [95% CI, 0.87-0.92]; GRADE: high quality of evidence; convincing association [class I]).

Across fracture, respiratory, neurological, psychiatric, and endocrine outcomes, most pooled estimates did not reach the prespecified high-credibility threshold, but several outcomes showed possible signals. These outcomes included lower odds of fracture^43^ (OR, 0.67 [95% CI, 0.52-0.87]; *P* = .003), incident respiratory disease^78^ (OR, 0.85 [95% CI, 0.80-0.92]; *P* < .001), and all-cause dementia^71^ (OR, 0.55 [95% CI, 0.35-0.87]; *P* = .01) as well as higher odds of thyroid disease^80^ (OR, 1.27 [95% CI, 1.02-1.59]; *P* = .04); overall certainty was limited. Liver-related evidence was sparse, with only 1 association suggesting an exploratory signal for metabolic dysfunction–associated steatohepatitis resolution^70^ (OR, 3.39 [95% CI, 2.63-4.36]; *P* < .001) (eTable 4 in Supplement 1).

Gastrointestinal disease outcomes did not meet prespecified stringent credibility criteria, with 95% PIs generally overlapping the null. Gastroesophageal reflux disease showed a possible signal toward higher odds^67^ (OR, 2.19 [95% CI, 1.65-2.90]; *P* < .001), and gallbladder or biliary diseases suggested a possible signal^62^ (OR, 1.34 [95% CI, 1.16-1.55]; *P* < .001); however, 95% PIs were close to the null. Other gastrointestinal and biliary-specific outcomes were generally inconclusive and should be considered exploratory (eTable 4 in Supplement 1).

Cancer outcomes showed no associations meeting the prespecified credibility thresholds despite several nominal signals. Colorectal cancer showed a nominal signal toward higher odds^45^ (OR, 1.24 [95% CI, 1.00-1.54]; *P* = .049; weak association [class IV]). Thyroid cancer showed an imprecise, nonstatistically significant estimate compatible with higher odds^45^ (OR, 1.43 [95% CI, 0.95-2.13]; *P* = .08). Only 1 study further examined thyroid cancer subtypes and reported an imprecise estimate for papillary thyroid cancer^55^ (OR, 1.30 [95% CI, 0.68-2.52]). Pancreatic cancer showed a nominal signal toward lower odds^46^ (OR, 0.51 [95% CI, 0.30-0.85]; *P* = .01), but this finding remained exploratory (eTable 4 in Supplement 1).

### Rare-Event Methods

Sensitivity analysis of rare-event methods revealed the instability of initial signals. While the overall direction of treatment effect remained largely consistent, the LOTO analysis indicated fragility: the direction of treatment effect flipped in approximately 9 of 39 outcomes,^44,52,53,54,55,57,69^ and statistical significance was lost in 6 of 39 outcomes^46,51,52,55,60,80^ after the removal of a single trial (eTable 6 in Supplement 1). eTable 9 in Supplement 1 presents subgroup analyses by GLP-1 RA type; the most commonly evaluated agents were semaglutide and liraglutide. Subgroup analyses for tirzepatide, GLP-1 RA dose, and treatment duration included few studies^30,34,39,41,43,55,58,67,68,77^ and should be interpreted as exploratory (eTables 10 and 11 in Supplement 1).

### Credibility Criteria, GRADE, and AMSTAR 2

**Figure 2.**
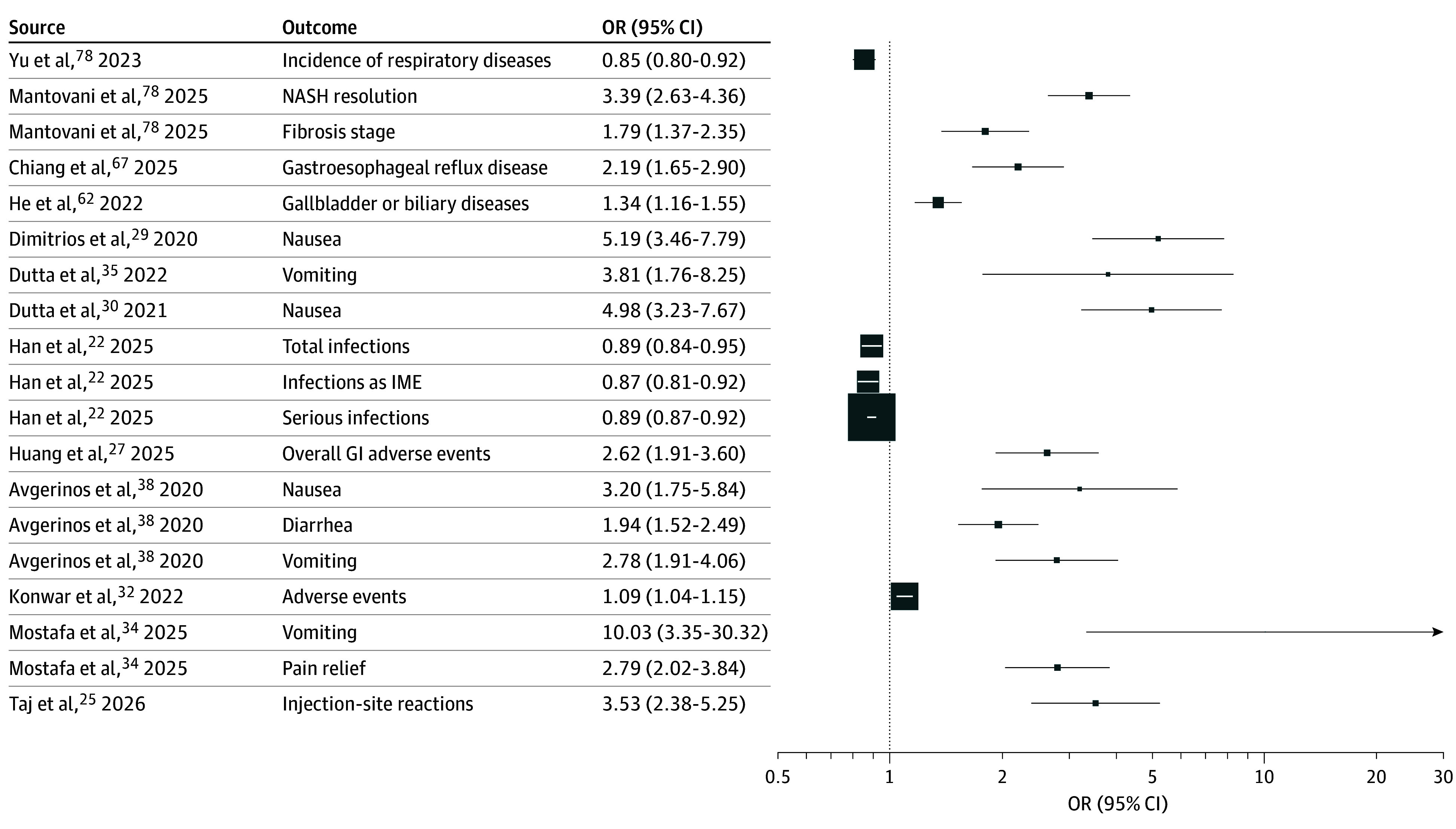
Forest Plot of Glucagon-Like Peptide-1 Receptor Agonist (GLP-1 RA) Associations With Selected Outcomes With Class I to III Credibility Error bars represent 95% CIs. Larger squares represent studies with larger sample sizes. Values to the right of 1 indicate increased odds, and values to the left of 1 indicate decreased odds associated with GLP-1 RAs. GI indicates gastrointestinal; IME, important medical event; MASH, metabolic dysfunction-associated steatohepatitis (formerly nonalcoholic steatohepatitis [NASH]); and OR, odds ratio.

eTable 4 in Supplement 1 summarizes the associations between GLP-1 RAs and several outcomes, along with the corresponding GRADE ratings and strength-of-evidence classifications. Figure 2 presents a forest plot of GLP-1 RA associations with selected outcomes. For example, GLP-1 RAs were associated with an increased risk of nausea^29^ (OR, 5.19 [95% CI, 3.46-7.79]).

Under AMSTAR 2, all 60 meta-analyses^21,22,23,24,25,26,27,28,29,30,31,32,33,34,35,36,37,38,39,40,41,42,43,44,45,46,47,48,49,50,51,52,53,54,55,56,57,58,59,60,61,62,63,64,65,66,67,68,69,70,71,72,73,74,75,76,77,78,79,80^ clearly specified PICO elements (item 1) and used appropriate statistical methods (critical item 11), and 57 meta-analyses (95.0%)^21,22,23,24,25,26,27,28,29,30,31,32,33,34,35,36,37,38,39,40,41,42,43,44,45,46,47,48,50,51,52,53,54,55,57,58,59,60,61,62,63,64,65,66,67,68,69,70,71,72,73,74,75,76,77,78,80^ considered risk of bias when interpreting results (critical item 9). However, key methodological limitations were common in several critical domains, including lack of a prespecified protocol (critical item 2), failure to provide a list of excluded studies with justification (critical item 7), and inadequate consideration of risk of bias in primary studies when interpreting findings (critical item 13). In addition, 41 meta-analyses (68.3%)^21,22,23,24,25,26,27,28,29,32,33,34,35,36,39,41,42,44,45,48,50,52,53,54,55,56,57,59,60,63,64,65,68,69,70,73,74,75,77,79,80^ did not report funding sources of included studies (item 10) or assess the potential implication of individual study bias for the synthesized results (item 12) (eTable 5 in Supplement 1).

## Discussion

This comprehensive assessment revealed a complex profile for GLP-1 RAs across noncardiometabolic outcomes. We identified relatively robust signals for gastrointestinal AEs, particularly nausea,^28,29,32,34,35,38^ vomiting,^26,28,35,36,38^ and diarrhea.^35,38^ Potential protective associations were observed for respiratory disease^78^ and all-cause dementia,^71^ alongside a possible reduced risk of fractures.^43^ However, several associations did not meet the prespecified high-credibility thresholds and remained exploratory.^26,35,45,58,59,62^ Evidence regarding cancer risks was inconclusive, with no persistent associations in sensitivity analyses conducted under stringent credibility criteria.

Consistent with the findings of our study, emerging evidence^81,82^ corroborates the potential of GLP-1 RAs to mitigate the risk of dementia. The neuroprotective properties of GLP-1 RAs are likely attributable to their multifaceted mechanisms, encompassing broad anti-inflammatory properties, enhancement of cerebrovascular function, and attenuation of amyloid-β deposition and tau hyperphosphorylation. GLP-1 receptors are expressed in key cerebral regions, such as the hippocampus and cortex, and their activation has been demonstrated to bolster synaptic plasticity and neuronal survival.^81^ This association has been further substantiated by large-scale epidemiological investigations.^82^

Regarding respiratory outcomes, our analysis was constrained by a limited number of studies, revealing a reduction only in the overall risk of respiratory diseases, with no significant findings for specific disease subcategories. Nevertheless, existing research suggests that the anti-inflammatory pathways modulated by GLP-1 RAs may confer benefits in conditions characterized by airway inflammation and oxidative stress, such as asthma and chronic obstructive pulmonary disease.^33^ Furthermore, the substantial weight reduction induced by GLP-1 RAs may indirectly contribute to a lower overall risk of respiratory morbidity by improving respiratory mechanics and alleviating obesity-related complications, including obstructive sleep apnea.^78,83^

Our analysis found no robust evidence to support an increased risk of cancer associated with GLP-1 RA use. Although nominal signals were observed in preliminary analyses, these associations did not meet prespecified credibility thresholds and were not substantiated in sensitivity analyses, suggesting they may be attributed to biases rather than true causation.

Regarding thyroid cancer, while preclinical data indicating GLP-1 RA–induced C-cell hyperplasia in rodents have raised long-standing concerns,^84^ the relevance of these findings to humans remains unconfirmed.^47^ The absence of a robust signal in our comprehensive assessment supports the hypothesis that the human thyroid differs substantially in GLP-1 receptor expression and responsiveness. Furthermore, only 1 study has explored the subtypes of thyroid cancer, presenting an OR for papillary thyroid cancer of 1.30 (95% CI, 0.68-2.52), which underscores the considerable uncertainty surrounding these findings.^55^

The gastrointestinal adverse effects of GLP-1 RAs are well-recognized, with regulatory bodies maintaining warnings regarding the potential risk of pancreatitis. Our study shows an elevated likelihood of pancreatitis and gallbladder or biliary diseases. However, the scientific consensus on the causal association between GLP-1 RAs and pancreatitis remains unsettled. A 2025 propensity score–matched analysis using a large US database reported no increased risk of pancreatitis after adjusting for multiple comorbidities.^85^ A 2026 safety update referenced by UK regulatory bodies highlighted rare but fatal cases of pancreatitis associated with GLP-1 RAs.^86^ Although we interpreted our findings as exploratory and hypothesis-generating, these conflicting data and renewed warnings underscore the urgent need for large-scale, high-quality clinical studies with long-term follow-up to definitively resolve this safety signal.

Regarding gallbladder and biliary diseases, the literature presents conflicting evidence on the temporal nature of the risk. Some studies suggest the risk is concentrated within the first 6 months of GLP-1 RA therapy,^87^ whereas others report an increased risk only with longer treatment durations.^62^ Such inconsistencies underscore the necessity for future systematic reviews to incorporate high-quality data on treatment duration and dose to accurately delineate the risk profile.

Furthermore, intriguing evidence is emerging for the potential role of GLP-1 RAs in addictive disorders, such as alcohol use disorder.^88^ Animal studies provide a plausible mechanism, suggesting the role may involve the modulation of alcohol-induced reward and punishment pathways in the brain, possibly via a reduction in alcohol-induced reward and nucleus accumbens–dependent mechanisms.^89^ Given the preliminary nature of this research, more high-quality studies are warranted to substantiate these compelling findings.

Given the current paucity of high-certainty evidence, the findings of this umbrella review are intended primarily for hypothesis-generating purposes and preclude the formulation of robust clinical recommendations or modifications. Specifically, any identified safety signals should prompt nuanced, individualized risk-benefit discussions and targeted monitoring—particularly for symptomatic patients or those with high-risk clinical profiles—rather than routine alterations to GLP-1 RA prescribing patterns or surveillance protocols. Mechanistic investigations and adequately powered prospective trials are essential to validate these preliminary observations before any definitive shifts in clinical practice or guideline updates can be justified.

### Limitations

This study has several limitations. First, as an umbrella review, the scope was restricted to outcomes synthesized in existing meta-analyses, inevitably excluding emerging end points reported only in individual trials or clinical studies. Second, many noncardiometabolic outcomes were captured as AEs rather than prespecified primary end points. These data are subject to heterogeneous definitions, passive ascertainment, and lack of independent adjudication, rendering them susceptible to misclassification and reporting bias. Third, inconsistent reporting in primary trials precluded detailed stratification by dose or treatment duration, potentially masking time-dependent or dose-response relationships. We encourage future studies to report subgroup results in greater detail to enable clinically meaningful meta-analyses. Fourth, considerable primary-study overlap across source meta-analyses was observed; future reviews should rigorously assess redundancy to avoid double-counting bias. Finally, given that some direct associations lack established biological mechanisms, these findings should be interpreted as exploratory and hypothesis-generating rather than causal, requiring confirmation in adequately powered, well-adjudicated prospective studies.

## Conclusions

In this umbrella review of meta-analyses, although GLP-1 RAs have been widely studied across a spectrum of health outcomes, the current evidence base is insufficient for definitive conclusions of high certainty. GLP-1 RAs continue to be a cornerstone of antidiabetic therapy but should not be misconstrued as a panacea. The complex profile that emerged from our findings was characterized by potential neuroprotective and respiratory benefits coexisting with safety signals related to gastrointestinal events, such as nausea and vomiting. These findings reinforce the necessity of individualized therapeutic strategies and sustained clinical vigilance rather than routine practice change.

## Notes

Supplement 1. **eMethods.** Description of Statistical Analysis Methods **eTable 1.** Search Strategy **eTable 2.** Inclusion and Exclusion Criteria **eTable 3.** Criteria for Quality of Evidence Classification **eTable 4.** Summary of Associations Between Glucagon-Like Peptide-1 Receptor Agonists and Several Outcomes, Including Strength of Evidence and GRADE Assessments **eTable 5.** AMSTAR 2 Quality Assessment by Health Outcome Domain **eTable 6.** Sensitivity and Leave-One-Out Analyses of Associations Between Glucagon-Like Peptide-1 Receptor Agonists and Cancer Outcomes **eTable 7.** Studies Excluded from the Meta-Analysis with Rationale **eTable 8.** Outcomes Included in the Umbrella Review **eTable 9.** Summary of Associations Between Glucagon-Like Peptide-1 Receptor Agonists and Selected Outcomes, Stratified by Drug Type **eTable 10.** Summary of Associations Between Tirzepatide and Skeletal, Respiratory, Gastrointestinal, and Cancer Outcomes **eTable 11.** Summary of Associations Between Glucagon-Like Peptide-1 Receptor Agonists and Selected Outcomes, Stratified by Treatment Duration and Dose **eFigure 1.** Overlap Among Systematic Reviews of Adverse Events, Quantified by Corrected Covered Area **eFigure 2.** Overlap Among Systematic Reviews of Gastrointestinal (A) and Cancer (B) Outcomes, Quantified by Corrected Covered Area **eFigure 3.** Overlap Among Systematic Reviews of Neurological(A), Respiratory(B), Fracture(C), and Psychiatric(D) Outcomes, Quantified by Corrected Covered Area
eReferences

Supplement 2.
Data Sharing Statement
