# Supplementary material for: GLP-1 Receptor Agonists and Noncardiometabolic Outcomes: An Umbrella Review of Meta-Analyses
Source: JAMA Netw Open. 2026 Mar 31;9(3):e264722. doi: 10.1001/jamanetworkopen.2026.4722 (PMC13040404; doi:10.1001/jamanetworkopen.2026.4722)
Supplement: Supplement 1. — eMethods. Description of Statistical Analysis Methods eTable 1. Search Strategy eTable 2. Inclusion and Exclusion Criteria eTable 3. Criteria for Quality of Evidence Classification eTable 4. Summary of Associations Between Glucagon-Like Peptide-1 Receptor Agonists and Several Outcomes, Including Strength of Evidence and GRADE Assessments eTable 5. AMSTAR 2 Quality Assessment by Health Outcome Domain eTable 6. Sensitivity and Leave-One-Out Analyses of Associations Between Glucagon-Like Peptide-1 Receptor Agonists and Cancer Outcomes eTable 7. Studies Excluded from the Meta-Analysis with Rationale eTable 8. Outcomes Included in the Umbrella Review eTable 9. Summary of Associations Between Glucagon-Like Peptide-1 Receptor Agonists and Selected Outcomes, Stratified by Drug Type eTable 10. Summary of Associations Between Tirzepatide and Skeletal, Respiratory, Gastrointestinal, and Cancer Outcomes eTable 11. Summary of Associations Between Glucagon-Like Peptide-1 Receptor Agonists and Selected Outcomes, Stratified by Treatment Duration and Dose eFigure 1. Overlap Among Systematic Reviews of Adverse Events, Quantified by Corrected Covered Area eFigure 2. Overlap Among Systematic Reviews of Gastrointestinal (A) and Cancer (B) Outcomes, Quantified by Corrected Covered Area eFigure 3. Overlap Among Systematic Reviews of Neurological(A), Respiratory(B), Fracture(C), and Psychiatric(D) Outcomes, Quantified by Corrected Covered Area eReferences [file jamanetwopen-e264722-s001.pdf]

Supplemental Online Content

Yang K, Liu C, Guo Q, Li Y. GLP-1 receptor agonists and noncardiometabolic outcomes: an umbrella review of meta-analyses. *JAMA Netw Open*. 2026;9(4): e264722. doi:10.1001/jamanetworkopen.2026.4722

eMethods. Description of Statistical Analysis Methods
eTable 1. Search Strategy
eTable 2. Inclusion and Exclusion Criteria
eTable 3. Criteria for Quality of Evidence Classification
eTable 4. Summary of Associations Between Glucagon-Like Peptide-1 Receptor Agonists and Several Outcomes, Including Strength of Evidence and GRADE Assessments
eTable 5. AMSTAR 2 Quality Assessment by Health Outcome Domain
eTable 6. Sensitivity and Leave-One-Out Analyses of Associations Between Glucagon-Like Peptide-1 Receptor Agonists and Cancer Outcomes
eTable 7. Studies Excluded from the Meta-Analysis with Rationale
eTable 8. Outcomes Included in the Umbrella Review
eTable 9. Summary of Associations Between Glucagon-Like Peptide-1 Receptor Agonists and Selected Outcomes, Stratified by Drug Type
eTable 10. Summary of Associations Between Tirzepatide and Skeletal, Respiratory, Gastrointestinal, and Cancer Outcomes
eTable 11. Summary of Associations Between Glucagon-Like Peptide-1 Receptor Agonists and Selected Outcomes, Stratified by Treatment Duration and Dose
eFigure 1. Overlap Among Systematic Reviews of Adverse Events, Quantified by Corrected Covered Area
eFigure 2. Overlap Among Systematic Reviews of Gastrointestinal (A) and Cancer (B) Outcomes, Quantified by Corrected Covered Area
eFigure 3. Overlap Among Systematic Reviews of Neurological(A), Respiratory(B), Fracture(C), and Psychiatric(D) Outcomes, Quantified by Corrected Covered Area
eReferences

This supplemental material has been provided by the authors to give readers additional information about their work.

© 2026 Yang K et al. *JAMA Network Open*

eMethods. Description of Statistical Analyses Methods.

**I^2 statistic**	We quantified between-study heterogeneity using the I^2 statistic, which estimates the proportion of total variation in pooled effect sizes attributable to genuine heterogeneity rather than chance. This measure also reflects the extent of overlap among the 95% confidence intervals of the included studies. Following conventional thresholds, we interpreted an I^2 value around 50% as indicative of moderate heterogeneity and values ≥75% as substantial heterogeneity.[1]
**Prediction intervals**	While 95% confidence intervals represent the precision around an estimated overall effect, 95% prediction intervals provide the range within which the effect size of a future study is likely to fall.31 Within the context of an umbrella review, prediction intervals that exclude the null suggest a statistically robust distribution of effect estimates. These intervals, along with assessments of small-study effects and excess significance bias (described below), were calculated only for meta-analyses comprising at least three primary studies.[2]
**Excess significance bias**	We evaluated excess significance bias by comparing the observed number of studies reporting nominally significant findings ($P<0.05$) with the number expected based on their estimated statistical power.[3]
**Small study effects**	Potential small-study effects were examined using Harbord's regression asymmetry test, which identifies systematic differences—often inflated effect estimates—between smaller and larger studies.[3]

© 2026 Yang K et al. *JAMA Network Open*

eTable 1. Search Strategy.

Database	Search term	Results
PubMed	("Glucagon-Like Peptide-1 Receptor Agonists"[Mesh] OR "glucagon like peptide 1 receptor agonist*"[tiab] OR "glucagon-like peptide-1 receptor agonist*"[tiab] OR "glucagon like peptide-1 receptor agonist*"[tiab] OR "glp 1 receptor agonist*"[tiab] OR "glp-1 receptor agonist*"[tiab] OR "glp 1r agonist*"[tiab] OR "glp-1r agonist*"[tiab] OR GLP1RA*[tiab] OR GLP1-RA*[tiab] OR GLP1R*[tiab] OR GLP-1R*[tiab] OR "incretin mimetic*"[tiab] OR "glp-1 analog*"[tiab] OR "glp 1 analog*"[tiab] OR "glucagon-like peptide-1 analog*"[tiab] OR exenatide[tiab] OR liraglutide[tiab] OR semaglutide[tiab] OR dulaglutide[tiab] OR lixisenatide[tiab] OR albiglutide[tiab] OR efpeglenatide[tiab] OR taspoglutide[tiab]) AND (systematic[sb] OR "Systematic Review"[pt] OR "Meta-Analysis"[pt] OR meta-analy*[tiab] OR "systematic review"[tiab] OR "network meta-analysis"[tiab] OR "network meta-analysis"[tiab] OR "mixed treatment comparison"[tiab] OR "meta synthesis"[tiab] OR metasynthes*[tiab] OR cochrane[tiab]) AND Humans[Mesh] NOT (protocol[ti] OR "study protocol"[ti])	1400
Web of Science	TS=("glucagon-like peptide-1 receptor agonist*" OR "glucagon like peptide 1 receptor agonist*" OR "GLP-1 receptor agonist*" OR "GLP 1 receptor agonist*" OR "GLP-1R agonist*" OR "GLP 1R agonist*" OR GLP1RA* OR GLP1-RA* OR GLP1R* OR GLP-1R* OR "incretin mimetic*" OR "GLP-1 analog*" OR "GLP 1 analog*" OR "glucagon-like peptide-1 analog*" OR exenatide OR liraglutide OR semaglutide OR dulaglutide OR lixisenatide OR albiglutide OR efpeglenatide OR taspoglutide) AND TS=("systematic review" OR "meta-analysis" OR metaanaly* OR "meta analy*" OR "network meta-analysis" OR "network metaanalysis" OR "mixed treatment comparison" OR "meta-synthesis" OR "meta synthesis" OR metasynthes* OR cochrane) NOT TS=(protocol OR "study protocol")	2747
Embase	('glucagon like peptide 1 receptor agonist'/exp OR 'incretin mimetic agent'/exp OR 'exenatide'/exp OR 'liraglutide'/exp OR 'semaglutide'/exp OR 'dulaglutide'/exp OR 'lixisenatide'/exp OR 'albiglutide'/exp OR 'efpeglenatide'/exp OR 'taspoglutide'/exp OR "glucagon-like peptide-1 receptor agonist*":ti,ab,kw OR "glucagon like peptide 1 receptor agonist*":ti,ab,kw OR "glucagon like peptide-1 receptor agonist*":ti,ab,kw OR "glp 1 receptor agonist*":ti,ab,kw OR "glp-1 receptor agonist*":ti,ab,kw OR "glp 1r agonist*":ti,ab,kw OR "glp-1r agonist*":ti,ab,kw OR glp1ra*:ti,ab,kw OR glp1-ra*:ti,ab,kw OR glp1r*:ti,ab,kw OR glp-1r*:ti,ab,kw OR "incretin mimetic*":ti,ab,kw OR "glp-1 analog*":ti,ab,kw OR "glp 1 analog*":ti,ab,kw OR "glucagon-like peptide-1 analog*":ti,ab,kw) AND ('systematic review'/de OR 'meta-analysis'/de OR "systematic review":ti,ab,kw OR "meta-analysis":ti,ab,kw OR metaanaly*:ti,ab,kw OR "meta analy*":ti,ab,kw OR "network meta-analysis":ti,ab,kw OR "network metaanalysis":ti,ab,kw OR "mixed treatment comparison":ti,ab,kw OR "meta synthesis":ti,ab,kw OR "meta-synthesis":ti,ab,kw OR metasynthes*:ti,ab,kw OR cochrane:ti,ab,kw) AND 'human'/de NOT (protocol:ti OR "study protocol":ti)	4689
Cochrane Database of Systematic Review	#1 MeSH descriptor: [Glucagon-Like Peptide-1 Receptor Agonists] explode all trees<br>#2 ((glucagon-like peptide-1 NEXT receptor NEXT agonist*) OR (glucagon like peptide 1 NEXT receptor NEXT agonist*) OR (GLP-1 NEXT receptor NEXT agonist*) OR (GLP 1 NEXT receptor NEXT agonist*) OR (GLP-1R NEXT agonist*) OR (GLP 1R NEXT agonist*) OR GLP1RA* OR (incretin NEXT mimetic*):ti,ab,kw	15

© 2026 Yang K et al. *JAMA Network Open*

	#3 (exenatide OR liraglutide OR semaglutide OR dulaglutide OR lixisenatide OR albiglutide OR efpeglenatide OR taspoglutide):ti,ab,kw<br>#4 #1 OR #2 OR #3 #5 ("systematic review" OR "meta-analysis"):ti,ab,kw<br>#6 #4 AND #5	
Scopus	(TITLE-ABS-KEY ("glucagon-like peptide-1 receptor agonist*") OR TITLE-ABS-KEY ("glucagon like peptide 1 receptor agonist*") OR TITLE-ABS-KEY ("glp-1 agonist*") OR TITLE-ABS-KEY ("glp-1 receptor agonist*") OR TITLE-ABS-KEY ("incretin mimetic*") OR TITLE-ABS-KEY ("glp-1 analog*") OR TITLE-ABS-KEY (exenatide OR liraglutide OR semaglutide OR dulaglutide OR lixisenatide OR albiglutide OR tirzepatide)) AND (TITLE-ABS-KEY ("meta-analysis") OR TITLE-ABS-KEY ("meta-analyses") OR TITLE-ABS-KEY ("meta-regression") OR TITLE-ABS-KEY ("meta-synthesis") OR TITLE-ABS-KEY ("systematic review"))	3556
TOTAL		12407

© 2026 Yang K et al. *JAMA Network Open*

eTable 2. Inclusion and Exclusion Criteria.

**Inclusion Criteria** (We included studies based on the following PICOS framework)
**1. Population:** The study population comprised patients receiving treatment with GLP-1 receptor agonists (GLP-1RAs), with no restrictions on demographics or underlying comorbidities.
**2. Intervention/Exposure:** The exposure of interest was therapy with any GLP-1RA.
**3. Comparison:** Eligible comparators included placebo, no treatment, or alternative glucose-lowering medications.
**4. Outcomes:** Included studies were required to investigate at least one non-cardiometabolic outcome associated with GLP-1RA use, operationalized as health outcomes not primarily related to glycemic control, body weight, or cardiorenal endpoints. Outcomes were grouped a priori into domains: oncological; gastrointestinal; hepatobiliary; neurological; psychiatric; endocrine; musculoskeletal; and other systemic conditions.
**5. Study Design:** Eligible articles were systematic reviews with meta-analyses of randomized clinical trials (RCTs). To be included, reviews had to report extractable quantitative data (i.e., effect sizes with 95% confidence intervals) from the RCTs. If a review contained data from both RCTs and non-RCTs, the results for the RCTs had to be presented separately.
**Exclusion Criteria**
**1. Outcome Focus:** Meta-analyses focusing exclusively on the established canonical effects of GLP-1RAs, such as glycemic control, weight reduction, and primary cardiorenal outcomes.
**2. Study Type:**
1) Reviews of observational, in-vitro, or animal studies.
2) Qualitative or narrative reviews that did not perform a quantitative synthesis.
3) Case reports, case series, and letters to the editor.
4) Grey literature (e.g., conference abstracts, dissertations, non-peer-reviewed articles).
**3. Data and Publication Characteristics:**
1) Reviews that did not report sufficient quantitative data to be re-analyzed (e.g., lacking effect sizes and confidence intervals).
2) Retracted articles.
3) Duplicate publications.
4) Meta-analyses that pooled data from fewer than three primary RCTs.

© 2026 Yang K et al. *JAMA Network Open*

eTable 3. Criteria for Quality of Evidence Classification.

Category	Criteria
Convincing (class I)	Number of participants >1000
	$P<10^{-6}$
	I2<50.0%
	Largest component study reporting a nominal statistically significant result (P<0.05)
	No small-study effects
	No excess significance bias
Highly suggestive (class II)	Number of participants >1000
	$P<10^{-6}$
	Largest study with a statistically significant effect (P<0.05)
Suggestive (class III)	Number of participants >1000
	$P<10^{-3}$
Weak (class IV)	P<0.05
Nonsignificant	P>0.05

© 2026 Yang K et al. *JAMA Network Open*

eTable 4. Summary of Associations Between Glucagon-Like Peptide-1 Receptor Agonists and Several Outcomes, Including Strength of Evidence and GRADE Assessments.

Author, year	Outcomes	Intervention	Comparator	No. of RCTs	Events/N (Intervention), %	Events/N (Control), %	Effect size	P-value for effect size	I^2 (%)	95% PI	Harbord's P-value	Largest study significant (Yes/No)	Downgrade factors					GRADE	Strength of evidence
													Risk of bias [a]	Imprecision [b]	Inconsistency [c]	Indirectness [d]	Publication bias [e]		
**Fracture Outcomes**																			
Yimei et al, 2025	Fracture incidence	GLP-1RA	Placebo or active control	13	23/2755 (0.83%)	23/2509 (0.92%)	0.96 (0.54 – 1.71)	0.90	0	0.54 – 1.71	0.73	No	No downgrade	Downgrade	No downgrade	No downgrade	No downgrade	Moderate	ns (Non-significant)
Zhang et al, 2025	Fracture risk	GLP-1RA	Placebo or active control	43	125/25239 (0.50%)	137/21116 (0.65%)	0.79 (0.61 – 1.01)	0.06	0	0.61 – 1.01	0.67	No	No downgrade	Downgrade	No downgrade	No downgrade	No downgrade	Moderate	ns (Non-significant)
Cheng et al, 2019	Fracture risk	GLP-1RA	Placebo or active control	38	107/21766 (0.49%)	134/17788 (0.75%)	0.67 (0.52 – 0.87)	0.003	0	0.52 – 0.87	0.02	Yes	No downgrade	No downgrade	No downgrade	No downgrade	Downgrade	Moderate	Class IV (Weak)
Su et al, 2015	Incidence of bone fractures	GLP-1RA	Placebo or active control	16	27/5698 (0.47%)	21/4082 (0.51%)	0.96 (0.52 – 1.79)	0.91	0	0.52 – 1.79	0.75	No	No downgrade	Downgrade	No downgrade	No downgrade	No downgrade	Moderate	ns (Non-significant)
Mabilleau et al, 2014	Incidence of bone fractures	GLP-1RA	Placebo or active control	7	13/2905 (0.45%)	6/1331 (0.45%)	0.76 (0.29 – 2.02)	0.59	0	0.29 – 2.02	0.31	No	No downgrade	Downgrade	No downgrade	No downgrade	No downgrade	Moderate	ns (Non-significant)
**Respiratory Outcomes**																			
Zhang et al, 2024	Asthma	GLP-1RA	Placebo or active control	34	8038/37387 (21.50%)	4915/37620 (13.06%)	1.17 (0.92 – 1.49)	0.20	100	0.32 – 4.31	0.67	No	No downgrade	Downgrade	Downgrade	No downgrade	No downgrade	Low	ns (Non-significant)
Yu et al, 2023	Incidence of respiratory diseases	GLP-1RA	Placebo or active control	28	1431/40232 (3.56%)	1513/34309 (4.41%)	0.85 (0.80 – 0.92)	< 0.001	0	0.80 – 0.92	0.81	No	No downgrade	No downgrade	No downgrade	No downgrade	No downgrade	High	Class III (Suggestive)

© 2026 Yang K et al. *JAMA Network Open*

Wei et al, 2021	Bronchitis	GLP-1RA	Placebo	7	48/27894 (0.17%)	60/27920 (0.21%)	0.81 (0.55 – 1.19)	0.29	0	0.55 – 1.19	0.80	No	Downgrade	Downgrade	No downgrade	No downgrade	No downgrade	Low	ns (Non-significant)
Wei et al, 2021	Pneumonia	GLP-1RA	Placebo	6	423/20175 (2.10%)	477/20131 (2.37%)	0.89 (0.78 – 1.01)	0.07	0	0.78 – 1.01	0.10	No	Downgrade	Downgrade	No downgrade	No downgrade	No downgrade	Low	ns (Non-significant)
Wei et al, 2021	Upper respiratory tract infection	GLP-1RA	Placebo	6	12/20586 (0.06%)	18/20590 (0.09%)	0.72 (0.34 – 1.51)	0.38	0	0.34 – 1.51	0.47	No	Downgrade	Downgrade	No downgrade	No downgrade	No downgrade	Low	ns (Non-significant)
Wei et al, 2021	Lung adenocarcinoma	GLP-1RA	Placebo	7	17/27925 (0.06%)	19/27961 (0.07%)	0.92 (0.46 – 1.83)	0.81	0	0.46 – 1.83	0.86	No	Downgrade	Downgrade	No downgrade	No downgrade	No downgrade	Low	ns (Non-significant)
Wei et al, 2021	Squamous cell carcinoma of lung	GLP-1RA	Placebo	7	9/27933 (0.03%)	19/27961 (0.07%)	0.57 (0.26 – 1.26)	0.17	0	0.26 – 1.26	0.81	No	Downgrade	Downgrade	No downgrade	No downgrade	No downgrade	Low	ns (Non-significant)
Wei et al, 2021	Acute respiratory failure	GLP-1RA	Placebo	7	53/27889 (0.19%)	70/27910 (0.25%)	0.76 (0.48 – 1.18)	0.22	25.1	0.36 – 1.58	0.42	No	Downgrade	Downgrade	No downgrade	No downgrade	No downgrade	Low	ns (Non-significant)
Wei et al, 2021	Asthma	GLP-1RA	Placebo	7	30/27882 (0.11%)	38/27904 (0.14%)	0.83 (0.51 – 1.34)	0.45	0	0.51 – 1.34	0.13	No	Downgrade	Downgrade	No downgrade	No downgrade	No downgrade	Low	ns (Non-significant)
Wei et al, 2021	Chronic obstructive pulmonary disease	GLP-1RA	Placebo	7	175/27592 (0.63%)	196/27588 (0.71%)	0.89 (0.73 – 1.09)	0.27	0	0.73 – 1.09	0.55	No	Downgrade	Downgrade	No downgrade	No downgrade	No downgrade	Low	ns (Non-significant)
Wei et al, 2021	Interstitial lung disease	GLP-1RA	Placebo	4	19/19948 (0.10%)	10/20005 (0.05%)	1.89 (0.88 – 4.09)	0.11	0	0.88 – 4.09	0.94	No	Downgrade	Downgrade	No downgrade	No downgrade	No downgrade	Low	ns (Non-significant)
Wei et al, 2021	Pulmonary fibrosis	GLP-1RA	Placebo	6	4/24903 (0.02%)	10/24928 (0.04%)	0.47 (0.15 – 1.46)	0.19	0	0.15 – 1.46	0.11	No	Downgrade	Downgrade	No downgrade	No downgrade	No downgrade	Low	ns (Non-significant)

© 2026 Yang K et al. *JAMA Network Open*

Wei et al, 2021	Pulmonary edema	GLP-1RA	Placebo	6	35/24841 (0.14%)	56/24836 (0.23%)	0.62 (0.37 – 1.04)	0.07	15.3	0.31 – 1.27	0.48	No	Downgrade	Downgrade	No downgrade	No downgrade	No downgrade	Low	ns (Non-significant)
Wei et al, 2021	Sleep apnea syndrome	GLP-1RA	Placebo	6	20/26254 (0.08%)	23/26285 (0.09%)	1.00 (0.53 – 1.89)	1.00	0	0.53 – 1.89	0.31	No	Downgrade	Downgrade	No downgrade	No downgrade	No downgrade	Low	ns (Non-significant)
**Neurological Outcomes**																			
Seminer et al, 2025	All-cause dementia	GLP-1RA	Placebo	10	28/39745 (0.07%)	53/38183 (0.14%)	0.55 (0.35 – 0.87)	0.01	0	0.35 – 0.87	0.45	No	No downgrade	No downgrade	No downgrade	Downgrade	No downgrade	Moderate	Class IV (Weak)
Seminer et al, 2025	Vascular dementia	GLP-1RA	Placebo	5	5/27357 (0.02%)	10/27393 (0.04%)	0.56 (0.19 – 1.70)	0.31	0	0.19 – 1.70	0.84	No	No downgrade	Downgrade	No downgrade	Downgrade	No downgrade	Low	ns (Non-significant)
Seminer et al, 2025	Alzheimer dementia	GLP-1RA	Placebo	5	8/30491 (0.03%)	3/30547 (0.01%)	1.60 (0.44 – 5.78)	0.47	0	0.44 – 5.78	0.13	No	No downgrade	Downgrade	No downgrade	Downgrade	No downgrade	Low	ns (Non-significant)
Sindhu et al, 2024	Seizure and epilepsy risk	GLP-1RA	Placebo	8	45/30570 (0.15%)	65/29205 (0.22%)	0.73 (0.49 – 1.07)	0.11	0	0.49 – 1.07	0.05	No	No downgrade	Downgrade	No downgrade	Downgrade	No downgrade	Low	ns (Non-significant)
Sindhu et al, 2024	Seizure risk	GLP-1RA	Placebo	8	30/30600 (0.10%)	48/29239 (0.16%)	0.64 (0.40 – 1.03)	0.07	0	0.40 – 1.03	0.14	No	No downgrade	Downgrade	No downgrade	Downgrade	No downgrade	Low	ns (Non-significant)
Sindhu et al, 2024	Epilepsy risk	GLP-1RA	Placebo	8	15/30630 (0.05%)	17/29301 (0.06%)	0.95 (0.39 – 2.34)	0.91	22.2	0.22 – 4.18	0.57	No	No downgrade	Downgrade	No downgrade	Downgrade	No downgrade	Low	ns (Non-significant)
Tang et al, 2023	Parkinson disease	GLP-1RA	Placebo	8	2/30676 (0.01%)	4/29361 (0.01%)	0.68 (0.21 – 2.18)	0.52	0	0.21 – 2.18	0.01	No	Undetermined	Downgrade	No downgrade	Downgrade	Downgrade	Very low	ns (Non-significant)
**Psychiatric Outcomes**																			
Chen et al, 2025	Suicidal behavior	GLP-1RA	Placebo or active control	25	35/42172 (0.08%)	36/39223 (0.09%)	0.85 (0.54	0.47	0	0.54 – 1.33	0.12	No	Undetermined	Downgrade	No downgrade	Downgrade	No downgrade	Low	ns (Non-significant)

© 2026 Yang K et al. *JAMA Network Open*

							– 1.33)												
Ebrahimi et al, 2025	Suicide and/or self-harm adverse events	GLP-1RA	Placebo	27	33/74740 (0.04%)	27/68095 (0.04%)	0.75 (0.47 – 1.19)	0.22	0	0.47 – 1.19	0.24	No	No downgrade	Downgrade	No downgrade	Downgrade	No downgrade	Low	ns (Non-significant)
Silverii et al, 2024	Psychiatric disorder	GLP-1RA	Placebo or active control	31	318/44415 (0.72%)	281/40144 (0.70%)	0.96 (0.81 – 1.14)	0.65	0	0.81 – 1.14	0.73	No	Undetermined	Downgrade	No downgrade	Downgrade	No downgrade	Low	ns (Non-significant)
Silverii et al, 2024	Suicidal behavior	GLP-1RA	Placebo or active control	11	18/25030 (0.07%)	19/23408 (0.08%)	0.88 (0.47 – 1.67)	0.70	0	0.47 – 1.67	0.66	No	Undetermined	Downgrade	No downgrade	Downgrade	No downgrade	Low	ns (Non-significant)
Silverii GA al, 2024	Depression	GLP-1RA	Placebo or active control	17	80/30828 (0.26%)	58/27791 (0.21%)	0.95 (0.66 – 1.36)	0.79	1.5	0.63 – 1.42	0.40	No	Undetermined	Downgrade	No downgrade	Downgrade	No downgrade	Low	ns (Non-significant)
Silverii GA al, 2024	Anxiety	GLP-1RA	Placebo or active control	13	23/27494 (0.08%)	24/24997 (0.10%)	0.84 (0.48 – 1.48)	0.56	0	0.48 – 1.48	0.59	No	Undetermined	Downgrade	No downgrade	Downgrade	No downgrade	Low	ns (Non-significant)
Endocrine Outcomes																			
Hu et al, 2022	Overall thyroid disorders	GLP-1RA	Placebo or active control	45	204/52396 (0.39%)	129/41334 (0.31%)	1.27 (1.02 – 1.59)	0.03	0	1.02 – 1.59	0.04	No	No downgrade	No downgrade	No downgrade	No downgrade	Downgrade	Moderate	Class IV (Weak)
Hu et al, 2022	Hyperthyroidism	GLP-1RA	Placebo or active control	9	19/25715 (0.07%)	14/24522 (0.06%)	1.20 (0.60 – 2.38)	0.61	0	0.60 – 2.38	0.20	No	No downgrade	Downgrade	No downgrade	No downgrade	No downgrade	Moderate	ns (Non-significant)
Hu et al, 2022	Hypothyroidism	GLP-1RA	Placebo or active control	6	47/13792 (0.34%)	36/12854 (0.28%)	1.23 (0.80 – 1.89)	0.34	0	0.80 – 1.89	0.05	No	No downgrade	Downgrade	No downgrade	No downgrade	No downgrade	Moderate	ns (Non-significant)
Hu et al, 2022	Thyroiditis	GLP-1RA	Placebo or active control	6	8/20931 (0.04%)	1/18919 (0.01%)	1.56 (0.41 – 5.93)	0.51	0	0.41 – 5.93	0.001	No	No downgrade	Downgrade	No downgrade	No downgrade	Downgrade	Low	ns (Non-significant)

© 2026 Yang K et al. *JAMA Network Open*

Hu et al, 2022	Goiter	GLP-1RA	Placebo or active control	18	43/37370 (0.12%)	31/31920 (0.10%)	1.14 (0.71 – 1.81)	0.59	0	0.71 – 1.81	0.02	No	No downgrade	Downgrade	No downgrade	No downgrade	Downgrade	Low	ns (Non-significant)
**Hepatic Outcomes**																			
Mantovani et al, 2025	Nonalcoholic steatohepatitis resolution	GLP-1RA	Placebo or active control	6	438/566 (77.39%)	338/696 (48.56%)	3.39 (2.63 – 4.36)	< 0.001	0	2.63 – 4.36	0.70	Yes	No downgrade	No downgrade	No downgrade	No downgrade	No downgrade	High	Class I (Convincing)
Mantovani et al, 2025	Fibrosis stage	GLP-1RA	Placebo or active control	5	275/395 (69.62%)	454/796 (57.04%)	1.79 (1.37 – 2.35)	< 0.001	1.1	1.36 – 2.37	0.49	Yes	No downgrade	No downgrade	No downgrade	No downgrade	No downgrade	High	Class III (Suggestive)
**Gastrointestinal Outcomes**																			
Chiang et al, 2025	Cholecystitis	GLP-1RA	Placebo	40	256/41272 (0.62%)	176/33809 (0.52%)	1.17 (0.91 – 1.50)	0.23	9.3	0.71 – 1.91	0.45	No	No downgrade	Downgrade	No downgrade	No downgrade	No downgrade	Moderate	ns (Non-significant)
Chiang et al, 2025	Cholelithiasis	GLP-1RA	Placebo	37	321/44436 (0.72%)	194/36852 (0.53%)	1.32 (1.07 – 1.62)	0.008	4.7	0.95 – 1.83	0.28	No	No downgrade	No downgrade	No downgrade	No downgrade	No downgrade	High	Class IV (Weak)
Chiang et al, 2025	Cholangitis	GLP-1RA	Placebo	9	31/28458 (0.11%)	17/27134 (0.06%)	1.49 (0.83 – 2.67)	0.19	0	0.83 – 2.67	0.59	No	No downgrade	Downgrade	No downgrade	No downgrade	No downgrade	Moderate	ns (Non-significant)
Chiang et al, 2025	Cholestasis	GLP-1RA	Placebo	11	17/35353 (0.05%)	19/33514 (0.06%)	0.80 (0.41 – 1.56)	0.52	0	0.41 – 1.56	0.86	No	No downgrade	Downgrade	No downgrade	No downgrade	No downgrade	Moderate	ns (Non-significant)
Chiang et al, 2025	Pancreatitis	GLP-1RA	Placebo	26	167/49508 (0.34%)	149/43988 (0.34%)	1.01 (0.78 – 1.29)	0.96	6.7	0.67 – 1.52	0.05	No	No downgrade	Downgrade	No downgrade	No downgrade	Downgrade	Low	ns (Non-significant)
Chiang et al, 2025	Gastroesophageal reflux disease	GLP-1RA	Placebo	33	471/44299 (1.06%)	125/39564 (0.32%)	2.19 (1.65 – 2.90)	< *0.001*	27.2	0.99 – 4.82	0.85	No	No downgrade	No downgrade	No downgrade	No downgrade	No downgrade	High	Class III (Suggestive)

© 2026 Yang K et al. *JAMA Network Open*

Chiang et al, 2025	Gastritis	GLP-1RA	Placebo	23	108/40687 (0.27%)	98/37195 (0.26%)	0.95 (0.72 – 1.25)	0.71	0	0.72 – 1.25	0.43	No	No downgrade	Downgrade	No downgrade	No downgrade	No downgrade	Moderate	ns (Non-significant)
Chiang et al, 2025	Esophagitis	GLP-1RA	Placebo	12	21/35555 (0.06%)	12/33417 (0.04%)	1.17 (0.58 – 2.33)	0.66	0	0.58 – 2.33	0.19	No	No downgrade	Downgrade	No downgrade	No downgrade	No downgrade	Moderate	ns (Non-significant)
Chiang et al, 2025	Gastrointestinal ischemia	GLP-1RA	Placebo	17	34/39056 (0.09%)	20/36775 (0.05%)	1.29 (0.76 – 2.19)	0.34	0	0.76 – 2.19	0.40	No	No downgrade	Downgrade	No downgrade	No downgrade	No downgrade	Moderate	ns (Non-significant)
Chiang et al, 2025	Gastrointestinal hemorrhage	GLP-1RA	Placebo	25	280/40659 (0.69%)	273/37405 (0.73%)	0.99 (0.84 – 1.17)	0.92	0	0.84 – 1.17	0.01	No	No downgrade	Downgrade	No downgrade	No downgrade	Downgrade	Low	ns (Non-significant)
Chiang et al, 2025	Intestinal obstruction	GLP-1RA	Placebo	19	80/39750 (0.20%)	87/36135 (0.24%)	0.90 (0.65 – 1.23)	0.50	2.5	0.61 – 1.33	0.08	No	No downgrade	Downgrade	No downgrade	No downgrade	No downgrade	Moderate	ns (Non-significant)
Chiang et al, 2025	Paralytic ileus	GLP-1RA	Placebo	8	18/34082 (0.05%)	20/32864 (0.06%)	0.79 (0.41 – 1.53)	0.49	0	0.41 – 1.53	0.15	No	No downgrade	Downgrade	No downgrade	No downgrade	No downgrade	Moderate	ns (Non-significant)
Chiang et al, 2025	Gastrointestinal ulceration	GLP-1RA	Placebo	19	62/40571 (0.15%)	70/36738 (0.19%)	0.81 (0.58 – 1.14)	0.23	0	0.58 – 1.14	0.18	No	No downgrade	Downgrade	No downgrade	No downgrade	No downgrade	Moderate	ns (Non-significant)
Chiang et al, 2025	Gastrointestinal perforation	GLP-1RA	Placebo	11	19/36777 (0.05%)	26/34514 (0.08%)	0.74 (0.40 – 1.35)	0.33	0	0.40 – 1.35	0.50	No	No downgrade	Downgrade	No downgrade	No downgrade	No downgrade	Moderate	ns (Non-significant)
Chiang et al, 2025	Gastroparesis	GLP-1RA	Placebo	10	12/25860 (0.05%)	6/25526 (0.02%)	1.33 (0.52 – 3.41)	0.55	0	0.52 – 3.41	0.02	No	No downgrade	Downgrade	No downgrade	No downgrade	Downgrade	Low	ns (Non-significant)
Safwan et al, 2025	Cholelithiasis	GLP-1RA	Placebo	7	77/4508 (1.71%)	22/2364 (0.93%)	1.91 (1.18 – 3.10)	0.01	0	1.18 – 3.10	0.73	No	Undetermined	No downgrade	No downgrade	No downgrade	No downgrade	High	Class IV (Weak)

© 2026 Yang K et al. *JAMA Network Open*

Safwan et al, 2025	Pancreatitis	GLP-1RA	Placebo	10	27/14564 (0.19%)	27/11569 (0.23%)	0.76 (0.45–1.29)	0.30	0	0.45–1.29	0.36	No	Undetermined	Downgrade	No downgrade	No downgrade	No downgrade	Moderate	ns (Non-significant)
Wen et al, 2025	Pancreatitis	GLP-1RA	Placebo or active control	62	133/36813 (0.36%)	77/29759 (0.26%)	1.21 (0.93–1.56)	0.16	0	0.93–1.56	0.19	No	No downgrade	Downgrade	No downgrade	No downgrade	No downgrade	Moderate	ns (Non-significant)
Masson et al, 2024	Acute pancreatitis	Semaglutide	Placebo	22	35/19033 (0.18%)	42/15759 (0.27%)	0.70 (0.46–1.06)	0.10	0	0.46–1.06	0.50	No	Downgrade	Downgrade	No downgrade	No downgrade	No downgrade	Low	ns (Non-significant)
Muhammed et al, 2024	Pancreatitis	GLP-1RA	Placebo or active control	37	54/24732 (0.22%)	42/22492 (0.19%)	1.03 (0.72–1.48)	0.88	0	0.72–1.48	0.07	No	Downgrade	Downgrade	No downgrade	No downgrade	No downgrade	Low	ns (Non-significant)
Muhammed et al, 2024	Chronic pancreatitis	GLP-1RA	Placebo or active control	4	4/6020 (0.07%)	3/5238 (0.06%)	1.01 (0.27–3.84)	0.99	0	0.27–3.84	0.03	No	Downgrade	Downgrade	No downgrade	No downgrade	Downgrade	Very low	ns (Non-significant)
Wang et al, 2024	Acute cholecystitis	GLP-1RA	Placebo or active control	11	108/29005 (0.37%)	62/25078 (0.25%)	1.50 (1.08–2.08)	0.02	0	1.08–2.08	0.52	No	Downgrade	No downgrade	No downgrade	No downgrade	No downgrade	Moderate	Class IV (Weak)
Wang et al, 2024	Gastric ulcer	GLP-1RA	Placebo or active control	10	21/33050 (0.06%)	21/30801 (0.07%)	0.90 (0.49–1.64)	0.73	0	0.49–1.64	0.64	No	Downgrade	Downgrade	No downgrade	No downgrade	No downgrade	Low	ns (Non-significant)
Wang et al, 2024	Gastric ulcer hemorrhage	GLP-1RA	Placebo or active control	8	24/31550 (0.08%)	6/28986 (0.02%)	2.27 (0.96–5.34)	0.06	0	0.96–5.34	0.92	No	Downgrade	Downgrade	No downgrade	No downgrade	No downgrade	Low	ns (Non-significant)
Wang et al, 2024	Pancreatitis	GLP-1RA	Placebo or active control	10	77/32236 (0.24%)	48/30629 (0.16%)	1.52 (1.06–2.20)	0.02	0	1.06–2.20	0.38	No	Downgrade	No downgrade	No downgrade	No downgrade	No downgrade	Moderate	Class IV (Weak)
He et al, 2022	Cholelithiasis	GLP-1RA	Placebo or active control	62	454/53654 (0.85%)	287/42212 (0.68%)	1.22 (1.05–1.42)	0.01	0	1.05–1.42	0.58	No	Downgrade	No downgrade	No downgrade	No downgrade	No downgrade	Moderate	Class IV (Weak)

© 2026 Yang K et al. *JAMA Network Open*

He et al, 2022	Cholecystitis	GLP-1RA	Placebo or active control	53	302/49491 (0.61%)	187/40574 (0.46%)	1.33 (1.10 – 1.60)	0.003	0	1.10 – 1.60	0.08	No	Downgrade	No downgrade	No downgrade	No downgrade	No downgrade	Moderate	Class IV (Weak)
He et al, 2022	Biliary diseases	GLP-1RA	Placebo or active control	21	79/36225 (0.22%)	40/32741 (0.12%)	1.51 (1.04 – 2.19)	0.03	0	1.04 – 2.19	0.37	No	Downgrade	No downgrade	No downgrade	No downgrade	No downgrade	Moderate	Class IV (Weak)
He et al, 2022	Gallbladder or biliary diseases	GLP-1RA	Placebo or active control	77	911/57856 (1.57%)	538/45657 (1.18%)	1.34 (1.16 – 1.55)	*< 0.001*	8.4	0.95 – 1.89	0.68	No	Downgrade	No downgrade	No downgrade	No downgrade	No downgrade	Moderate	Class III (Suggestive)
Nreu et al, 2020	Cholelithiasis	GLP-1RA	Placebo or active control	43	454/38953 (1.17%)	348/35893 (0.97%)	1.18 (0.94 – 1.48)	0.16	13.7	0.73 – 1.90	0.14	No	No downgrade	Downgrade	No downgrade	No downgrade	No downgrade	Moderate	ns (Non-significant)
Nreu et al, 2020	Pancreatitis	GLP-1RA	Placebo or active control	43	123/38953 (0.32%)	111/35893 (0.31%)	1.13 (0.86 – 1.47)	0.37	0	0.86 – 1.47	0.89	No	No downgrade	Downgrade	No downgrade	No downgrade	No downgrade	Moderate	ns (Non-significant)
Monami et al, 2017	Cholelithiasis	GLP-1RA	Placebo or any other non-GLP-1RAs drug	26	141/17232 (0.82%)	99/14872 (0.67%)	1.29 (1.00 – 1.67)	0.05	0	1.00 – 1.67	0.07	No	Downgrade	Downgrade	No downgrade	No downgrade	No downgrade	Low	ns (Non-significant)
Monami et al, 2014	Pancreatitis	GLP-1RA	Placebo or active control	9	10/3214 (0.31%)	6/2137 (0.28%)	0.91 (0.32 – 2.56)	0.85	0	0.32 – 2.56	0.96	No	Downgrade	Downgrade	No downgrade	No downgrade	No downgrade	Low	ns (Non-significant)
Alves et al, 2012	Acute pancreatitis	GLP-1RA	Placebo or active control	25	87/63080 (0.14%)	1094/713180 (0.15%)	0.81 (0.57 – 1.16)	0.25	11.8	0.44 – 1.51	0.45	No	Downgrade	Downgrade	No downgrade	No downgrade	No downgrade	Low	ns (Non-significant)
**Cancer Outcomes**																			
Duchemin et al, 2025	Thyroid cancer	GLP-1RA	Placebo	44	70/47588 (0.15%)	44/36568 (0.12%)	1.03 (0.69 – 1.53)	0.89	0.9	0.64 – 1.65	0.06	No	Downgrade	Downgrade	Downgrade	No downgrade	Downgrade	Very low	Class III (Suggestive)

© 2026 Yang K et al. *JAMA Network Open*

Ko et al, 2025	Colorectal cancer	GLP-1RA	Placebo	22	196/41451 (0.47%)	155/37685 (0.41%)	1.20 (0.97 – 1.48)	0.10	0	0.97 – 1.48	0.09	No	No downgrade	Downgrade	No downgrade	No downgrade	No downgrade	Moderate	ns (Non-significant)
Ko et al, 2025	Thyroid cancer	GLP-1RA	Placebo	49	87/101749 (0.09%)	40/93330 (0.04%)	1.25 (0.88 – 1.77)	0.22	0	0.88 – 1.77	0.04	No	No downgrade	Downgrade	No downgrade	No downgrade	Downgrade	Low	ns (Non-significant)
Ko et al, 2025	Esophageal cancer	GLP-1RA	Placebo	5	23/25885 (0.09%)	29/25840 (0.11%)	0.80 (0.46 – 1.39)	0.43	0	0.46 – 1.39	0.07	No	No downgrade	Downgrade	No downgrade	No downgrade	No downgrade	Moderate	ns (Non-significant)
Ko et al, 2025	Endometrial cancer	GLP-1RA	Placebo	18	35/14804 (0.24%)	35/13185 (0.27%)	0.85 (0.52 – 1.40)	0.53	1.9	0.48 – 1.52	0.28	No	No downgrade	Downgrade	No downgrade	No downgrade	No downgrade	Moderate	ns (Non-significant)
Ko et al, 2025	Liver cancer	GLP-1RA	Placebo	11	43/35763 (0.12%)	50/34681 (0.14%)	0.86 (0.52 – 1.42)	0.55	18.2	0.37 – 2.01	0.71	No	No downgrade	Downgrade	No downgrade	No downgrade	No downgrade	Moderate	ns (Non-significant)
Ko et al, 2025	Kidney cancer	GLP-1RA	Placebo	18	91/38155 (0.24%)	76/35962 (0.21%)	1.09 (0.80 – 1.47)	0.58	0	0.80 – 1.47	0.93	No	No downgrade	Downgrade	No downgrade	No downgrade	No downgrade	Moderate	ns (Non-significant)
Ko et al, 2025	Pancreatic cancer	GLP-1RA	Placebo	35	136/81366 (0.17%)	134/73644 (0.18%)	0.93 (0.73 – 1.19)	0.59	0	0.73 – 1.19	0.001	No	No downgrade	Downgrade	No downgrade	No downgrade	Downgrade	Low	ns (Non-significant)
Ko et al, 2025	Gallbladder cancer	GLP-1RA	Placebo	14	23/39134 (0.06%)	16/36078 (0.04%)	1.14 (0.60 – 2.15)	0.69	0	0.60 – 2.15	0.02	No	No downgrade	Downgrade	No downgrade	No downgrade	Downgrade	Low	ns (Non-significant)
Ko et al, 2025	Ovarian cancer	GLP-1RA	Placebo	12	19/13235 (0.14%)	11/12177 (0.09%)	1.11 (0.53 – 2.32)	0.79	0	0.53 – 2.32	0.67	No	No downgrade	Downgrade	No downgrade	No downgrade	No downgrade	Moderate	ns (Non-significant)
Ko et al, 2025	Breast cancer	GLP-1RA	Placebo	28	116/45638 (0.25%)	97/39690 (0.24%)	1.04 (0.79 – 1.36)	0.80	0	0.79 – 1.36	0.09	No	No downgrade	Downgrade	No downgrade	No downgrade	No downgrade	Moderate	ns (Non-significant)

© 2026 Yang K et al. *JAMA Network Open*

Ko et al, 2025	Gastric cancer	GLP-1RA	Placebo	12	53/35890 (0.15%)	45/34838 (0.13%)	1.04 (0.69 – 1.57)	0.84	0	0.69 – 1.57	0.87	No	No downgrade	Downgrade	No downgrade	No downgrade	No downgrade	Moderate	ns (Non-significant)
Ko et al, 2025	Multiple myeloma	GLP-1RA	Placebo	10	27/35887 (0.08%)	26/34672 (0.07%)	1.03 (0.60 – 1.75)	0.92	0	0.60 – 1.75	0.41	No	No downgrade	Downgrade	No downgrade	No downgrade	No downgrade	Moderate	ns (Non-significant)
Ko et al, 2025	Meningioma	GLP-1RA	Placebo	7	11/32441 (0.03%)	9/31226 (0.03%)	1.04 (0.43 – 2.53)	0.93	0	0.43 – 2.53	0.97	No	No downgrade	Downgrade	No downgrade	No downgrade	No downgrade	Moderate	ns (Non-significant)
Rao et al, 2025	Bladder cancer	GLP-1RA	Placebo	11	25/31464 (0.08%)	35/29874 (0.12%)	0.74 (0.43 – 1.26)	0.26	0	0.43 – 1.26	0.19	No	No downgrade	Downgrade	No downgrade	No downgrade	No downgrade	Moderate	ns (Non-significant)
Silverii et al, 2025	Colorectal cancer	GLP-1RA	Placebo	24	199/43557 (0.46%)	144/38844 (0.37%)	1.24 (1.00 – 1.54)	0.05	0	1.00 – 1.54	0.85	No	Downgrade	No downgrade	No downgrade	No downgrade	No downgrade	Moderate	Class IV (Weak)
Silverii et al, 2025	Thyroid cancer	GLP-1RA	Placebo	28	77/46412 (0.17%)	31/41053 (0.08%)	1.43 (0.95 – 2.13)	0.08	0	0.95 – 2.13	0.90	No	Downgrade	Downgrade	No downgrade	No downgrade	No downgrade	Low	ns (Non-significant)
Silverii et al, 2025	Endometrial cancer	GLP-1RA	Placebo	7	15/24348 (0.06%)	21/23602 (0.09%)	0.68 (0.35 – 1.31)	0.25	0	0.35 – 1.31	0.23	No	Downgrade	Downgrade	No downgrade	No downgrade	No downgrade	Low	ns (Non-significant)
Silverii et al, 2025	Overall cancer	GLP-1RA	Placebo	50	1725/55305 (3.12%)	1568/47467 (3.30%)	1.03 (0.97 – 1.10)	0.34	0	0.97 – 1.10	0.15	No	Downgrade	Downgrade	No downgrade	No downgrade	No downgrade	Low	ns (Non-significant)
Silverii et al, 2025	Uterine cancer	GLP-1RA	Placebo	15	34/35940 (0.09%)	40/34435 (0.12%)	0.77 (0.44 – 1.37)	0.38	11.2	0.31 – 1.93	0.03	No	Downgrade	Downgrade	No downgrade	No downgrade	Downgrade	Very low	ns (Non-significant)
Wen et al, 2025	Pancreatic cancer	GLP-1RA	Placebo or active control	62	36/36813 (0.10%)	37/29759 (0.12%)	0.91 (0.61 – 1.36)	0.65	2.5	0.48 – 1.72	0.00	Yes	No downgrade	Downgrade	No downgrade	No downgrade	No downgrade	Moderate	ns (Non-significant)

© 2026 Yang K et al. *JAMA Network Open*

Wu et al, 2025	Esophageal cancer	GLP-1RA	Placebo	5	2/6507 (0.03%)	5/11933 (0.04%)	0.87 (0.20 – 3.82)	0.86	0	0.20 – 3.82	0.23	No	No downgrade	Downgrade	No downgrade	No downgrade	No downgrade	Moderate	ns (Non-significant)
Figlioli et al, 2024	Gastrointestinal cancer	GLP-1RA	Placebo or any other non-GLP-1RAs antidiabetic drug	90	436/70646 (0.62%)	380/54145 (0.70%)	0.97 (0.85 – 1.12)	0.68	0	0.85 – 1.12	0.11	No	Downgrade	Downgrade	No downgrade	No downgrade	No downgrade	Low	ns (Non-significant)
Muhammed et al, 2024	Pancreatic cancer	Anti-diabetic medications (alone or in combination)	Placebo or active control	10	51/22250 (0.23%)	36/21158 (0.17%)	1.17 (0.65 – 2.12)	0.60	22.4	0.42 – 3.26	0.18	No	No downgrade	Downgrade	No downgrade	Downgrade	No downgrade	Low	ns (Non-significant)
Silverii et al, 2024	Overall thyroid cancer	GLP-1RA	Placebo or active control	26	73/36886 (0.20%)	27/32591 (0.08%)	1.64 (1.08 – 2.50)	0.02	0	1.08 – 2.50	0.50	No	Downgrade	No downgrade	No downgrade	No downgrade	No downgrade	Moderate	Class IV (Weak)
Nagendra et al, 2023	Pancreatic cancer	Semaglutide	Placebo control group (PCG) and active control group (ACG)	50	6/25910 (0.02%)	14/17962 (0.08%)	0.51 (0.30 – 0.85)	0.01	0	0.30 – 0.85	0.00	No	Downgrade	No downgrade	No downgrade	No downgrade	Downgrade	Low	Class IV (Weak)
Nagendra et al, 2023	Thyroid cancer	Semaglutide	Placebo control group (PCG) and active control	52	12/27204 (0.04%)	2/22782 (0.01%)	0.78 (0.47 – 1.31)	0.35	0	0.47 – 1.31	0.004	No	Downgrade	Downgrade	No downgrade	No downgrade	Downgrade	Very low	ns (Non-significant)

© 2026 Yang K et al. *JAMA Network Open*

			group (ACG)															
Nagendra et al, 2023	Neoplasms	Semaglutide	Placebo control group (PCG) and active control group (ACG)	80	210/39822 (0.53%)	76/26974 (0.28%)	0.96 (0.75 – 1.23)	0.76	0	0.75 – 1.23	0.29	No	Downgrade	Downgrade	No downgrade	No downgrade	No downgrade	Low	ns (Non-significant)
He et al, 2022	Biliary cancer	GLP-1RA	Placebo or active control	12	24/31010 (0.08%)	15/30026 (0.05%)	1.45 (0.77 – 2.76)	0.25	0	0.77 – 2.76	0.02	No	Downgrade	Downgrade	No downgrade	No downgrade	Downgrade	Very low	ns (Non-significant)
Hu et al, 2022	Thyroid cancer	GLP-1RA	Placebo or active control	35	63/43061 (0.15%)	29/34330 (0.08%)	1.26 (0.82 – 1.92)	0.29	0	0.82 – 1.92	0.01	No	No downgrade	Downgrade	No downgrade	No downgrade	Downgrade	Low	ns (Non-significant)
Piccoli et al, 2021	Breast cancer	GLP-1RA	Placebo or active control	51	129/48267 (0.27%)	107/40755 (0.26%)	0.95 (0.74 – 1.23)	0.71	0	0.74 – 1.23	0.24	No	No downgrade	Downgrade	No downgrade	No downgrade	No downgrade	Moderate	ns (Non-significant)
Cao et al, 2020	Pancreatic cancer	GLP-1RA	Placebo	6	57/26357 (0.22%)	51/26392 (0.19%)	1.08 (0.63 – 1.86)	0.77	38.4	0.41 – 2.84	0.30	No	No downgrade	Downgrade	No downgrade	No downgrade	No downgrade	Moderate	ns (Non-significant)
Cao et al, 2019	Thyroid cancer	GLP-1RA	Placebo or active control	15	34/22276 (0.15%)	17/20525 (0.08%)	1.39 (0.77 – 2.50)	0.27	0	0.77 – 2.50	0.05	No	No downgrade	Downgrade	No downgrade	No downgrade	Downgrade	Low	ns (Non-significant)
Cao et al, 2019	Overall cancer	GLP-1RA	Placebo or active control	36	1342/34102 (3.94%)	1223/29423 (4.16%)	1.02 (0.92 – 1.13)	0.72	8.2	0.85 – 1.23	0.66	No	No downgrade	Downgrade	No downgrade	No downgrade	No downgrade	Moderate	ns (Non-significant)
Cao et al, 2019	Pancreatic cancer	GLP-1RA	Placebo or active control	16	48/25102 (0.19%)	41/23684 (0.17%)	0.97 (0.60 – 1.58)	0.90	8.4	0.46 – 2.04	0.65	No	No downgrade	Downgrade	No downgrade	No downgrade	No downgrade	Moderate	ns (Non-significant)

© 2026 Yang K et al. *JAMA Network Open*

Liu et al, 2019	Neoplasms	GLP-1RA	Placebo or other interventions	26	773/24702 (3.13%)	723/21525 (3.36%)	1.04 (0.94 – 1.15)	0.47	0	0.94 – 1.15	0.75	No	No downgrade	Downgrade	No downgrade	No downgrade	No downgrade	Moderate	ns (Non-significant)
Pinto et al, 2019	Pancreatic cancer	GLP-1RA	Placebo or other antidiabetic interventions	12	37/18394 (0.20%)	33/18000 (0.18%)	0.98 (0.52 – 1.87)	0.96	18.9	0.33 – 2.98	0.70	No	No downgrade	Downgrade	No downgrade	No downgrade	No downgrade	Moderate	ns (Non-significant)
Guo al, 2016	Cancer	GLP-1RA	Placebo or active control	26	80/8364 (0.96%)	43/6074 (0.71%)	1.16 (0.78 – 1.71)	0.46	0	0.78 – 1.71	0.70	No	No downgrade	Downgrade	No downgrade	No downgrade	No downgrade	Moderate	ns (Non-significant)
Alves et al, 2012	Overall cancer	GLP-1RA	Placebo or active control	20	46/5613 (0.82%)	21/3989 (0.53%)	1.17 (0.68 – 2.02)	0.57	0	0.68 – 2.02	0.22	No	Downgrade	Downgrade	No downgrade	No downgrade	No downgrade	Low	ns (Non-significant)
**Adverse Event Outcomes**																			
Arrowaili et al, 2025	Serious adverse events	GLP-1RA	Placebo or no drugs	3	33/159 (20.75%)	16/107 (14.95%)	0.97 (0.47 – 2.00)	0.94	0	0.47 – 2.00	0.50	No	No downgrade	Downgrade	No downgrade	No downgrade	No downgrade	Moderate	ns (Non-significant)
Arrowaili et al, 2025	Adverse events	GLP-1RA	Placebo or no drugs	3	115/159 (72.33%)	39/107 (36.45%)	5.69 (2.52 – 12.87)	*<0.001*	25.6	1.89 – 17.12	0.72	Yes	No downgrade	Downgrade	No downgrade	No downgrade	No downgrade	Moderate	Class IV (Weak)
Badran et al, 2025	Loss of appetite	GLP-1RA	Placebo	3	56/298 (18.79%)	13/210 (6.19%)	3.19 (1.57 – 6.49)	0.001	24.5	1.24 – 8.24	0.79	No	No downgrade	No downgrade	No downgrade	No downgrade	No downgrade	High	Class IV (Weak)
Badran et al, 2025	Nausea	GLP-1RA	Placebo	4	185/376 (49.20%)	55/288 (19.10%)	2.47 (1.84 – 3.34)	*<0.001*	22.5	1.64 – 3.74	0.61	Yes	No downgrade	No downgrade	No downgrade	No downgrade	No downgrade	High	Class IV (Weak)
Badran et al, 2025	Vomiting	GLP-1RA	Placebo	4	46/376 (12.23%)	6/288 (2.08%)	4.46 (1.92 – 10.36)	*<0.001*	0	1.92 – 10.36	0.61	Yes	No downgrade	Downgrade	No downgrade	No downgrade	No downgrade	Moderate	Class IV (Weak)

© 2026 Yang K et al. *JAMA Network Open*

Sillassen et al, 2025	Serious adverse events	Semaglutide	Placebo	47	4828/18806 (25.67%)	4805/13259 (36.24%)	0.97 (0.85–1.11)	0.68	78.3	0.55–1.71	0.02	Yes	No downgrade	Downgrade	Downgrade	No downgrade	Downgrade	Very low	ns (Non-significant)
Patoulias et al, 2021	Upper respiratory tract infection	GLP-1RA	Placebo	6	116/20599 (0.56%)	144/20607 (0.70%)	0.81 (0.64–1.02)	0.08	0	0.64–1.02	0.86	No	No downgrade	Downgrade	No downgrade	No downgrade	No downgrade	Moderate	ns (Non-significant)
Patoulias et al, 2021	Lower respiratory tract infection	GLP-1RA	Placebo	6	33/20599 (0.16%)	32/20607 (0.16%)	1.03 (0.63–1.68)	0.90	0	0.63–1.68	0.95	No	No downgrade	Downgrade	No downgrade	No downgrade	No downgrade	Moderate	ns (Non-significant)
Dimitrios et al, 2020	Vomiting	Liraglutide	Placebo	4	98/742 (13.21%)	17/293 (5.80%)	2.07 (0.91–4.72)	0.08	34	0.58–7.37	0.19	Yes	No downgrade	Downgrade	No downgrade	No downgrade	No downgrade	Moderate	ns (Non-significant)
Dimitrios et al, 2020	Nausea	Liraglutide	Placebo	6	779/1802 (43.23%)	93/656 (14.18%)	5.19 (3.46–7.79)	< 0.001	39.3	2.59–10.43	0.22	Yes	No downgrade	Downgrade	No downgrade	No downgrade	No downgrade	Moderate	Class I (Convincing)
Dutta et al, 2022	Treatment-emergent adverse events	Semaglutide	Placebo or active control	4	945/1042 (90.69%)	951/1073 (88.63%)	2.26 (0.85–5.97)	0.10	76	0.34–15.08	0.15	No	Downgrade	Downgrade	Downgrade	No downgrade	No downgrade	Very low	ns (Non-significant)
Dutta et al, 2022	Severe adverse events	Semaglutide	Placebo or active control	4	318/1042 (30.52%)	351/1073 (32.71%)	1.11 (0.68–1.79)	0.69	41.8	0.50–2.45	0.08	Yes	Downgrade	Downgrade	No downgrade	No downgrade	No downgrade	Low	ns (Non-significant)
Dutta et al, 2022	Constipation	Semaglutide	Placebo or active control	3	50/216 (23.15%)	18/249 (7.23%)	3.79 (1.61–8.93)	0.002	47.4	1.01–14.32	0.75	Yes	Downgrade	No downgrade	No downgrade	No downgrade	No downgrade	Moderate	Class IV (Weak)
Dutta et al, 2022	Diarrhea	Semaglutide	Placebo or active control	4	213/1042 (20.44%)	133/1073 (12.40%)	2.05 (1.16–3.61)	0.01	66.8	0.71–5.91	0.69	Yes	Downgrade	No downgrade	Downgrade	No downgrade	No downgrade	Low	Class IV (Weak)
Dutta et al, 2022	Decreased appetite	Semaglutide	Placebo or active control	3	46/216 (21.30%)	12/249 (4.82%)	5.25 (2.68–10.29)	< 0.001	0	2.68–10.29	0.17	Yes	Downgrade	Downgrade	No downgrade	No downgrade	No downgrade	Low	Class IV (Weak)

© 2026 Yang K et al. *JAMA Network Open*

Dutta et al, 2022	Vomiting	Semaglutide	Placebo or active control	4	126/1042 (12.09%)	51/1073 (4.75%)	3.81 (1.76 – 8.25)	< 0.001	50.8	1.03 – 14.10	0.01	Yes	Downgrade	No downgrade	Downgrade	No downgrade	Downgrade	Very low	Class III (Suggestive)
Dutta et al, 2022	Nausea	Semaglutide	Placebo or active control	4	111/1042 (10.65%)	38/1073 (3.54%)	4.98 (3.23 – 7.67)	< 0.001	0	3.23 – 7.67	0.36	Yes	Downgrade	Downgrade	No downgrade	No downgrade	No downgrade	Low	Class I (Convincing)
Dutta et al, 2021	Treatment-emergent adverse events vs active comparator group	Tirzepatide	Placebo or active control	3	145/201 (72.14%)	116/192 (60.42%)	2.26 (0.88 – 5.81)	0.09	73	0.43 – 12.02	0.17	No	Downgrade	Downgrade	Downgrade	No downgrade	No downgrade	Very low	ns (Non-significant)
Dutta et al, 2021	Severe adverse events vs active comparator group	Tirzepatide	Placebo or active control	3	6/201 (2.99%)	4/192 (2.08%)	1.24 (0.33 – 4.62)	0.75	0	0.33 – 4.62	0.60	No	Downgrade	Downgrade	No downgrade	No downgrade	No downgrade	Low	ns (Non-significant)
Dutta et al, 2021	Severe adverse events vs placebo comparator group	Tirzepatide	Placebo or active control	4	102/1208 (8.44%)	234/1883 (12.43%)	1.00 (0.64 – 1.57)	1.00	49.9	0.46 – 2.16	0.82	No	Downgrade	Downgrade	No downgrade	No downgrade	No downgrade	Low	ns (Non-significant)
Dutta et al, 2021	Treatment-emergent adverse events vs placebo comparator group	Tirzepatide	Placebo or active control	4	851/1208 (70.45%)	1213/1883 (64.42%)	1.43 (1.12 – 1.82)	0.004	48.7	0.95 – 2.15	0.98	No	Downgrade	No downgrade	No downgrade	No downgrade	No downgrade	Moderate	Class IV (Weak)
Han et al, 2025	Nonserious infections	GLP-1RA	Placebo or active control	122	18169/72699 (24.99%)	13490/54487 (24.76%)	0.91 (0.85 – 0.97)	0.002	90.9	0.51 – 1.62	0.03	No	No downgrade	No downgrade	Downgrade	No downgrade	Downgrade	Low	Class IV (Weak)
Han et al, 2025	Total infections	GLP-1RA	Placebo or active control	136	22076/92152 (23.96%)	17435/72129 (24.17%)	0.89 (0.84	< 0.001	92.3	0.50 – 1.60	0.11	Yes	No downgrade	No downgrade	Downgrade	No downgrade	No downgrade	Moderate	Class III (Suggestive)

© 2026 Yang K et al. *JAMA Network Open*

							– 0.95)											
Han et al, 2025	Infections as important medical events	GLP-1RA	Placebo or active control	121	2144/89251 (2.40%)	2225/69695 (3.19%)	0.87 (0.81 – 0.92)	< 0.001	1.8	0.77 – 0.97	0.73	Yes	No downgrade	No downgrade	No downgrade	No downgrade	No downgrade	High	Class III (Suggestive)
Han et al, 2025	Serious infections	GLP-1RA	Placebo or active control	262	8419/180028 (4.68%)	8595/141993 (6.05%)	0.89 (0.87 – 0.92)	< 0.001	0	0.87 – 0.92	0.33	Yes	No downgrade	No downgrade	No downgrade	No downgrade	No downgrade	High	Class I (Convincing)
Helal et al, 2025	Diarrhea	GLP-1RA	Placebo or usual care or no treatment	4	39/263 (14.83%)	20/158 (12.66%)	1.38 (0.84 – 2.27)	0.20	0	0.84 – 2.27	0.38	No	No downgrade	Downgrade	No downgrade	No downgrade	No downgrade	Moderate	ns (Non-significant)
Helal et al, 2025	Fatigue	GLP-1RA	Placebo or usual care or no treatment	3	32/290 (11.03%)	13/183 (7.10%)	1.60 (0.56 – 4.55)	0.38	34	0.34 – 7.53	0.31	No	No downgrade	Downgrade	No downgrade	No downgrade	No downgrade	Moderate	ns (Non-significant)
Helal et al, 2025	Headache	GLP-1RA	Placebo or usual care or no treatment	3	44/290 (15.17%)	21/183 (11.48%)	1.19 (0.73 – 1.93)	0.48	0	0.73 – 1.93	0.60	No	No downgrade	Downgrade	No downgrade	No downgrade	No downgrade	Moderate	ns (Non-significant)
Helal et al, 2025	Constipation	GLP-1RA	Placebo or usual care or no treatment	4	60/263 (22.81%)	31/158 (19.62%)	1.48 (1.10 – 1.99)	0.01	0	1.10 – 1.99	0.30	No	No downgrade	No downgrade	No downgrade	No downgrade	No downgrade	High	Class IV (Weak)
Helal et al, 2025	Vomiting	GLP-1RA	Placebo or usual care or no treatment	3	17/151 (11.26%)	2/128 (1.56%)	4.84 (1.44 – 16.25)	0.01	0	1.44 – 16.25	0.57	Yes	No downgrade	Downgrade	No downgrade	No downgrade	No downgrade	Moderate	Class IV (Weak)

© 2026 Yang K et al. *JAMA Network Open*

Helal et al, 2025	Dyspepsia	GLP-1RA	Placebo or usual care or no treatment	3	38/290 (13.10%)	7/183 (3.83%)	2.72 (1.24 – 5.98)	0.01	0	1.24 – 5.98	0.80	Yes	No downgrade	No downgrade	No downgrade	No downgrade	No downgrade	High	Class IV (Weak)
Hu et al, 2023	Diarrhea	Exenatide	Metformin	2	0/61 (0.00%)	8/61 (13.11%)	0.13 (0.02 – 1.06)	0.06	0	NA	NA	No	No downgrade	Downgrade	No downgrade	No downgrade	No downgrade	Moderate	ns (Non-significant)
Hu et al, 2023	Constipation	Exenatide	Metformin	2	0/61 (0.00%)	3/61 (4.92%)	0.33 (0.03 – 3.17)	0.34	0	NA	NA	No	No downgrade	Downgrade	No downgrade	No downgrade	No downgrade	Moderate	ns (Non-significant)
Hu et al, 2023	Stomach pain	Exenatide	Metformin	2	0/61 (0.00%)	3/61 (4.92%)	0.33 (0.03 – 3.17)	0.34	0	NA	NA	No	No downgrade	Downgrade	No downgrade	No downgrade	No downgrade	Moderate	ns (Non-significant)
Hu et al, 2023	Vomiting	Exenatide	Metformin	3	8/106 (7.55%)	5/94 (5.32%)	1.41 (0.47 – 4.19)	0.54	0	0.47 – 4.19	0.61	No	No downgrade	Downgrade	No downgrade	No downgrade	No downgrade	Moderate	ns (Non-significant)
Hu et al, 2023	Nausea	Exenatide	Metformin	3	16/106 (15.09%)	12/94 (12.77%)	1.14 (0.56 – 2.31)	0.71	0	0.56 – 2.31	0.90	No	No downgrade	Downgrade	No downgrade	No downgrade	No downgrade	Moderate	ns (Non-significant)
Hu et al, 2023	Headache	Exenatide	Metformin	2	4/65 (6.15%)	4/53 (7.55%)	0.79 (0.21 – 3.01)	0.73	0	NA	NA	No	No downgrade	Downgrade	No downgrade	No downgrade	No downgrade	Moderate	ns (Non-significant)
Huang et al, 2025	Overall gastrointestinal adverse events	GLP-1RA	Placebo or other antidiabetic medications	18	2766/5794 (47.74%)	1644/4684 (35.10%)	2.62 (1.91 – 3.60)	< 0.001	89.5	0.79 – 8.71	0.02	No	No downgrade	No downgrade	Downgrade	No downgrade	Downgrade	Low	Class III (Suggestive)
Avgerinos et al, 2020	Nausea	GLP-1RA	Placebo	11	717/5871 (12.21%)	170/3806 (4.47%)	3.20 (1.75 – 5.84)	< 0.001	89.1	0.47 – 21.97	0.05	Yes	No downgrade	No downgrade	Downgrade	No downgrade	Downgrade	Low	Class III (Suggestive)

© 2026 Yang K et al. *JAMA Network Open*

Avgerinos et al, 2020	Diarrhea	GLP-1RA	Placebo	11	494/5871 (8.41%)	144/3806 (3.78%)	1.94 (1.52 – 2.49)	< 0.001	29.5	1.18 – 3.21	0.09	Yes	No downgrade	No downgrade	No downgrade	No downgrade	No downgrade	High	Class II (Highly Suggestive)
Avgerinos et al, 2020	Vomiting	GLP-1RA	Placebo	11	334/5871 (5.69%)	70/3806 (1.84%)	2.78 (1.91 – 4.06)	< 0.001	42.5	1.17 – 6.64	0.01	Yes	No downgrade	No downgrade	No downgrade	No downgrade	Downgrade	Moderate	Class II (Highly Suggestive)
Khan et al, 2025	Diarrhea	GLP-1RA	Placebo or usual care	5	34/256 (13.28%)	25/236 (10.59%)	1.28 (0.80 – 2.07)	0.30	0	0.80 – 2.07	0.22	No	Downgrade	Downgrade	No downgrade	No downgrade	No downgrade	Low	ns (Non-significant)
Khan et al, 2025	Back pain	GLP-1RA	Placebo or usual care	4	14/171 (8.19%)	17/152 (11.18%)	0.76 (0.38 – 1.52)	0.44	0	0.38 – 1.52	0.02	No	Downgrade	Downgrade	No downgrade	No downgrade	Downgrade	Very low	ns (Non-significant)
Khan et al, 2025	Anxiety	GLP-1RA	Placebo or usual care	4	6/178 (3.37%)	3/158 (1.90%)	1.66 (0.44 – 6.33)	0.46	0	0.44 – 6.33	0.05	No	Downgrade	Downgrade	No downgrade	No downgrade	No downgrade	Low	ns (Non-significant)
Khan et al, 2025	Urinary tract infection	GLP-1RA	Placebo or usual care	5	13/256 (5.08%)	13/236 (5.51%)	0.90 (0.37 – 2.21)	0.82	12.8	0.28 – 2.86	0.90	No	Downgrade	Downgrade	No downgrade	No downgrade	No downgrade	Low	ns (Non-significant)
Khan et al, 2025	Injection site reaction	GLP-1RA	Placebo or usual care	4	55/178 (30.90%)	43/158 (27.22%)	1.00 (0.84 – 1.19)	0.99	0	0.84 – 1.19	0.42	No	Downgrade	Downgrade	No downgrade	No downgrade	No downgrade	Low	ns (Non-significant)
Khan et al, 2025	Vomiting	GLP-1RA	Placebo or usual care	4	21/236 (8.90%)	3/212 (1.42%)	4.62 (1.60 – 13.29)	0.005	0	1.60 – 13.29	0.49	No	Downgrade	No downgrade	No downgrade	No downgrade	No downgrade	Moderate	Class IV (Weak)
Khan et al, 2025	Constipation	GLP-1RA	Placebo or usual care	4	46/178 (25.84%)	31/158 (19.62%)	1.44 (1.07 – 1.95)	0.02	0	1.07 – 1.95	0.19	No	Downgrade	No downgrade	No downgrade	No downgrade	No downgrade	Moderate	Class IV (Weak)
Khan et al, 2025	Nausea	GLP-1RA	Placebo or usual care	5	122/256 (47.66%)	46/236 (19.49%)	2.30 (1.60 – 3.31)	< 0.001	33.4	1.28 – 4.16	0.64	Yes	Downgrade	No downgrade	No downgrade	No downgrade	No downgrade	Moderate	Class IV (Weak)

© 2026 Yang K et al. *JAMA Network Open*

Konwar et al, 2022	Serious adverse events	Liraglutide	Placebo or active control	11	240/3950 (6.08%)	134/2561 (5.23%)	1.13 (0.90 – 1.40)	0.29	0.8	0.89 – 1.42	0.32	No	No downgrade	Downgrade	No downgrade	No downgrade	No downgrade	Moderate	ns (Non-significant)
Konwar et al, 2022	Adverse events	Liraglutide	Placebo or active control	11	3358/3985 (84.27%)	1890/2561 (73.80%)	1.09 (1.04 – 1.15)	< 0.001	76.6	0.94 – 1.27	0.26	Yes	No downgrade	No downgrade	Downgrade	No downgrade	No downgrade	Moderate	Class III (Suggestive)
Li et al, 2021	Adverse events vs placebo	Oral semaglutide	Placebo or active control	5	907/1257 (72.16%)	481/714 (67.37%)	1.07 (0.99 – 1.16)	0.10	38.3	0.94 – 1.22	0.79	No	Downgrade	Downgrade	No downgrade	No downgrade	No downgrade	Low	ns (Non-significant)
Li et al, 2021	Adverse events vs active comparator	Oral semaglutide	Placebo or active control	6	1736/2236 (77.64%)	1139/1522 (74.84%)	1.03 (0.97 – 1.10)	0.31	57.9	0.91 – 1.17	0.36	Yes	Downgrade	Downgrade	Downgrade	No downgrade	No downgrade	Very low	ns (Non-significant)
Li et al, 2021	Serious adverse events vs active comparator	Oral semaglutide	Placebo or active control	6	187/2236 (8.36%)	142/1522 (9.33%)	0.93 (0.71 – 1.22)	0.62	23.5	0.62 – 1.41	0.13	No	Downgrade	Downgrade	No downgrade	No downgrade	No downgrade	Low	ns (Non-significant)
Li et al, 2021	Serious adverse events vs placebo	Oral semaglutide	Placebo or active control	6	391/2848 (13.73%)	419/2306 (18.17%)	0.85 (0.75 – 0.96)	0.01	0	0.75 – 0.96	0.77	Yes	Downgrade	No downgrade	No downgrade	No downgrade	No downgrade	Moderate	Class IV (Weak)
Mostafa et al, 2025	Headache	ROSE-010	Placebo or usual care	6	39/547 (7.13%)	14/524 (2.67%)	2.57 (1.32 – 5.02)	0.01	0	1.32 – 5.02	0.22	No	Downgrade	No downgrade	No downgrade	No downgrade	No downgrade	Moderate	Class IV (Weak)
Mostafa et al, 2025	Vomiting	ROSE-010	Placebo or usual care	6	34/547 (6.22%)	2/524 (0.38%)	10.03 (3.35 – 30.02)	< 0.001	0	3.35 – 30.02	0.51	No	Downgrade	No downgrade	No downgrade	No downgrade	No downgrade	Moderate	Class III (Suggestive)
Mostafa et al, 2025	Pain relief	ROSE-010	Placebo or usual care	10	222/524 (42.37%)	114/500 (22.80%)	2.79 (2.02 – 3.84)	< 0.001	13.2	1.71 – 4.54	0.92	Yes	Downgrade	No downgrade	No downgrade	No downgrade	No downgrade	Moderate	Class I (Convincing)

© 2026 Yang K et al. *JAMA Network Open*

Mostafa et al, 2025	Nausea	ROSE-010	Placebo or usual care	6	151/547 (27.61%)	0/524 (0.00%)	78.50 (24.02–256.54)	< 0.001	0	24.02–256.54	0.59	Yes	Downgrade	No downgrade	No downgrade	No downgrade	No downgrade	Moderate	Class I (Convincing)
Taj et al, 2025	Injection-site reactions	GLP-1RA	Placebo or no drugs	14	396/4861 (8.15%)	62/3032 (2.04%)	3.53 (2.38–5.25)	< 0.001	47.3	1.25–9.99	0.38	No	No downgrade	No downgrade	No downgrade	No downgrade	No downgrade	High	Class III (Suggestive)
Ye et al, 2023	Gastrointestinal reaction	Exenatide	Metformin	6	51/256 (19.92%)	62/259 (23.94%)	0.87 (0.50–1.50)	0.61	51.4	0.30–2.47	0.75	No	No downgrade	Downgrade	Downgrade	No downgrade	No downgrade	Low	ns (Non-significant)
Ye et al, 2023	Other adverse reactions	Exenatide	Metformin	4	10/201 (4.98%)	7/202 (3.47%)	1.44 (0.23–8.92)	0.70	50.8	0.06–33.80	0.30	No	No downgrade	Downgrade	Downgrade	No downgrade	No downgrade	Low	ns (Non-significant)

CI, Confidence Interval; GRADE, Grading of Recommendations Assessment, Development and Evaluation; GLP-1RAs: Glucagon-like peptide-1 receptor agonists; NA, not available; No., number; PI, prediction interval;

a. Study limitation: we downgrade by one level when the contributions from high risk of bias (RoB) comparisons are higher than 50%.

b. Imprecision: If the metrics are odds ratio, we downgrade by one level if: 1) the total sample size is less than 300; or 2) the point estimate crossed the line of no effect (null value).

c. Inconsistency: we downgrade by one level if the heterogeneity $I^2 > 50\%$.

d. Indirectness: we downgrade by one level if there are differences in the PICO of the original studies included between different associations.

e. Publication bias: we downgrade by one level if the P-value of Harbord's test < 0.05.

© 2026 Yang K et al. *JAMA Network Open*

eTable 5. AMSTAR 2 Quality Assessment by Health Outcome Domain.

Author, Year	*1	*2	*3	*4	*5	*6	*7	*8	*9	*10	*11	*12	*13	*14	*15	*16
Zhang et al, 2025	2	2	2	1	0	2	0	2	2	0	2	0	0	2	2	2
Tan et al, 2025	2	2	2	2	2	2	1	2	2	2	2	1	1	2	2	2
Mabilleau et al, 2014	2	0	2	1	2	1	0	2	1	0	2	1	1	2	1	0
Su et al, 2015	2	1	2	1	2	2	0	2	1	0	2	0	1	2	2	2
Cheng et al, 2019	2	0	2	2	2	2	1	2	2	2	2	1	2	1	1	1
Zhang et al, 2024	2	2	2	2	2	2	1	2	2	0	2	0	1	2	2	2
Yu et al, 2023	2	2	2	2	2	2	2	2	2	2	2	2	1	2	2	2
Wei et al, 2021	2	0	2	1	0	0	0	1	0	0	2	0	0	2	0	2
Seminer et al, 2025	2	2	1	2	2	2	1	2	2	2	2	2	2	2	2	2
Sindhu et al, 2024	2	1	2	1	2	2	1	2	2	2	2	1	2	2	2	2
Tang et al, 2023	2	0	2	1	2	0	0	2	2	0	2	1	2	2	2	2
Chen et al, 2025	2	2	1	1	0	0	1	2	2	0	2	0	1	2	2	2
Ebrahimi et al, 2025	2	2	2	2	2	2	1	2	2	0	2	2	2	2	2	2
Silverii et al, 2024	2	2	1	2	0	2	2	2	2	0	2	0	1	1	2	2
Hu et al, 2022	2	2	1	2	0	2	1	2	2	0	2	0	2	2	2	2
Mantovani et al, 2025	2	2	2	2	2	2	1	2	2	0	2	0	1	2	2	2
Safwan et al, 2025	2	2	2	2	2	2	2	2	2	2	2	1	2	2	2	2
Wang et al, 2024	2	0	2	2	2	2	2	2	2	0	2	2	2	2	2	2
Muhammed et al, 2024	2	2	2	1	1	2	1	2	1	0	2	1	1	2	2	2
Nreu et al, 2020	2	2	2	1	2	2	1	2	2	2	2	0	2	2	2	2
He et al, 2022	2	2	2	2	2	2	0	2	2	2	2	1	2	2	2	2
Nreu et al, 2020	2	2	2	2	2	2	1	2	2	0	2	1	2	2	2	2
Monami et al, 2017	2	2	2	1	2	2	1	2	2	0	2	1	2	2	2	2
Monami et al, 2014	2	0	2	1	2	2	1	2	2	0	2	0	1	1	2	2

© 2026 Yang K et al. *JAMA Network Open*

Alves et al, 2012	2	0	2	2	2	2	1	2	2	2	2	1	2	2	1	2
Chiang et al, 2025	2	2	2	2	2	2	0	2	2	2	2	2	2	2	2	1
Masson et al, 2024	2	2	1	1	2	1	0	2	2	0	2	0	1	1	2	2
Wen et al, 2025	2	2	2	2	2	2	0	2	2	0	2	0	1	2	0	2
Wu et al, 2025	2	2	2	2	2	2	1	2	2	0	2	2	2	2	2	2
Silverii et al, 2025	2	2	2	2	2	2	2	2	2	0	2	1	1	2	2	2
Nagendra et al, 2023	2	2	2	2	2	2	1	2	2	2	2	1	1	2	2	2
Piccoli et al, 2021	2	2	2	1	2	2	1	2	2	1	2	1	2	2	2	2
Liu et al, 2019	2	2	2	1	2	2	1	2	2	0	2	1	2	2	2	2
Guo et al, 2016	2	0	2	2	2	2	1	2	0	2	2	0	0	2	2	2
Silverii et al, 2024	2	2	2	2	2	2	2	2	2	1	2	2	2	2	2	2
Duchemin et al, 2025	2	2	2	2	2	2	1	2	2	2	2	2	2	2	2	2
Cao et al, 2019	2	2	2	2	0	2	1	2	2	0	2	1	2	2	2	2
Figlioli et al, 2024	2	2	0	2	2	2	1	2	2	0	2	0	2	2	2	2
Pinto et al, 2019	2	2	2	2	2	2	0	2	2	0	2	1	2	2	2	2
Ko et al, 2025	2	2	1	2	2	2	1	2	2	0	2	2	2	2	2	1
Rao et al, 2025	2	2	1	2	2	2	1	2	0	0	2	1	1	2	2	1
Cao et al, 2020	2	1	1	2	2	2	1	2	2	0	2	0	1	2	2	2
Ye et al, 2023	2	2	1	2	2	2	1	2	2	0	2	0	1	2	1	2
Han et al, 2025	2	2	1	2	2	2	1	2	2	0	2	0	1	2	2	2
Arrowaili et al, 2025	2	2	1	1	2	2	1	2	2	0	2	0	1	2	0	2
Patoulias et al, 2021	2	0	1	1	0	2	0	1	2	0	2	0	1	1	0	2
Taj et al, 2025	2	2	1	2	2	2	1	2	2	0	2	0	1	2	2	2
Helal et al, 2025	2	2	2	2	2	2	0	2	2	0	2	2	2	2	0	2
Huang et al, 2025	2	1	2	2	2	2	0	2	2	0	2	2	2	2	2	2
Badran et al, 2025	2	2	2	2	2	2	0	2	2	0	2	2	2	2	2	2
Dimitrios et al, 2020	2	0	1	2	2	2	1	2	2	0	2	0	1	1	1	2

© 2026 Yang K et al. *JAMA Network Open*

Dutta et al, 2021	2	2	2	2	2	2	2	2	2	2	2	1	2	2	2	2
Li et al, 2021	2	0	2	2	2	2	2	2	2	2	2	2	2	2	2	2
Konwar et al, 2022	2	2	2	1	2	2	0	2	2	0	2	0	0	2	2	2
Hu et al, 2023	2	2	2	1	2	2	2	2	2	0	2	1	2	2	0	2
Mostafa et al, 2025	2	2	2	1	2	2	0	2	2	0	2	0	2	2	0	2
Dutta et al, 2022	2	2	2	2	2	2	0	2	2	0	2	0	0	2	2	2
Khan et al, 2025	2	2	2	1	2	2	0	2	2	0	2	0	0	2	0	2
Sillassen et al, 2025	2	2	2	2	2	2	2	2	2	2	2	2	2	2	2	2
Avgerinos et al, 2020	2	0	2	2	2	2	0	2	2	2	2	2	2	2	2	2

0 = No; 1 = Partial Yes; 2 = Yes. Shaded columns = AMSTART 2 critical domains

**1. Did the research questions and inclusion criteria for the review include the components of PICO?*

*2. *Did the report of the review contain an explicit statement that the review methods were established prior to the conduct of the review and did the report justify any significant deviations from the protocol?*

*3. *Did the review authors explain their selection of the study designs for inclusion in the review?*

**4. Did the review authors use a comprehensive literature search strategy?*

**5. Did the review authors perform study selection in duplicate?*

**6. Did the review authors perform data extraction in duplicate?*

**7. Did the review authors provide a list of excluded studies and justify the exclusions?*

**8. Did the review authors describe the included studies in adequate detail?*

**9. Did the review authors use a satisfactory technique for assessing the risk of bias (RoB) in individual studies that were included in the review?*

**10. Did the review authors report on the sources of funding for the studies included in the review?*

**11. If meta-analysis was performed, did the review authors use appropriate methods for statistical combination of results?*

**12. If meta-analysis was performed, did the review authors assess the potential impact of RoB in individual studies on the results of the meta-analysis or other evidence synthesis?*

**13. Did the review authors account for RoB in primary studies when interpreting/discussing the results of the review?*

**14. Did the review authors provide a satisfactory explanation for, and discussion of, any heterogeneity observed in the results of the review?*

**15. If they performed quantitative synthesis did the review authors carry out an adequate investigation of publication bias (small study bias) and discuss its likely impact on the results of the review?*

**16. Did the review authors report any potential sources of conflict of interest, including any funding they received for conducting the review?*

© 2026 Yang K et al. *JAMA Network Open*

eTable 6. Sensitivity and Leave-One-Out Analyses of Associations Between Glucagon-Like Peptide-1 Receptor Agonists and Cancer Outcomes.

Author, year	Outcome	Events/participants (GLP-1RA)	Random-effects OR (95% CI)	Mantel-Haenszel OR (95% CI)	Peto OR (95% CI)	Largest absolute change in OR	Significance changed	Direction changed
Alves et al, 2012	Overall cancer	46/5613 (0.82%)	1.17 (0.68–2.02)	1.27 (0.78-2.09)	1.3 (0.78-2.17)	0.15	FALSE	FALSE
Cao et al, 2020	Pancreatic cancer	57/26357 (0.22%)	1.08 (0.63–1.86)	1.04 (0.96-1.13)	1.04 (0.96-1.13)	0.05	FALSE	TRUE
Cao et al, 2019	Overall cancer	1342/34102 (3.94%)	1.02 (0.92–1.13)	1.09 (0.73-1.62)	1.09 (0.72-1.66)	0.19	TRUE	FALSE
Cao et al, 2019	Thyroid cancer	34/22276 (0.15%)	1.39 (0.77–2.50)	1.67 (0.96-2.91)	1.75 (1-3.07)	0.16	FALSE	TRUE
Cao et al, 2019	Pancreatic cancer	48/25102 (0.19%)	0.97 (0.59–1.58)	1.12 (0.77-1.63)	1.12 (0.77-1.63)	0.16	FALSE	TRUE
Duchemin et al, 2025	Thyroid cancer	70/47588 (0.15%)	1.03 (0.69–1.53)	1.22 (0.87-1.71)	1.29 (0.89-1.89)	0.2	TRUE	FALSE
Figlioli et al, 2024	Gastrointestinal cancer	436/70646 (0.62%)	0.97 (0.85–1.12)	1.02 (0.89-1.16)	1.02 (0.88-1.17)	0.03	FALSE	TRUE
Guo et al, 2016	Cancer	80/8364 (0.96%)	1.16 (0.78–1.71)	1.22 (0.84-1.76)	1.23 (0.85-1.78)	0.06	FALSE	FALSE
He et al, 2022	Biliary cancer	24/31010 (0.08%)	1.45 (0.77–2.76)	1.44 (0.79-2.63)	1.51 (0.81-2.84)	0.18	FALSE	FALSE
Hu et al, 2022	Thyroid cancer	63/43124 (0.15%)	1.26 (0.82–1.92)	1.57 (1.06-2.32)	1.74 (1.14-2.65)	0.06	TRUE	FALSE
Ko et al, 2025	Thyroid Cancer	87/101749 (0.09%)	1.24 (0.88–1.77)	1.09 (0.84-1.41)	1.1 (0.84-1.44)	0.08	FALSE	FALSE
Ko et al, 2025	Pancreatic Cancer	136/81366 (0.17%)	0.93 (0.73–1.19)	1.22 (0.99-1.51)	1.23 (1-1.52)	0.04	FALSE	FALSE
Ko et al, 2025	Colorectal Cancer	196/41451 (0.47%)	1.20 (0.97–1.48)	0.92 (0.59-1.43)	0.91 (0.57-1.46)	NA	NA	NA
Ko et al, 2025	Gastric Cancer	53/35890 (0.15%)	1.04 (0.69–1.57)	0.79 (0.46-1.35)	0.78 (0.45-1.35)	0.15	FALSE	FALSE
Ko et al, 2025	Esophageal Cancer	23/25885 (0.09%)	0.80 (0.46–1.39)	1.23 (0.69-2.2)	1.29 (0.68-2.44)	0.17	FALSE	FALSE
Ko et al, 2025	Liver Cancer	43/35763 (0.12%)	0.86 (0.52–1.42)	1.12 (0.76-1.66)	1.13 (0.76-1.68)	0.1	FALSE	TRUE
Ko et al, 2025	Gallbladder Cancer	23/39134 (0.06%)	1.14 (0.60–2.15)	1.14 (0.85-1.55)	1.15 (0.85-1.56)	0.07	FALSE	FALSE
Ko et al, 2025	Breast Cancer	116/45638 (0.25%)	1.04 (0.79–1.36)	0.83 (0.56-1.25)	0.83 (0.55-1.25)	0.17	FALSE	FALSE
Ko et al, 2025	Ovarian Cancer	19/13235 (0.14%)	1.11 (0.53–2.32)	1.05 (0.45-2.45)	1.06 (0.43-2.58)	0.14	FALSE	TRUE
Ko et al, 2025	Endometrial Cancer	35/14804 (0.24%)	0.85 (0.52–1.40)	1.02 (0.61-1.7)	1.02 (0.59-1.75)	0.08	FALSE	FALSE
Ko et al, 2025	Kidney Cancer	91/38155 (0.24%)	1.09 (0.80–1.47)	1.44 (0.74-2.79)	1.57 (0.76-3.25)	0.21	FALSE	FALSE
Ko et al, 2025	Multiple Myeloma	27/35887 (0.08%)	1.03 (0.60–1.75)	0.97 (0.77-1.22)	0.97 (0.76-1.23)	0.07	FALSE	FALSE
Ko et al, 2025	Meningioma	11/32441 (0.03%)	1.04 (0.43–2.53)	1.63 (1.17-2.26)	1.83 (1.28-2.6)	0.07	TRUE	FALSE
Liu et al, 2019	Neoplasms	773/24702 (3.13%)	1.04 (0.94–1.15)	1.05 (0.95-1.17)	1.05 (0.95-1.17)	0.06	FALSE	FALSE
Muhammed et al, 2024	Pancreatic cancer	51/22250 (0.23%)	1.17 (0.65–2.12)	1.37 (0.9-2.1)	1.38 (0.9-2.1)	0.22	TRUE	FALSE

© 2026 Yang K et al. *JAMA Network Open*

Nagendra et al, 2023	Pancreatic cancer	6/25910 (0.02%)	0.51 (0.30–0.85)	1.2 (0.93-1.56)	1.21 (0.93-1.58)	0.04	TRUE	FALSE
Nagendra et al, 2023	Thyroid cancer	12/27204 (0.04%)	0.78 (0.46–1.31)	0.38 (0.17-0.88)	0.29 (0.12-0.73)	0.15	FALSE	FALSE
Nagendra et al, 2023	Neoplasms	210/39822 (0.53%)	0.96 (0.75–1.23)	2.39 (0.85-6.69)	3.24 (1.09-9.58)	0.12	FALSE	FALSE
Piccoli et al, 2021	Breast cancer	129/48267 (0.27%)	0.95 (0.74–1.23)	1.05 (0.82-1.34)	1.05 (0.81-1.37)	0.05	FALSE	FALSE
Pinto et al, 2019	Pancreatic cancer	37/18394 (0.20%)	0.98 (0.52–1.86)	1.08 (0.68-1.72)	1.09 (0.68-1.74)	0.27	FALSE	TRUE
Rao et al, 2025	Bladder cancer	25/31464 (0.08%)	0.74 (0.43–1.26)	0.69 (0.42-1.14)	0.68 (0.41-1.12)	0.22	FALSE	FALSE
Silverii et al, 2025	Overall cancer	1725/55305 (3.12%)	1.03 (0.97–1.10)	2.02 (1.34-3.05)	2.13 (1.42-3.18)	0.11	FALSE	FALSE
Silverii et al, 2025	Uterine cancer	34/35940 (0.09%)	0.77 (0.44–1.37)	1.31 (1.06-1.62)	1.32 (1.06-1.63)	0.04	FALSE	FALSE
Silverii et al, 2025	Endometrial Cancer	15/24348 (0.06%)	0.68 (0.35–1.31)	0.69 (0.36-1.3)	0.67 (0.35-1.29)	0.18	FALSE	FALSE
Silverii et al, 2025	Thyroid cancer	77/46412 (0.17%)	1.42 (0.95–2.13)	1.05 (0.98-1.13)	1.05 (0.98-1.13)	0.01	FALSE	FALSE
Silverii et al, 2025	Colorectal cancer	199/43557 (0.46%)	1.24 (1.00–1.54)	1.97 (1.34-2.9)	2.09 (1.42-3.06)	0.12	FALSE	FALSE
Silverii et al, 2024	Overall thyroid cancer	73/36886 (0.20%)	1.64 (1.07–2.50)	0.81 (0.53-1.25)	0.79 (0.5-1.25)	NA	NA	NA
Wen et al, 2025	Pancreatic cancer	36/36813 (0.10%)	0.91 (0.61–1.36)	0.94 (0.6-1.49)	0.94 (0.59-1.5)	0.54	FALSE	TRUE
Wu et al, 2025	Esophageal cancer	2/6507 (0.03%)	0.87 (0.20–3.82)	0.55 (0.15-2.09)	0.4 (0.08-2.02)	0.72	FALSE	TRUE

Abbreviations: CI, Confidence Interval; GLP-1RAs: Glucagon-like peptide-1 receptor agonists; NA, not available; No., number; OR, odds ratios; PI, prediction interval; OR, odds ratio.

© 2026 Yang K et al. *JAMA Network Open*

eTable 7. Studies Excluded from the Meta-Analysis with Rationale.

Number	Title
**Reason: Not a meta-analysis/systematic review with quantitative synthesis or not based on randomized controlled trials.**	
1	Incretin-based drugs decrease the incidence of prostate cancer in type 2 diabetics: A pooling-up analysis
2	Comparison of GLP-1 Receptor Agonists Combined with Metformin Versus Metformin Alone in the Management of PCOS: A Comprehensive Meta-Analysis
3	Expanding therapeutic horizons: glucagon-like peptide-1 receptor agonists and sodium glucose transporter-2 inhibitors in poly cystic ovarian syndrome: a comprehensive review including systematic review and network meta-analysis of randomized clinical trials
4	GLP-1 Receptor Agonist Induced Eustachian Tube Dysfunction: Database and Systematic Review of Otolaryngologic Adverse Events
5	Risk of Hearing Loss in Patients Treated with Exendin-4 Derivatives: A Network Meta-Analysis of Glucagon-like Peptide-1 Receptor Agonists and Sodium-Glucose Cotransporter 2 Inhibitors
6	Glucagon-Like Peptide 1 Receptor Agonists and Chronic Lower Respiratory Disease Among Type 2 Diabetes Patients: Replication and Reliability Assessment Across a Research Network
7	The Association Between Antidiabetic Agents and Clinical Outcomes of COVID-19 Patients With Diabetes: A Bayesian Network Meta-Analysis
8	Effect of glucagon-like peptide-1 receptor agonists in osteoarthritis: A systematic review of pre-clinical and human studies
9	Risk of bone fracture by using dipeptidyl peptidase-4 inhibitors, glucagon-like peptide-1 receptor agonists, or sodium-glucose cotransporter-2 inhibitors in patients with type 2 diabetes mellitus: a network meta-analysis of population-based cohort studies
10	Hypoglycemic agents and bone health; an umbrella systematic review of the clinical trials' meta-analysis studies
11	Effects of Incretin Therapy on Skeletal Health in Type 2 Diabetes-A Systematic Review
12	The Impact of GLP1 Agonists on Bone Metabolism: A Systematic Review
13	Glucose-Lowering Drugs and Fracture Risk-a Systematic Review
14	Glucagon-like peptide-1 receptor agonists and fracture risk: a network meta-analysis of randomized clinical trials
15	Glucagon-Like Peptide-1 Receptor Agonists and Thyroid Cancer: A Narrative Review
16	Assessment of Thyroid Carcinogenic Risk and Safety Profile of GLP1-RA Semaglutide (Ozempic) Therapy for Diabetes Mellitus and Obesity: A Systematic Literature Review
17	The pharmacodynamics-based prophylactic benefits of GLP-1 receptor agonists and SGLT2 inhibitors on neurodegenerative diseases: evidence from a network meta-analysis
18	Neurological Manifestation of Incretin-Based Therapies in Patients with Type 2 Diabetes: A Systematic Review and Network Meta-Analysis
19	GLP-1 receptor agonists cause gastrointestinal adverse reaction in patients with type 2 diabetes mellitus: a network meta-analysis
20	Impact of GLP-1 Receptor Agonists in Gastrointestinal Endoscopy: An Updated Review
21	A Scoping Review of GLP-1 Receptor Agonists: Are They Associated with Increased Gastric Contents, Regurgitation, and Aspiration Events?
22	Gastrointestinal adverse events with insulin glargine/lixisenatide fixed-ratio combination versus glucagon-like peptide-1 receptor agonists in people with type 2 diabetes mellitus: A network meta-analysis

© 2026 Yang K et al. *JAMA Network Open*

23	Gastrointestinal adverse events of glucagon-like peptide-1 receptor agonists in patients with type 2 diabetes: a systematic review and network meta-analysis
24	Efficacy of Hypoglycemic Agents in Metabolic Dysfunction Associated Steatotic Liver Disease (MASLD): A Systematic Review and Network Meta-Analysis
25	Pharmacological treatment options for Metabolic Dysfunction Associated Steatotic Liver Disease (MASLD) in patients with type 2 diabetes mellitus: A systematic review
26	Comparative Efficacy of Drug Interventions on NAFLD Over 24 Weeks: A Traditional and Network Meta-Analysis of Randomized Controlled Trials
27	Efficacy of pharmacologic interventions on magnetic resonance imaging biomarkers in patients with nonalcoholic fatty liver disease: systematic review and network meta-analysis
28	Comparative efficacy of THR-β agonists, FGF-21 analogues, GLP-1R agonists, GLP-1-based polyagonists, and Pan-PPAR agonists for MASLD: A systematic review and network meta-analysis
29	Comparative Analysis of Resmetirom vs. FGF21 Analogs vs. GLP-1 Agonists in MASLD and MASH: Network Meta-Analysis of Clinical Trials
30	PPAR-alpha/gamma agonists, glucagon-like peptide-1 receptor agonists and metformin for non-alcoholic fatty liver disease: A network meta-analysis
31	Comparing the effectiveness of long-term use of daily and weekly glucagon-like peptide-1 receptor agonists treatments in patients with nonalcoholic fatty liver disease and type 2 diabetes mellitus: a network meta-analysis
32	Comparison of glucagon-like peptide-1 receptor agonists and thiazolidinediones on treating nonalcoholic fatty liver disease: A network meta-analysis
33	New anti-diabetic agents for the treatment of non-alcoholic fatty liver disease: a systematic review and network meta-analysis of randomized controlled trials
34	Comparative efficacy of 5 sodium-glucose cotransporter protein-2 (SGLT-2) inhibitor and 4 glucagon-like peptide-1 (GLP-1) receptor agonist drugs in non-alcoholic fatty liver disease: A GRADE-assessed systematic review and network meta-analysis of randomized controlled trials
35	Comparative effectiveness of multiple different treatment regimens for nonalcoholic fatty liver disease with type 2 diabetes mellitus: a systematic review and Bayesian network meta-analysis of randomized controlled trials
36	GLP-1 RAs and SGLT-2 Inhibitors for Insulin Resistance in Nonalcoholic Fatty Liver Disease: Systematic Review and Network Meta-Analysis
37	Antidiabetic Medications for Type 2 Diabetics with Nonalcoholic Fatty Liver Disease: Evidence From a Network Meta-Analysis of Randomized Controlled Trials
38	Sodium-glucose cotransporter protein-2 inhibitors and glucagon-like peptide-1 receptor agonists versus thiazolidinediones for non-alcoholic fatty liver disease: A network meta-analysis
39	Review article: role of glucagon-like peptide-1 receptor agonists in non-alcoholic steatohepatitis, obesity and diabetes-what hepatologists need to know
40	Effectiveness of drug interventions in nonalcoholic fatty liver disease: A network meta-analysis
41	Novel glucose-lowering drugs for non-alcoholic fatty liver disease
42	Real-world evidence on the association of novel antidiabetic medication use with cancer risk and protective effects: a systematic review and network meta-analysis.
43	GLP-1 Receptor Agonists Use and Incidence of Glaucoma: A Systematic Review and Meta-Analysis
44	Glucagon-Like Peptide 1 Receptor Agonist (GLP1RA) Exposure and Outcomes in Type 2 Diabetes: A Systematic Review of Population-Based Observational Studies

© 2026 Yang K et al. *JAMA Network Open*

45	Association between glucagon-like peptide-1 receptor agonist use and peri-operative pulmonary aspiration: a systematic review and meta-analysis.
46	The Effect of Antihyperglycemic Medications on COVID-19: A Meta-analysis and Systematic Review from Observational Studies
47	Association between novel Glucose-Lowering drugs and risk of Asthma: A network Meta-Analysis of cardiorenal outcome trials
48	Risk of bone fracture by using dipeptidyl peptidase-4 inhibitors, glucagon-like peptide-1 receptor agonists, or sodium-glucose cotransporter-2 inhibitors in patients with type 2 diabetes mellitus: a network meta-analysis of population-based cohort studies
49	Risk of fracture with dipeptidyl peptidase-4 inhibitors, glucagon-like peptide-1 receptor agonists, or sodium-glucose cotransporter-2 inhibitors in real-world use: systematic review and meta-analysis of observational studies
50	Newer glucose-lowering drugs and risk of dementia: A systematic review and meta-analysis of observational studies
51	Glucagon-like peptide 1 (GLP-1) receptor agonists as a protective factor for incident depression in patients with diabetes mellitus: A systematic review
52	Glucagon-like peptide-1 receptor agonist use and the risk of residual gastric contents and aspiration in patients undergoing GI endoscopy: a systematic review and a meta-analysis
53	A systematic review and meta-analysis of observational studies of the association between the use of incretin-based therapies and the risk of pancreatic cancer
54	Using real-world data to evaluate the association of incretin-based therapies with risk of acute pancreatitis: a meta-analysis of 1,324,515 patients from observational studies
55	Incretin-based therapies and acute pancreatitis risk: a systematic review and meta-analysis of observational studies
56	Effect of Glucagon-Like Peptide-1 Receptor Agonists on Bowel Preparation for Colonoscopy: A Systematic Review and Meta-Analysis.
57	Influence of glucagon-like peptide-1 receptor agonists on hepatic events in type 2 diabetes: a systematic review and meta-analysis
58	Sodium-Glucose Cotransporter 2 Inhibitor Use and Risk of Liver-Related Events in Patients With Type 2 Diabetes: A Meta-analysis of Observational Cohort Studies
59	Glucagon-like peptide-1 receptor agonist use is associated with a lower risk of major adverse liver-related outcomes: a meta-analysis of observational cohort studies
60	Risk of Hepatocellular Carcinoma with Glucagon-like Peptide-1 receptor agonist treatment in patients: a systematic review and meta-analysis
61	Glucose-lowering drugs and liver-related outcomes among individuals with type 2 diabetes: A systematic review of longitudinal population-based studies
62	Tarar ZI, Farooq U, Chaudhry A, et al. Evidence Report on the Safety of Gastrointestinal Endoscopy in Patients on Glucagon-like Peptide-1 Receptor Agonists: A Systematic Review and Meta-Analysis.
63	Glucagon-like peptide-1 receptor agonists increase the risk of residual gastric content and pulmonary aspiration on upper endoscopy: A meta-analysis.
64	Effects of glucagon-like peptide-1 receptor agonists on endoscopy outcomes: systematic review and meta-analysis.
65	The impact of glucagon-like peptide-1 receptor agonists on the quality indicators of colonoscopy - a systematic review and meta-analysis. Dig Liver Dis.
66	Effect of Glucagon-Like Peptide-1 Receptor Agonists on Bowel Preparation for Colonoscopy: A Systematic Review and Meta-Analysis.
67	Effect of diabetes medications on the risk of developing dementia, mild cognitive impairment, or cognitive decline: A systematic review and meta-analysis
68	Frequency of acute pancreatitis associated with glucagon-like peptide-1 use: A systematic review.
69	A systematic review and meta-analysis of observational studies of the association between the use of incretin-based therapies and the risk of pancreatic cancer.

© 2026 Yang K et al. *JAMA Network Open*

70	Meta-analysis of observational studies on the effect of incretin treatment on fracture risk.
71	Bone fracture risk is not associated with the use of glucagon-like peptide-1 receptor agonists: a population-based cohort analysis.
72	The use of incretins and fractures - a meta-analysis on population-based real-life data.
73	Risk of fracture with dipeptidyl peptidase-4 inhibitors, glucagon-like peptide-1 receptor agonists, or sodium-glucose cotransporter-2 inhibitors in real-world use: systematic review and meta-analysis of observational studies.
74	GLP-1 and GIP receptor agonists in the treatment of Parkinson's disease: Translational systematic review and meta-analysis protocol of clinical and preclinical studies.
75	Cardiovascular outcomes, heart failure and mortality in type 2 diabetic patients treated with glucagon-like peptide 1 receptor agonists (GLP-1 RAs): A systematic review and meta-analysis of observational cohort studies.
76	Using real-world data to evaluate the association of incretin-based therapies with risk of acute pancreatitis: a meta-analysis of 1,324,515 patients from observational studies.
77	A systematic review of acute pancreatitis as an adverse event of type 2 diabetes drugs: From hard facts to a balanced position.
**Reason: Insufficient or inadequate data for quantitative synthesis**	
78	Adverse drug reactions of GLP-1 agonists: A systematic review of case reports
79	A meta-analysis of the incidence of acne vulgaris in patients treated with GLP-1 agonists
80	The Therapeutic Potential of GLP-1 Receptor Agonists in the Management of Hidradenitis Suppurativa: A Systematic Review of Anti-Inflammatory and Metabolic Effects
81	The effect of glucagon-like peptide-1 (GLP-1) receptor agonists on substance use disorder (SUD)-related behavioral effects of drugs and alcohol: A systematic review
82	The effect of glucagon-like Peptide-1 receptor agonists on measures of suicidality: A systematic review
83	The effect of GLP-1RAs on mental health and psychotropics-induced metabolic disorders: A systematic review
84	A systematic review in effects of glucagon-like peptide-1 (GLP-1) mono-agonists on functional connectivity: Target engagement and rationale for the development in mental disorders
85	Glucagon-like peptide 1 agonist and effects on reward behavior: A systematic review
86	GLP-1 and GIP receptor agonists in the treatment of Parkinson's disease: Translational systematic review and meta-analysis protocol of clinical and preclinical studies
87	Impact of antidiabetic agents on dementia risk: A Bayesian network meta-analysis
88	The effects of dipeptidyl peptidase-4 inhibitors and glucagon-like peptide 1 receptor agonists on cognitive functions in adults with type 2 diabetes mellitus: a systematic review and meta-analysis
89	GLP-1R Agonists and Their Therapeutic Potential in Inflammatory Bowel Disease and Other Immune-Mediated Inflammatory Diseases, a Systematic Review of the Literature
90	Effects of GLP-1 Analogues and Agonists on the Gut Microbiota: A Systematic Review

© 2026 Yang K et al. *JAMA Network Open*

91	Association Between GLP1 RAs Use and Risk of Colorectal Cancer: A Systematic Review and Meta-Analysis
92	GLP-1 receptor agonists: A novel pharmacotherapy for binge eating (Binge eating disorder and bulimia nervosa)? A systematic review
93	GLP-1RAs caused gastrointestinal adverse reactions of drug withdrawal: a system review and network meta-analysis
94	Incretin-based glucose-lowering medications and the risk of acute pancreatitis and malignancies: a meta-analysis based on cardiovascular outcomes trials
95	The impact of gut hormones on the neural circuit of appetite and satiety: A systematic review
96	Incretin-Based Therapy and Risk of Pancreatic Cancer in Patients with Type 2 Diabetes Mellitus: A Meta-analysis of Randomized Controlled Trials
97	A systematic review of acute pancreatitis as an adverse event of type 2 diabetes drugs: from hard facts to a balanced position
98	Impact of GLP-1 receptor agonists on major gastrointestinal disorders for type 2 diabetes mellitus: a mixed treatment comparison meta-analysis
99	The role of glucagon-like peptide-1 receptor agonists in metabolic dysfunction-associated steatohepatitis
100	Efficacy of peroxisome proliferator-activated receptor agonists, glucagon-like peptide-1 receptor agonists, or sodium-glucose cotransporter-2 inhibitors for treatment of non-alcoholic fatty liver disease: a systematic review
101	A Systematic Review of Newer Antidiabetic Agents in the Treatment of Nonalcoholic Fatty Liver Disease
102	Efficacy of Glucagon-Like Peptide-1 Analogs in Nonalcoholic Fatty Liver Disease: A Systematic Review
103	Effect of Weight Loss Medications on Hepatic Steatosis and Steatohepatitis: A Systematic Review
104	Efficacy and safety of anti-hyperglycemic drugs in patients with non-alcoholic fatty liver disease with or without diabetes: An updated systematic review of randomized controlled trials
105	Glucagon-like peptide-1 receptor agonists (GLP-1 RAs) for the management of nonalcoholic fatty liver disease (NAFLD): A systematic review
106	Treating nonalcoholic fatty liver disease in patients with type 2 diabetes mellitus: a review of efficacy and safety
107	Comparative efficacy of anti-diabetic agents on nonalcoholic fatty liver disease in patients with type 2 diabetes mellitus: a systematic review and meta-analysis of randomized and non-randomized studies
108	Glucagon-Like Peptide 1 Receptor Agonists and Mental Health: A Systematic Review and Meta-Analysis. JAMA Psychiatry.
109	The Association between GLP-1 Receptor-Based Agonists and the Incidence of Asthma in Patients with Type 2 Diabetes and/or Obesity: A Meta-Analysis.
110	Use of GLP1RAs and occurrence of respiratory disorders: A meta-analysis of large randomized trials of GLP1RAs.
111	Impact of glucagon-like peptide-1 receptor agonists on nasopharyngitis and upper respiratory tract infection among patients with type 2 diabetes: a network meta-analysis
112	Effects of glucagon-like PEPTIDE-1 receptor agonists on incidence of hepatocellular carcinoma and liver decompensation in patients with diabetes: A systematic review and META-analysis
**Reason: Incorrect intervention**	
113	Impact of SGLT-2i on COPD exacerbations in patients with type 2 diabetes mellitus: A systematic review and meta-analysis
114	Evaluation of the risk of fracture in type 2 diabetes mellitus patients with incretins: an updated meta-analysis
115	Dipeptidyl peptidase-4 inhibitors and gallbladder or biliary disease in type 2 diabetes: systematic review and pairwise and network meta-analysis of randomized controlled trials

© 2026 Yang K et al. *JAMA Network Open*

116	Gastrointestinal Adverse Events of Dipeptidyl Peptidase 4 Inhibitors in Type 2 Diabetes: A Systematic Review and Network Meta-analysis
**Reason: Inappropriate outcome measure**	
117	Glucagon-like peptide-1 receptor agonists and microvascular outcomes in type 2 diabetes: A systematic review and meta-analysis
118	Effect of GLP-1 receptor agonists on microvascular endpoints in type 2 diabetes: a systematic review and meta-analysis
119	Efficacy and safety of glucagon-like peptide-1 agonists on macrovascular and microvascular events in type 2 diabetes mellitus: A meta-analysis
120	Microvascular effects of glucagon-like peptide-1 receptor agonists in type 2 diabetes: a meta-analysis of randomized controlled trials
121	Influence of glucagon-like peptide-1 receptor agonists on fat accumulation in patients with diabetes mellitus and non-alcoholic fatty liver disease or obesity: A systematic review and meta-analysis of randomized control trials
122	Efficacy and Safety of GLP-1 Receptor Agonists in Patients With Metabolic Dysfunction-Associated Steatotic Liver Disease: A Systematic Review and Meta-Analysis of Randomized Controlled Trials
123	Effects of GLP-1 receptor agonists on the degree of liver fibrosis and CRP in non-alcoholic fatty liver disease and non-alcoholic steatohepatitis: A systematic review and meta-analysis
124	Comparative efficacy of diabetes medications on liver enzymes and fat fraction in patients with nonalcoholic fatty liver disease: A network meta- analysis
125	The effects of GLP-1 receptor agonists on visceral fat and liver ectopic fat in an adult population with or without diabetes and nonalcoholic fatty liver disease: A systematic review and meta-analysis
126	Effect of novel glucose lowering agents on non-alcoholic fatty liver disease: A systematic review and meta-analysis
127	Efficacy and Safety of GLP-1 Receptor Agonists in Patients With Type 2 Diabetes Mellitus and Non-Alcoholic Fatty Liver Disease: A Systematic Review and Meta-Analysis
128	Glucagon-Like Peptide-1 Receptor Agonists for Non-Alcoholic Fatty Liver Disease in Type 2 Diabetes: A Meta-Analysis
129	The effects of glucagon-like peptide-1 receptor agonists on glycemic control and anthropometric profiles among diabetic patients with non-alcoholic fatty liver disease: A systematic review and meta-analysis of randomized controlled trials
130	Antidiabetic drugs and non-alcoholic fatty liver disease: A systematic review, meta-analysis and evidence map
131	Efficacy and Safety of Glucagon-like Peptide-1 Receptor Agonists in the Treatment of Metabolic Associated Fatty Liver Disease: A Systematic Review and Meta-analysis
132	A meta-analysis of the effects of glucagon-like-peptide 1 receptor agonist (GLP1-RA) in nonalcoholic fatty liver disease (NAFLD) with type 2 diabetes (T2D)
133	Comparison of the Efficacy of Glucagon-Like Peptide-1 Receptor Agonists in Patients With Metabolic Associated Fatty Liver Disease: Updated Systematic Review and Meta-Analysis
134	The efficacy of glucagon-like peptide 1 receptor agonists in patients with non-alcoholic fatty liver disease: a systematic review and meta-analysis of randomized controlled trials
135	Comparison of the Efficacy of Glucagon-Like Peptide-1 Receptor Agonists in Patients With Metabolic Associated Fatty Liver Disease: Updated Systematic Review and Meta-Analysis
136	A Meta-Analysis of the Efficacy of Glucagon-Like Peptide-1 Receptor Agonists on Nonalcoholic Fatty Liver Disease in Patients with Type 2 Diabetes Mellitus

© 2026 Yang K et al. *JAMA Network Open*

137	Efficacy and safety of glucagon-like peptide-1 receptor agonists in non-alcoholic fatty liver disease: A systematic review and meta-analysis
138	Pharmacokinetics, safety, and efficacy of DPP-4 inhibitors and GLP-1 receptor agonists in patients with type 2 diabetes mellitus and renal or hepatic impairment. A systematic review of the literature
139	Incretin-based therapies for the treatment of non-alcoholic fatty liver disease: A systematic review and meta-analysis
140	Efficacy and safety of glucagon-like peptide-1 agonists on macrovascular and microvascular events in type 2 diabetes mellitus: A meta-analysis.
141	Efficacy and safety of incretin therapy in type 2 diabetes: systematic review and meta-analysis.
142	Effectiveness and safety of new oral and injectable agents for in-hospital management of type 2 diabetes in general wards: Systematic review and meta-analysis.
143	Comparative efficacy and safety of long-acting insulin analogs in patients with type 2 diabetes failing on oral therapy: Systemic review and meta-analyses.
144	Glucose lowering efficacy of glucagon-like peptide-1 receptor agonists: A meta-analysis.
145	Proportion of patients at HbA1c target <7% with eight classes of antidiabetic drugs in type 2 diabetes: systematic review of 218 randomized controlled trials with 78 945 patients.
146	Efficacy and Safety of Lixisenatide in Japanese Patients with Type 2 Diabetes Insufficiently Controlled with Basal Insulin±Su1fonylurea: A Sub-analysis of the GetGoal-L-Asia Study.
147	Saxagliptin and sitagliptin in adult patients with type 2 diabetes: a systematic review and meta-analysis.
148	Insulin vs GLP-1 analogues in poorly controlled Type 2 diabetic subjects on oral therapy: a meta-analysis.
149	Meta-analysis of randomized controlled trials of lixisenatide as add on to basal insulin and/or oral antihyperglycemic agents in patients with type 2 diabetes mellitus.
**Reason: Animal study (non-human research)**	
150	Glucagon-like peptide-1 receptor agonists (GLP-1RAs) as treatment for nicotine cessation in psychiatric populations: a systematic review
151	Gut Microbiota Modification via Glucagon-like Peptide-1 with Beneficial Neuroprotective Effects
**Reason: High overlap with already included studies**	
152	Use of glucagon-like peptide-1 receptor agonists and bone fractures: a meta-analysis of randomized clinical trials.
153	Association of GLP-1 Receptor Agonists With Risk of Suicidal Ideation and Behavior: A Systematic Review and Meta-Analysis.
154	Semaglutide and cancer: A systematic review and meta-analysis.
155	Evidence Report on the Safety of Gastrointestinal Endoscopy in Patients on Glucagon-like Peptide-1 Receptor Agonists: A Systematic Review and Meta-Analysis.
156	Glucagon-like peptide-1 receptor agonists increase the risk of residual gastric content and pulmonary aspiration on upper endoscopy: A meta-analysis.
157	Effects of glucagon-like peptide-1 receptor agonists on endoscopy outcomes: systematic review and meta-analysis.
158	Effects of Glucagon-Like Peptide-1 Receptor Agonists on Upper Gastrointestinal Endoscopy: A Meta-Analysis.
159	Glucagon-like peptide-1 receptor agonists and risk of acute pancreatitis in patients with type 2 diabetes.
160	Efficacy and safety of GLP-1 agonists in Parkinson's disease: a systematic review and meta-analysis of randomized controlled trials.

© 2026 Yang K et al. *JAMA Network Open*

161	Risk of acute pancreatitis with incretin-based therapy: a systematic review and updated meta-analysis of cardiovascular outcomes trials.

© 2026 Yang K et al. *JAMA Network Open*

eTable 8. Outcomes Included in the Umbrella Review.

Major outcomes	Unique outcomes	No.
Adverse events	Serious infections	1
Adverse events	Total infections	2
Adverse events	Non-serious infections	3
Adverse events	Infections as immune-mediated events	4
Adverse events	Serious adverse events	5
Adverse events	Nausea	6
Adverse events	Vomiting	7
Adverse events	Diarrhea	8
Adverse events	Overall gastrointestinal adverse events	9
Adverse events	Adverse events	10
Adverse events	Injection-site reactions	11
Adverse events	Headache	12
Adverse events	Constipation	13
Adverse events	Pain relief	14
Adverse events	Upper respiratory tract infection	15
Adverse events	Lower respiratory tract infection	16
Adverse events	Gastrointestinal reaction	17
Adverse events	Adverse events (versus active comparator)	18
Adverse events	Serious adverse events (versus active comparator)	19
Adverse events	Serious adverse events (versus placebo)	20
Adverse events	Urinary tract infection	21
Adverse events	Adverse events (versus placebo)	22
Adverse events	Serious adverse events (versus placebo-controlled group)	23
Adverse events	Treatment-emergent adverse events (versus placebo-controlled group)	24
Adverse events	Other adverse reactions	25

© 2026 Yang K et al. *JAMA Network Open*

Adverse events	Anxiety	26
Adverse events	Back pain	27
Adverse events	Injection site reaction	28
Adverse events	Severe adverse events	29
Adverse events	Treatment-emergent adverse events	30
Adverse events	Serious adverse events (versus active-controlled group)	31
Adverse events	Treatment-emergent adverse events (versus active-controlled group)	32
Adverse events	Loss of appetite	33
Adverse events	Decreased appetite	34
Adverse events	Dyspepsia	35
Adverse events	Fatigue	36
Adverse events	Stomach pain	37
Respiratory	Asthma	38
Respiratory	Incidence of respiratory diseases	39
Respiratory	Bronchitis	40
Respiratory	Lung adenocarcinoma	41
Respiratory	Squamous cell carcinoma of lung	42
Respiratory	Acute respiratory failure	43
Respiratory	Chronic obstructive pulmonary disease	44
Respiratory	Pneumonia	45
Respiratory	Upper respiratory tract infection	46
Respiratory	Pulmonary fibrosis	47
Respiratory	Pulmonary oedema	48
Respiratory	Sleep apnoea syndrome	49
Respiratory	Interstitial lung disease	50
Fracture	Fracture risk	51
Fracture	Risk of fracture	52
Fracture	Incidence of bone fractures	53
Fracture	Fracture incidence	54
Gastrointestinal	Pancreatitis	55

© 2026 Yang K et al. *JAMA Network Open*

Gastrointestinal	Cholelithiasis	56
Gastrointestinal	Cholecystitis	57
Gastrointestinal	Gallbladder or biliary diseases	58
Gastrointestinal	Acute pancreatitis	59
Gastrointestinal	Gastroesophageal reflux disease	60
Gastrointestinal	Gastrointestinal hemorrhage	61
Gastrointestinal	Gastritis	62
Gastrointestinal	Biliary diseases	63
Gastrointestinal	Intestinal obstruction	64
Gastrointestinal	Gastrointestinal ulceration	65
Gastrointestinal	Gastrointestinal ischemia	66
Gastrointestinal	Esophagitis	67
Gastrointestinal	Acute cholecystitis	68
Gastrointestinal	Cholestasis	69
Gastrointestinal	Gastrointestinal perforation	70
Gastrointestinal	Gastric ulcer	71
Gastrointestinal	Gastroparesis	72
Gastrointestinal	Cholangitis	73
Gastrointestinal	Gastric ulcer hemorrhage	74
Gastrointestinal	Paralytic ileus	75
Gastrointestinal	Chronic pancreatitis	76
Cancer	Pancreatic cancer	77
Cancer	Thyroid cancer	78
Cancer	Gastrointestinal cancer	79
Cancer	Overall cancer	80
Cancer	Breast cancer	81
Cancer	Neoplasms	82
Cancer	Colorectal cancer	83
Cancer	Cancer	84
Cancer	Overall thyroid cancer	85
Cancer	Endometrial cancer	86
Cancer	Kidney cancer	87

© 2026 Yang K et al. *JAMA Network Open*

Cancer	Uterine cancer	88
Cancer	Gallbladder cancer	89
Cancer	Biliary cancer	90
Cancer	Gastric cancer	91
Cancer	Ovarian cancer	92
Cancer	Bladder cancer	93
Cancer	Liver cancer	94
Cancer	Esophageal cancer	95
Cancer	Multiple myeloma	96
Cancer	Meningioma	97
Endocrine	Goiter	98
Endocrine	Hyperthyroidism	99
Endocrine	Hypothyroidism	100
Endocrine	Thyroid mass	101
Endocrine	Thyroiditis	102
Psychiatric	Suicidal behavior	103
Psychiatric	Suicide and/or self-harm adverse events	104
Psychiatric	Psychiatric disorder	105
Psychiatric	Suicidal behavior	106
Psychiatric	Depression	107
Psychiatric	Anxiety	108
Hepatic	Metabolic dysfunction-associated steatohepatitis resolution	109
Neurological	All-cause dementia	110
Neurological	Vascular dementia	111
Neurological	Alzheimer dementia	112
Neurological	Seizure and epilepsy risk	113
Neurological	Seizure risk	114
Neurological	Epilepsy risk	115
Neurological	Parkinson’s disease	116

© 2026 Yang K et al. *JAMA Network Open*

eTable 9. Summary of Associations Between Glucagon-Like Peptide-1 Receptor Agonists and Selected Outcomes, Stratified by Drug Type.

Author, year	Outcomes	Intervention	No. of RCTs	Total participants	Events/participants (Intervention), %	Events/participants (Comparator), %	Effect size	P-value for effect size	I^2	95% PI
**Hepatic Outcomes**										
Alessandro et al, 2025	Fibrosis stage	Semaglutide	4	1532	269 / 975 (27.6%)	117 / 557 (21.0%)	1.48(1.15-1.91)	0.002	0	1.15-1.91
Alessandro et al, 2025	Nash resolution	Semaglutide	5	1772	429 / 1182 (36.3%)	126 / 590 (21.4%)	1.98(1.57-2.50)	0.00	0	1.57-2.50
Lise et al, 2014	Normalization of alanine aminotransferase	Lixisenatide	14	1654	361 / 988 (36.5%)	226 / 666 (33.9%)	1.06(0.93-1.22)	0.39	0	0.93-1.22
Lise et al, 2014	Normalization of aspartate aminotransferase	Lixisenatide	14	838	191 / 494 (38.7%)	128 / 344 (37.2%)	1.02(0.85-1.23)	0.84	0	0.85-1.23
**Respiratory Outcomes**										
Zhang et al, 2024	Asthma	Albiglutide	5	18793	2160 / 10149 (21.3%)	905 / 8644 (10.5%)	1.05(0.67-1.65)	0.84	93.3	0.37-2.99
Zhang et al, 2024	Asthma	Dulaglutide	3	11508	263 / 5795 (4.5%)	305 / 5713 (5.3%)	0.86(0.74-1.00)	0.05	0	0.74-1.00
Zhang et al, 2024	Asthma	Exenatide	6	17927	581 / 8932 (6.5%)	608 / 8995 (6.8%)	0.97(0.86-1.09)	0.64	10	0.83-1.13
Zhang et al, 2024	Asthma	Liraglutide	7	22696	2721 / 11997 (22.7%)	1512 / 10699 (14.1%)	1.07(0.78-1.46)	0.69	80.2	0.54-2.12
Zhang et al, 2024	Asthma	Lixisenatide	3	8460	883 / 4294 (20.6%)	752 / 4166 (18.1%)	1.43(0.93-2.20)	0.10	61.2	0.69-2.97
Zhang et al, 2024	Asthma	Semaglutide	5	12593	1146 / 6755 (17.0%)	649 / 5838 (11.1%)	1.13(0.78-1.64)	0.51	88.6	0.51-2.52
**Fracture Outcomes**										
Cheng et al, 2019	Risk of fracture	Albiglutide	4	2115	5 / 1243 (0.4%)	3 / 872 (0.3%)	1.22(0.25-5.91)	0.80	5.6	0.21-7.00
Cheng et al, 2019	Risk of fracture	Dulaglutide	5	3300	10 / 2146 (0.5%)	5 / 1154 (0.4%)	0.910.33-2.47()	0.85	0	0.33-2.47
Cheng et al, 2019	Risk of fracture	Exenatide	9	4158	17 / 2091 (0.8%)	8 / 2067 (0.4%)	1.63(0.73-3.63)	0.23	0	0.73-3.63
Cheng et al, 2019	Risk of fracture	Liraglutide	9	13676	40 / 7382 (0.5%)	64 / 6294 (1.0%)	0.58(0.39-0.86)	0.01	0	0.39-0.86
Cheng et al, 2019	Risk of fracture	Lixisenatide	3	7405	18 / 3697 (0.5%)	33 / 3708 (0.9%)	0.55(0.31-0.98)	0.04	0	0.31-0.98

© 2026 Yang K et al. *JAMA Network Open*

Mabilleau et al, 2014	Incidence of bone fractures	Exenatide	3	1046	5 / 538 (0.9%)	1 / 508 (0.2%)	2.31(0.36-15.01)	0.38	10.1	0.27-19.55
Mabilleau et al, 2014	Incidence of bone fractures	Liraglutide	4	3228	8 / 2393 (0.3%)	5 / 835 (0.6%)	0.46(0.15-1.43)	0.18	0	0.15-1.43
Su et al, 2015	Incidence of bone fractures	Exenatide	8	3937	14 / 2002 (0.7%)	7 / 1935 (0.4%)	1.49(0.64-3.48)	0.36	0	0.64-3.48
Su et al, 2015	Incidence of bone fractures	Liraglutide	8	5939	13 / 3750 (0.3%)	14 / 2189 (0.6%)	0.59(0.24-1.44)	0.25	0	0.24-1.44
Zhang et al, 2025	Fracture risk	Albiglutide	7	12439	38 / 6345 (0.6%)	40 / 6094 (0.7%)	0.92(0.59-1.45)	0.73	0	0.59-1.45
Zhang et al, 2025	Fracture risk	Dulaglutide	6	3653	11 / 2429 (0.4%)	8 / 1224 (0.6%)	0.7(0.28-1.77)	0.45	0	0.28-1.77
Zhang et al, 2025	Fracture risk	Exenatide	8	3884	15 / 1953 (0.8%)	10 / 1931 (0.5%)	1.38(0.61-3.12)	0.44	0	0.61-3.12
Zhang et al, 2025	Fracture risk	Liraglutide	12	6862	10 / 4230 (0.2%)	16 / 2632 (0.6%)	0.43(0.20-0.93)	0.03	0	0.20-0.93
Zhang et al, 2025	Fracture risk	Semaglutide	5	8158	24 / 4384 (0.6%)	26 / 3774 (0.7%)	0.81(0.47-1.42)	0.46	0	0.47-1.42
**Endocrine Outcomes**										
Hu et al, 2022	Overall thyroid disorders	Oral semaglutide	5	6381	7 / 3950 (0.2%)	3 / 2431(0.1%)	0.92(0.27-3.11)	0.89	0	0.27-3.11
Hu et al, 2022	Overall thyroid disorders	Subcutaneous semaglutide	6	9047	9 / 5628 (0.2%)	8 / 3419(0.2%)	0.66(0.26-1.68)	0.38	0	0.26-1.68
**Gastrointestinal Outcomes**										
Chiang et al, 2025	Cholelithiasis	Dulaglutide	3	10883	53 / 5646 (0.9%)	57 / 5237 (1.1%)	0.91(0.63-1.32)	0.61	0	0.63-1.32
Chiang et al, 2025	Gastritis	Dulaglutide	3	10883	20 / 5646 (0.3%)	19 / 5237 (0.4%)	0.99(0.53-1.85)	0.97	0	0.53-1.85
Chiang et al, 2025	Gastrointestinal ulceration	Dulaglutide	3	20484	15 / 10445 (0.1%)	17 / 10039 (0.2%)	0.82(0.41-1.64)	0.57	0	0.41-1.64
Alves et al, 2012	Acute pancreatitis	Exenatide	13	769769	82 / 58372 (0.1%)	1092 / 711397 (0.1%)	0.82(0.55-1.23)	0.34	24	0.40-1.68
Masson et al, 2024	Acute pancreatitis	High-dose subcutaneous semaglutide (2.4 mg once weekly)	9	23668	22 / 12726 (0.2%)	26 / 10942 (0.2%)	0.72(0.42-1.26)	0.25	0	0.42-1.26
Alves et al, 2012	Acute pancreatitis	Liraglutide	12	7672	5 / 4795 (0.1%)	2 / 2877 (0.1%)	0.81(0.29-2.26)	0.69	0	0.29-2.26

© 2026 Yang K et al. *JAMA Network Open*

Chiang et al, 2025	Cholecystitis	Liraglutide	10	15433	74 / 8579 (0.9%)	36 / 6854 (0.5%)	1.63(1.09-2.43)	0.02	0	1.09-2.43
Chiang et al, 2025	Cholelithiasis	Liraglutide	7	14923	94 / 8272 (1.1%)	40 / 6651 (0.6%)	1.79(1.23-2.60)	0.002	0	1.23-2.60
Chiang et al, 2025	Gastroesophageal reflux disease	Liraglutide	8	15215	192 / 8448 (2.3%)	45 / 6767 (0.7%)	2.55(1.31-4.97)	0.01	56.7	0.63-10.36
Chiang et al, 2025	Gastrointestinal hemorrhage	Liraglutide	6	14032	49 / 7686 (0.6%)	51 / 6346 (0.8%)	0.94(0.64-1.38)	0.74	0	0.64-1.38
Chiang et al, 2025	Gastrointestinal ischemia	Liraglutide	4	13485	7 / 7361 (0.1%)	4 / 6124 (0.1%)	1.32(0.44-3.92)	0.62	0	0.44-3.92
Chiang et al, 2025	Gastrointestinal ulceration	Liraglutide	6	23639	22 / 12644 (0.2%)	23 / 10995 (0.2%)	0.9(0.51-1.59)	0.72	0	0.51-1.59
Chiang et al, 2025	Intestinal obstruction	Liraglutide	4	13419	14 / 7325 (0.2%)	16 / 6094 (0.3%)	0.84(0.42-1.68)	0.61	0	0.42-1.68
Chiang et al, 2025	Pancreatitis	Liraglutide	5	13695	39 / 7447 (0.5%)	34 / 6248 (0.5%)	1.07(0.67-1.71)	0.78	0	0.67-1.71
Chiang et al, 2025	Cholecystitis	Lixisenatide	5	8530	23 / 4660 (0.5%)	16 / 3870 (0.4%)	0.95(0.36-2.50)	0.92	19.3	0.23-3.94
Chiang et al, 2025	Cholelithiasis	Lixisenatide	3	7602	15 / 4115 (0.4%)	8 / 3487 (0.2%)	1.15(0.26-5.11)	0.85	31	0.13-10.26
Chiang et al, 2025	Gastritis	Lixisenatide	4	8084	25 / 4437 (0.6%)	12 / 3647 (0.3%)	1.3(0.66-2.56)	0.45	0	0.66-2.56
Chiang et al, 2025	Gastrointestinal hemorrhage	Lixisenatide	3	7189	18 / 3764 (0.5%)	22 / 3425 (0.6%)	0.67(0.24-1.88)	0.45	14.4	0.16-2.73
Masson et al, 2024	Acute pancreatitis	Oral semaglutide (different doses)	8	6657	3 / 3943 (0.1%)	4 / 2714 (0.1%)	0.44(0.14-1.42)	0.17	0	0.14-1.42
Masson et al, 2024	Acute pancreatitis	Semaglutide	22	34869	35 / 19068 (0.2%)	42 / 15801 (0.3%)	0.7(0.46-1.06)	0.10	0	0.46-1.06
Chiang et al, 2025	Cholangitis	Semaglutide	4	27616	14 / 13809 (0.1%)	10 / 13807 (0.1%)	1.34(0.61-2.95)	0.47	0	0.61-2.95
Chiang et al, 2025	Cholecystitis	Semaglutide	13	34650	88 / 18454 (0.5%)	85 / 16196 (0.5%)	0.91(0.67-1.23)	0.54	0	0.67-1.23
Safwan et al, 2025	Cholelithiasis	Semaglutide	21	39662	185 / 21719 (0.8%)	90 / 17943 (0.5%)	1.52(1.17-1.97)	0.002	0	1.17-1.97
Chiang et al, 2025	Cholestasis	Semaglutide	4	25045	5 / 12625 (0.0%)	6 / 12420 (0.0%)	0.92(0.27-3.16)	0.89	0	0.27-3.16
Chiang et al, 2025	Esophagitis	Semaglutide	4	24706	6 / 12419 (0.0%)	6 / 12287 (0.0%)	0.99(0.32-3.07)	0.99	0	0.32-3.07
Chiang et al, 2025	Gastritis	Semaglutide	7	29513	30 / 15024 (0.2%)	24 / 14489 (0.2%)	1.17(0.69-1.98)	0.56	0	0.69-1.98
Chiang et al, 2025	Gastroesophageal reflux disease	Semaglutide	13	33884	222 / 18013 (1.2%)	55 / 15871 (0.3%)	2.25(1.51-3.35)	< 0.001	22	1.07-4.71

© 2026 Yang K et al. *JAMA Network Open*

Chiang et al, 2025	Gastrointestinal hemorrhage	Semaglutide	8	30135	86 / 15564 (0.6%)	89 / 14571 (0.6%)	0.93(0.69-1.25)	0.64	0	0.69-1.25
Chiang et al, 2025	Gastrointestinal ischemia	Semaglutide	8	30807	19 / 15827 (0.1%)	10 / 14980 (0.1%)	1.55(0.75-3.19)	0.23	0	0.75-3.19
Chiang et al, 2025	Gastrointestinal ulceration	Semaglutide	12	58761	22 / 30498 (0.1%)	34 / 28263 (0.1%)	0.63(0.36-1.08)	0.09	0	0.36-1.08
Chiang et al, 2025	Intestinal obstruction	Semaglutide	8	27984	34 / 14527 (0.2%)	28 / 13457 (0.2%)	1.13(0.69-1.85)	0.63	0	0.69-1.85
Safwan et al, 2025	Pancreatitis	Semaglutide	18	65924	79 / 34737 (0.2%)	94 / 31187 (0.3%)	0.76(0.56-1.02)	0.07	0	0.56-1.02
Chiang et al, 2025	Paralytic ileus	Semaglutide	3	24434	5 / 12218 (0.0%)	5 / 12216 (0.0%)	0.91(0.26-3.14)	0.88	0	0.26-3.14
He et al, 2022	Gallbladder or biliary diseases	Albiglutide	8	14093	36 / 6914 (0.5%)	30 / 7179 (0.4%)	1.1(0.57-2.13)	0.78	10.9	0.43-2.84
He et al, 2022	Gallbladder or biliary diseases	Dulaglutide	11	17630	149 / 10004 (1.5%)	98 / 7626 (1.3%)	1.33(1.03-1.72)	0.03	0	1.03-1.72
He et al, 2022	Gallbladder or biliary diseases	Exenatide	9	19381	190 / 9726 (1.9%)	154 / 9655 (1.6%)	1.22(0.99-1.51)	0.07	0	0.99-1.51
He et al, 2022	Gallbladder or biliary diseases	Liraglutide	21	21897	276 / 12687 (2.2%)	117 / 9210 (1.3%)	1.83(1.36-2.45)	< 0.001	4.5	1.20-2.79
He et al, 2022	Gallbladder or biliary diseases	Lixisenatide	6	9080	39 / 5022 (0.8%)	26 / 4058 (0.6%)	0.85(0.31-2.30)	0.75	29.4	0.16-4.47
He et al, 2022	Gallbladder or biliary diseases	Subcutaneous semaglutide	14	14185	193 / 9031 (2.1%)	92 / 5154 (1.8%)	1.37(0.94-1.99)	0.10	19	0.70-2.71
He et al, 2022	Gallbladder or biliary diseases	Oral semaglutide	8	8696	28 / 5383 (0.5%)	21 / 3313 (0.6%)	0.75(0.43-1.31)	0.31	0	0.43-1.31
**Cancer Outcomes**										
Guo et al, 2016	Cancer	Albiglutide	9	4555	32 / 2549 (1.3%)	18 / 2006 (0.9%)	1.27(0.70-2.32)	0.44	0	0.70-2.32
Guo et al, 2016	Cancer	Dulaglutide	11	7220	42 / 4580 (0.9%)	16 / 2640 (0.6%)	1.18(0.65-2.12)	0.59	0	0.65-2.12
Ko et al, 2025	Pancreatic cancer	Dulaglutide	4	21184	40 / 11004 (0.4%)	22 / 10180 (0.2%)	1.66(1.00-2.77)	0.05	0	1.00-2.77
Ko et al, 2025	Thyroid cancer	Dulaglutide	3	29676	12 / 14829 (0.1%)	0 / 14847 (0.0%)	7.89(1.41-44.22)	0.02	0	1.41-44.22
Alves et al, 2011	Overall cancer	Exenatide	10	3881	7 / 1938 (0.4%)	7 / 1943 (0.4%)	0.88(0.32-2.36)	0.79	0	0.32-2.36
Ko et al, 2025	Pancreatic cancer	Exenatide	4	30360	32 / 15150 (0.2%)	30 / 15210 (0.2%)	1.06(0.65-1.73)	0.82	0	0.65-1.73

© 2026 Yang K et al. *JAMA Network Open*

Guo et al, 2016	Cancer	Extended-release exenatide	6	2786	6 / 1315 (0.5%)	9 / 1471 (0.6%)	0.72(0.24-2.11)	0.55	0	0.24-2.11
Ko et al, 2025	Breast cancer	Liraglutide	6	14116	34 / 7659 (0.4%)	23 / 6457 (0.4%)	1.15(0.67-1.99)	0.61	0	0.67-1.99
Ko et al, 2025	Colorectal cancer	Liraglutide	6	14506	39 / 8114 (0.5%)	28 / 6392 (0.4%)	1.24(0.77-2.00)	0.39	0	0.77-2.00
Alves et al, 2012	Overall cancer	Liraglutide	10	5788	39 / 3721 (1.0%)	14 / 2067 (0.7%)	1.34(0.70-2.58)	0.37	0	0.70-2.58
Ko et al, 2025	Pancreatic cancer	Liraglutide	9	27554	28 / 15074 (0.2%)	22 / 12480 (0.2%)	1.17(0.69-1.99)	0.57	0	0.69-1.99
Ko et al, 2025	Thyroid cancer	Liraglutide	12	34762	19 / 18388 (0.1%)	15 / 16374 (0.1%)	1.03(0.55-1.95)	0.92	0	0.55-1.95
Ko et al, 2025	Breast cancer	Lixisenatide	3	7404	4 / 3927 (0.1%)	4 / 3477 (0.1%)	0.79(0.16-3.81)	0.77	15.4	0.11-5.47
Ko et al, 2025	Colorectal cancer	Lixisenatide	4	8048	15 / 4338 (0.3%)	10 / 3710 (0.3%)	1.26(0.58-2.75)	0.56	0	0.58-2.75
Ko et al, 2025	Kidney cancer	Lixisenatide	3	7225	6 / 3863 (0.2%)	3 / 3362 (0.1%)	1.18(0.32-4.32)	0.80	0	0.32-4.32
Ko et al, 2025	Pancreatic cancer	Lixisenatide	6	14450	6 / 7726 (0.1%)	10 / 6724 (0.1%)	0.47(0.16-1.42)	0.18	0	0.16-1.42
Ko et al, 2025	Thyroid cancer	Lixisenatide	3	12806	3 / 6572 (0.0%)	2 / 6234 (0.0%)	1(0.19-5.33)	1.00	0	0.19-5.33
Ko et al, 2025	Breast cancer	Semaglutide	13	34646	29 / 18506 (0.2%)	27 / 16140 (0.2%)	0.91(0.54-1.52)	0.72	0	0.54-1.52
Ko et al, 2025	Colorectal cancer	Semaglutide	6	29553	62 / 15160 (0.4%)	48 / 14393 (0.3%)	1.26(0.86-1.84)	0.25	0	0.86-1.84
Ko et al, 2025	Endometrial cancer	Semaglutide	8	9832	6 / 5146 (0.1%)	14 / 4686 (0.3%)	0.43(0.18-1.04)	0.06	0	0.18-1.04
Ko et al, 2025	Gallbladder cancer	Semaglutide	5	28016	3 / 14108 (0.0%)	5 / 13908 (0.0%)	0.61(0.16-2.31)	0.46	0	0.16-2.31
Ko et al, 2025	Gastric cancer	Semaglutide	6	29018	24 / 14760 (0.2%)	15 / 14258 (0.1%)	1.33(0.68-2.61)	0.41	0	0.68-2.61
Ko et al, 2025	Kidney cancer	Semaglutide	6	28340	35 / 14271 (0.2%)	24 / 14069 (0.2%)	1.4(0.84-2.36)	0.20	0	0.84-2.36
Ko et al, 2025	Liver cancer	Semaglutide	5	28823	19 / 14614 (0.1%)	14 / 14209 (0.1%)	1.32(0.66-2.63)	0.44	0	0.66-2.63
Ko et al, 2025	Multiple myeloma	Semaglutide	4	27616	8 / 13809 (0.1%)	8 / 13807 (0.1%)	1(0.39-2.59)	1.00	0	0.39-2.59
Ko et al, 2025	Ovarian cancer	Semaglutide	3	7236	4 / 3602 (0.1%)	1 / 3634 (0.0%)	2.11(0.35-12.73)	0.42	0	0.35-12.73
Ko et al, 2025	Pancreatic cancer	Semaglutide	8	55232	26 / 27618 (0.1%)	48 / 27614 (0.2%)	0.55(0.34-0.90)	0.02	0	0.34-0.90
Ko et al, 2025	Thyroid cancer	Semaglutide	22	81163	31 / 42142 (0.1%)	14 / 39021 (0.0%)	1.28(0.74-2.22)	0.37	0	0.74-2.22
**Adverse Event Outcomes**										
Han et al, 2025	Infections as medically important events	Albiglutide	6	12739	189 / 402 (47.0%)	6090 / 12337 (49.4%)	1.1(0.89-1.37)	0.36	49.5	0.75-1.63

© 2026 Yang K et al. *JAMA Network Open*

Han et al, 2025	Non-serious infections	Albiglutide	6	3687	1126 / 2473 (45.5%)	586 / 1214 (48.3%)	0.98(0.88-1.09)	0.68	44.2	0.80-1.20
Han et al, 2025	Serious infections	Albiglutide	6	13256	385 / 6529 (5.9%)	417 / 6727 (6.2%)	1.02(0.77-1.35)	0.89	21.5	0.66-1.58
Han et al, 2025	Infections as medically important events	Dulaglutide	14	19142	414 / 808 (51.2%)	10611 / 18334 (57.9%)	1.03(0.92-1.15)	0.58	13.9	0.86-1.24
Han et al, 2025	Non-serious infections	Dulaglutide	16	20094	3548 / 6759 (52.5%)	7881 / 13335 (59.1%)	0.91(0.83-1.00)	0.06	89.4	0.65-1.28
Han et al, 2025	Serious infections	Dulaglutide	13	19302	738 / 11001 (6.7%)	706 / 8301 (8.5%)	0.96(0.87-1.06)	0.47	0	0.87-1.06
Ye et al, 2023	Gastrointestinal reactions	Exenatide	6	628	51 / 307 (16.6%)	62 / 321 (19.3%)	0.88(0.63-1.23)	0.46	0	0.63-1.23
Han et al, 2025	Infections as medically important events	Exenatide	9	19171	18 / 37 (48.6%)	9640 / 19134 (50.4%)	1.18(0.91-1.52)	0.21	0	0.91-1.52
Hu et al, 2023	Nausea	Exenatide	3	228	16 / 122 (13.1%)	12 / 106 (11.3%)	1.14(0.56-2.32)	0.73	0	0.56-2.32
Han et al, 2025	Non-serious infections	Exenatide	14	7584	609 / 1345 (45.3%)	3011 / 6239 (48.3%)	1(0.83-1.22)	0.97	83.3	0.54-1.85
Ye et al, 2023	Other adverse reactions	Exenatide	4	420	10 / 211 (4.7%)	7 / 209 (3.4%)	1.42(0.24-8.36)	0.70	49.5	0.07-29.82
Han et al, 2025	Serious infections	Exenatide	8	18825	35 / 9265 (0.4%)	41 / 9560 (0.4%)	0.99(0.61-1.62)	0.98	0	0.61-1.62
Hu et al, 2023	Vomiting	Exenatide	3	213	8 / 114 (7.0%)	5 / 99 (5.0%)	1.38(0.46-4.11)	0.56	0	0.46-4.11
Huang et al, 2025	Total infections	FALSE	14	18121	1253 / 11823 (10.6%)	900 / 6298 (14.3%)	0.72(0.63-0.83)	0.00	60.6	0.48-1.09
Han et al, 2025	Infections as medically important events	Liraglutide	23	23155	381 / 765 (49.8%)	13068 / 22390 (58.4%)	1.12(0.98-1.29)	0.10	29.1	0.82-1.53
Dimitrios et al, 2020	Nausea	Liraglutide	6	3330	779 / 2581 (30.2%)	93 / 749 (12.4%)	3.07(2.42-3.89)	0.00	0	2.42-3.89
Han et al, 2025	Non-serious infections	Liraglutide	23	13707	3574 / 5700 (62.7%)	4886 / 8007 (61.0%)	0.89(0.79-0.99)	0.03	90	0.55-1.43
Han et al, 2025	Serious infections	Liraglutide	11	21141	773 / 11680 (6.6%)	813 / 9461 (8.6%)	0.92(0.84-1.01)	0.08	0	0.84-1.01
Dimitrios et al, 2020	Vomiting	Liraglutide	4	1150	98 / 840 (11.7%)	17 / 310 (5.5%)	2.01(0.93-4.33)	0.07	27.2	0.66-6.17

© 2026 Yang K et al. *JAMA Network Open*

Han et al, 2025	Infections as medically important events	Lixisenatide	11	11900	110 / 247 (44.5%)	6135 / 11653 (52.6%)	1.13(0.88-1.45)	0.34	51	0.63-2.03
Han et al, 2025	Non-serious infections	Lixisenatide	12	12021	1190 / 2067 (57.6%)	5312 / 9954 (53.4%)	0.87(0.76-0.99)	0.04	87.4	0.57-1.33
Han et al, 2025	Serious infections	Lixisenatide	12	13216	251 / 6904 (3.6%)	257 / 6312 (4.1%)	0.92(0.77-1.09)	0.32	0	0.77-1.09
Han et al, 2025	Infections as medically important events	Oral semaglutide	10	10804	77 / 150 (51.3%)	6844 / 10654 (64.2%)	0.9(0.77-1.04)	0.14	0	0.77-1.04
Han et al, 2025	Non-serious infections	Oral semaglutide	8	7122	797 / 1110 (71.8%)	4429 / 6012 (73.7%)	0.98(0.87-1.10)	0.74	82	0.72-1.33
Mostafa et al, 2025	Headache	Rose-010 100 micrograms	3	567	19 / 298 (6.4%)	7 / 269 (2.6%)	2.54(1.02-6.35)	0.05	0	1.02-6.35
Mostafa et al, 2025	Nausea	Rose-010 100 micrograms	3	598	57 / 336 (17.0%)	0 / 262 (0.0%)	34.96(6.71-182.07)	0.00	0	6.71-182.07
Mostafa et al, 2025	Pain relief	Rose-010 100 micrograms	5	679	104 / 372 (28.0%)	57 / 307 (18.6%)	1.72(1.18-2.50)	0.005	0	1.18-2.50
Mostafa et al, 2025	Vomiting	Rose-010 100 micrograms	3	555	13 / 292 (4.4%)	1 / 263 (0.4%)	6.92(1.48-32.25)	0.01	0	1.48-32.25
Mostafa et al, 2025	Headache	Rose-010 300 micrograms	3	557	20 / 288 (6.9%)	7 / 269 (2.6%)	2.46(0.73-8.26)	0.14	32.6	0.45-13.63
Mostafa et al, 2025	Nausea	Rose-010 300 micrograms	3	624	94 / 362 (26.0%)	0 / 262 (0.0%)	52.76(10.20-272.92)	0.00	0	10.20-272.92
Mostafa et al, 2025	Pain relief	Rose-010 300 micrograms	5	681	118 / 374 (31.6%)	57 / 307 (18.6%)	2.04(1.41-2.96)	< 0.001	0	1.41-2.96

© 2026 Yang K et al. *JAMA Network Open*

Mostafa et al, 2025	Vomiting	Rose-010 300 micrograms	3	552	21 / 289 (7.3%)	1 / 263 (0.4%)	9.77(2.14-44.57)	0.003	0	2.14-44.57
Li et al, 2021	Adverse events (versus active comparator)	Semaglutide	6	6633	1736 / 3972 (43.7%)	1139 / 2661 (42.8%)	1.01(0.95-1.07)	0.73	0	0.95-1.07
Li et al, 2021	Adverse events (versus placebo)	Semaglutide	5	3359	907 / 2164 (41.9%)	481 / 1195 (40.2%)	1.04(0.96-1.13)	0.35	0	0.96-1.13
Dutta et al, 2022	Constipation	Semaglutide	3	533	50 / 266 (18.8%)	18 / 267 (6.7%)	3.15(1.43-6.97)	0.005	40.7	0.97-10.28
Dutta et al, 2022	Decreased appetite	Semaglutide	3	523	46 / 262 (17.6%)	12 / 261 (4.6%)	4.19(2.15-8.17)	0.00	0	2.15-8.17
Dutta et al, 2022	Diarrhea	Semaglutide	4	2461	213 / 1255 (17.0%)	133 / 1206 (11.0%)	1.76(1.16-2.66)	0.007	44	0.89-3.49
Han et al, 2025	Infections as medically important events	Semaglutide	22	40334	718 / 1454 (49.4%)	21087 / 38880 (54.2%)	1.08(0.95-1.21)	0.23	70.5	0.74-1.56
Avgerinos et al, 2019	Nausea	Semaglutide	15	12828	828 / 7741 (10.7%)	208 / 5087 (4.1%)	3.05(1.99-4.68)	0.00	81.8	0.69-13.52
Han et al, 2025	Non-serious infections	Semaglutide	27	41058	5301 / 9216 (57.5%)	17249 / 31842 (54.2%)	1.01(0.82-1.26)	0.89	98.9	0.34-3.01
Huang (2025) et al, 2025	Overall gastrointestinal adverse events	Semaglutide	4	2633	710 / 1900 (37.4%)	185 / 733 (25.2%)	1.81(1.48-2.20)	0.00	0	1.48-2.20
Dutta et al, 2021	Severe adverse events	Semaglutide	4	2784	318 / 1360 (23.4%)	351 / 1424 (24.6%)	1.07(0.72-1.58)	0.75	27.8	0.58-1.95
Li et al, 2021	Serious adverse events (versus active comparator)	Semaglutide	6	4087	187 / 2423 (7.7%)	142 / 1664 (8.5%)	0.93(0.73-1.18)	0.54	11.9	0.67-1.28
Li et al, 2021	Serious adverse events (versus placebo)	Semaglutide	6	5964	391 / 3239 (12.1%)	419 / 2725 (15.4%)	0.87(0.77-0.99)	0.03	0	0.77-0.99
Sillassen (2025) et al,	Serious adverse events	Semaglutide	49	54881	8017 / 30226 (26.5%)	8182 / 24655 (33.2%)	0.93(0.90-0.95)	0.00	1.4	0.90-0.96
Han et al, 2025	Serious infections	Semaglutide	32	52149	1568 / 29182 (5.4%)	1727 / 22967 (7.5%)	0.86(0.80-0.92)	0.00	0	0.80-0.92

© 2026 Yang K et al. *JAMA Network Open*

Dutta et al, 2022	Treatment-emergent adverse events	Semaglutide	4	4011	945 / 1987 (47.6%)	951 / 2024 (47.0%)	1.02(0.90-1.16)	0.73	0	0.90-1.16
Dutta et al, 2022	Vomiting	Semaglutide	4	2292	126 / 1168 (10.8%)	51 / 1124 (4.5%)	3.26(1.62-6.56)	< 0.001	43.2	1.04-10.21
Avgerinos et al, 2020	Diarrhea	Semaglutide	11	10315	494 / 6365 (7.8%)	144 / 3950 (3.6%)	1.84(1.45-2.34)	0.00	25.5	1.16-2.92
Avgerinos et al, 2020	Vomiting	Semaglutide	11	10081	334 / 6205 (5.4%)	70 / 3876 (1.8%)	2.65(1.83-3.82)	0.00	40	1.15-6.07

© 2026 Yang K et al. *JAMA Network Open*

eTable 10. Summary of Associations Between Tirzepatide and Skeletal, Respiratory, Gastrointestinal, and Cancer Outcomes.

Author, year	Population	Intervention vs Control	No. of RCTs	Outcome	Measure	Effect Size (95% CI)	I^2	P Value
Zhang et al, 2025	T2D (Age ≥18)	Tirzepatide vs Placebo/Active	3	Fracture risk	OR	1.12 (0.43-2.91)	0	0.82
Zhang et al, 2024	T2D and obesity	Tirzepatide vs Placebo/Active	3	Asthma	OR	1.81 (0.38-8.62)	98.65	0.46
Dutta et al, 2021	T2D	Tirzepatide vs Active Control	3	Serious adverse events	OR	1.25 (0.34-4.63)	0	0.74
	T2D	Tirzepatide vs Active Control	4	Serious adverse events	OR	1 (0.64-1.57)	49.88	1
	T2D	Tirzepatide vs Passive Control	3	Treatment-emergent adverse events	OR	2.26 (0.88-5.81)	73.01	0.09
	T2D	Tirzepatide vs Passive Control	4	Treatment-emergent adverse events	OR	1.43 (1.12-1.82)	48.7	**0.003**
Safwan et al, 2025	Obesity	Tirzepatide vs Placebo	2	Cholelithiasis	OR	1.19 (0.54-2.6)	0	0.66
	Obesity	Tirzepatide vs Placebo	2	Pancreatitis	OR	1.02 (0.18-5.88)	0	0.98
Chiang et al, 2025	T2D/Obesity/MASH	Tirzepatide vs Placebo	2	Cholangitis	OR	0.68 (0.06-7.58)	0	0.76
	T2D/Obesity/MASH	Tirzepatide vs Placebo	6	Cholecystitis	OR	0.91 (0.3-2.79)	0	0.87
	T2D/Obesity/MASH	Tirzepatide vs Placebo	5	Cholelithiasis	OR	1.14 (0.44-2.93)	0	0.78
	T2D/Obesity/MASH	Tirzepatide vs Placebo	1	Cholestasis	OR	0.68 (0.02-20.27)	NA	0.82
	T2D/Obesity/MASH	Tirzepatide vs Placebo	2	Esophagitis	OR	1.28 (0.13-12.14)	0	0.83
	T2D/Obesity/MASH	Tirzepatide vs Placebo	2	Gastritis	OR	0.19 (0.03-1.35)	0	0.1
	T2D/Obesity/MASH	Tirzepatide vs Placebo	4	Gastroesophageal reflux disease	OR	3.12 (1.45-6.69)	0	**0.003**
	T2D/Obesity/MASH	Tirzepatide vs Placebo	4	Gastrointestinal hemorrhage	OR	0.39 (0.1-1.5)	0	0.17
	T2D/Obesity/MASH	Tirzepatide vs Placebo	1	Gastrointestinal ischemia	OR	0.64 (0.02-19.15)	NA	0.8

© 2026 Yang K et al. *JAMA Network Open*

	T2D/Obesity/MASH	Tirzepatide vs Placebo	4	Gastrointestinal ulceration	OR	0.49 (0.09-2.68)	0	0.41
	T2D/Obesity/MASH	Tirzepatide vs Placebo	1	Gastroparesis	OR	0.51 (0.02-15.15)	NA	0.69
	T2D/Obesity/MASH	Tirzepatide vs Placebo	4	Intestinal obstruction	OR	0.87 (0.21-3.73)	0	0.86
	T2D/Obesity/MASH	Tirzepatide vs Placebo	4	Pancreatitis	OR	0.84 (0.22-3.2)	0	0.8
	T2D/Obesity/MASH	Tirzepatide vs Placebo	1	Paralytic ileus	OR	0.68 (0.02-20.27)	NA	0.82
Ko et al, 2025	T2D/Overweight	Tirzepatide vs Control	3	Breast Cancer	OR	0.44 (0.06-3.14)	0	0.41
	T2D/Overweight	Tirzepatide vs Control	2	Colorectal Cancer	OR	0.35 (0.06-1.93)	0	0.23
	T2D/Overweight	Tirzepatide vs Control	4	Endometrial Cancer	OR	1.04 (0.2-5.48)	0	0.96
	T2D/Overweight	Tirzepatide vs Control	1	Esophageal Cancer	OR	0.24 (0.01-7.31)	NA	0.41
	T2D/Overweight	Tirzepatide vs Control	2	Gallbladder Cancer	OR	0.33 (0.03-3.6)	0	0.36
	T2D/Overweight	Tirzepatide vs Control	1	Gastric Cancer	OR	1.02 (0.03-30.37)	NA	0.99
	T2D/Overweight	Tirzepatide vs Control	3	Kidney Cancer	OR	0.87 (0.15-5.07)	0	0.87
	T2D/Overweight	Tirzepatide vs Control	1	Meningioma	OR	0.68 (0.06-7.49)	NA	0.75
	T2D/Overweight	Tirzepatide vs Control	1	Multiple Myeloma	OR	0.17 (0.01-5.04)	NA	0.3
	T2D/Overweight	Tirzepatide vs Control	1	Ovarian Cancer	OR	0.17 (0.01-5.07)	NA	0.31
	T2D/Overweight	Tirzepatide vs Control	2	Pancreatic Cancer	OR	0.34 (0.05-2.41)	0	0.28
	T2D/Overweight	Tirzepatide vs Control	6	Thyroid Cancer	OR	0.7 (0.18-2.81)	0	0.62

Abbreviations: RCT(s), randomized controlled trial(s); T2D, type 2 diabetes; MASH, metabolic dysfunction–associated steatohepatitis; OR, odds ratio; CI, confidence interval; I^2, I-squared statistic (%); NA, not available.

eTable 11. Summary of Associations Between Glucagon-Like Peptide-1 Receptor Agonists and Selected Outcomes, Stratified by Treatment Duration and Dose.

Author, year	Population	Intervention vs Control	Stratification (Duration or Dose)	No. of RCTs	Outcome	Measure	Effect Size (95% CI)	I^2	P Value
**I. Treatment Duration**									
Zhang et al, 2024	T2D and obese	GLP-1RA vs Placebo/Active	<52 weeks	NA	Asthma	RR	0.67 (0.28-1.61)	0.77	>0.99
	T2D and obese	GLP-1RA vs Placebo/Active	≥52 weeks	NA	Asthma	RR	0.95 (0.69-1.32)	0.37	>0.99
Zhang et al, 2025	T2D (Age ≥18)	GLP-1RA vs Placebo/Active	≤26 weeks	11	Fracture risk	RR	0.88 (0.44-1.80)	0.00	0.63
	T2D (Age ≥18)	GLP-1RA vs Placebo/Active	26–52 weeks	17	Fracture risk	RR	0.86 (0.54-1.37)	0.00	0.85
	T2D (Age ≥18)	GLP-1RA vs Placebo/Active	53–78 weeks	5	Fracture risk	RR	0.68 (0.22-2.08)	0.00	0.84
	T2D (Age ≥18)	GLP-1RA vs Placebo/Active	>78 weeks	10	Fracture risk	RR	0.72 (0.54-0.97)	0.00	0.74
Mabilleau et al, 2014	T2D	GLP-1RA vs Placebo/Active	<52 weeks	NA	Bone fractures	OR	2.35 (0.40-13.83)	NR	0.35
	T2D	GLP-1RA vs Placebo/Active	≥52 weeks	NA	Bone fractures	OR	0.46 (0.15-1.42)	NR	0.18
Cheng et al, 2019	T2D	GLP-1RA vs Placebo/Active	≤26 weeks	13	Risk of fracture	OR	0.97 (0.54-1.77)	0.00	0.93
	T2D	GLP-1RA vs Placebo/Active	26–52 weeks	15	Risk of fracture	OR	1.19 (0.62-2.28)	0.00	0.60
	T2D	GLP-1RA vs Placebo/Active	>52 weeks	10	Risk of fracture	OR	0.59 (0.44-0.80)	0.00	<0.001
**II. Dose**									
Masson et al, 2024	NA	Semaglutide vs Placebo	SC 0.5/1.0 mg once-weekly	5	Acute pancreatitis	OR	0.80 (0.40-1.90)	0.00	NR
	NA	Semaglutide vs Placebo	High dose SC 2.4 mg once-weekly	9	Acute pancreatitis	OR	0.80 (0.40-1.40)	0.00	NR
Mostafa et al, 2025	IBS (Age 18-70)	GLP-1RA (ROSE-010) vs Placebo/standard of care	ROSE-010 100 µg	5	Pain relief	OR	2.30 (1.53-3.46)	5.70	NR
	IBS (Age 18-70)	GLP-1RA (ROSE-010) vs Placebo/standard of care	ROSE-010 300 µg	5	Pain relief	OR	3.44 (2.10-5.65)	5.90	NR
	IBS (Age 18-70)	GLP-1RA (ROSE-010) vs Placebo/standard of care	ROSE-010 100 µg	3	Vomiting	OR	8.87 (1.87-42.20)	0.00	NR
	IBS (Age 18-70)	GLP-1RA (ROSE-010) vs Placebo/standard of care	ROSE-010 300 µg	3	Vomiting	OR	12.47 (2.69-57.79)	0.00	NR
	IBS (Age 18-70)	GLP-1RA (ROSE-010) vs Placebo/standard of care	ROSE-010 100 µg	3	Headache	OR	2.72 (1.08-6.83)	0.00	NR

© 2026 Yang K et al. *JAMA Network Open*

	IBS (Age 18-70)	GLP-1RA (ROSE-010) vs Placebo/standard of care	ROSE-010 300 µg	3	Headache	OR	2.75 (0.76-9.88)	37.80	NR

Abbreviations: RCT(s), randomized controlled trial(s); GLP-1RA(s), glucagon-like peptide-1 receptor agonist(s); T2DM, type 2 diabetes; IBS, irritable bowel syndrome; RR, risk ratio; OR, odds ratio; CI, confidence interval; I^2, I-squared statistic (%); SC, subcutaneous; NA, not available; NR, not reported.

© 2026 Yang K et al. *JAMA Network Open*

eFigure 1. Overlap Among Systematic Reviews of Adverse Events, Quantified by Corrected Covered Area.

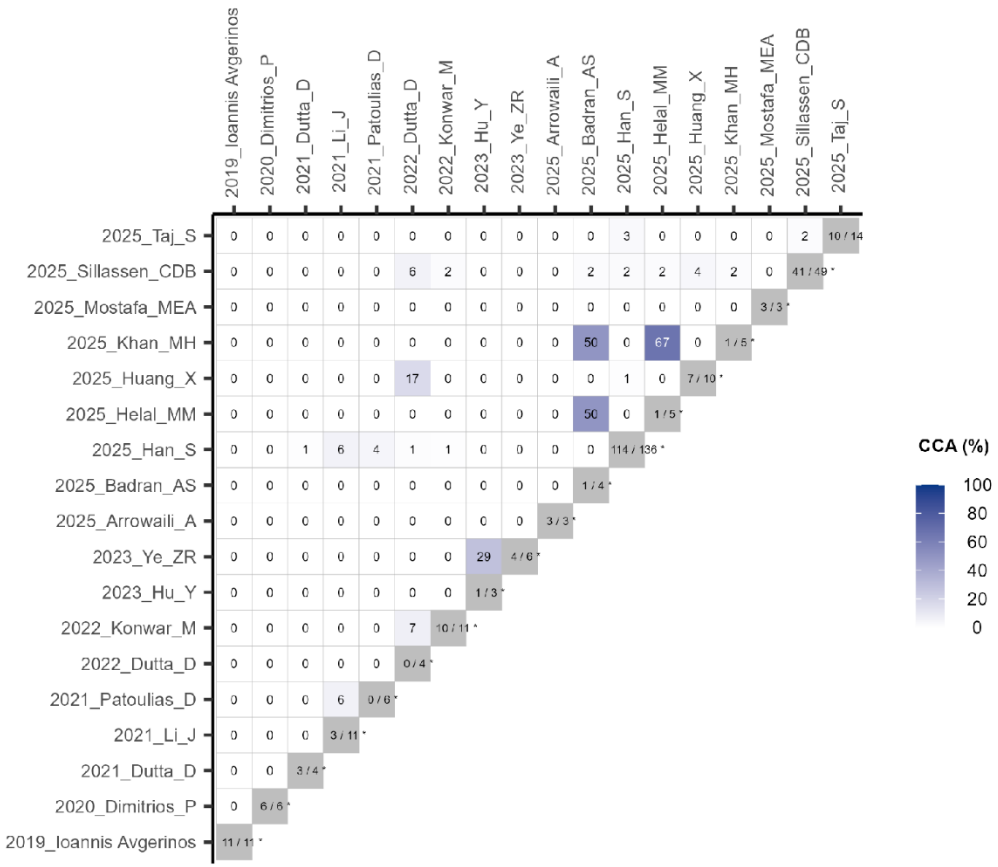

Diagonal cells provide the number of primary studies included in each review (n/N).

© 2026 Yang K et al. *JAMA Network Open*

eFigure 2. Overlap Among Systematic Reviews of Gastrointestinal (A) and Cancer (B) Outcomes, Quantified by Corrected Covered Area

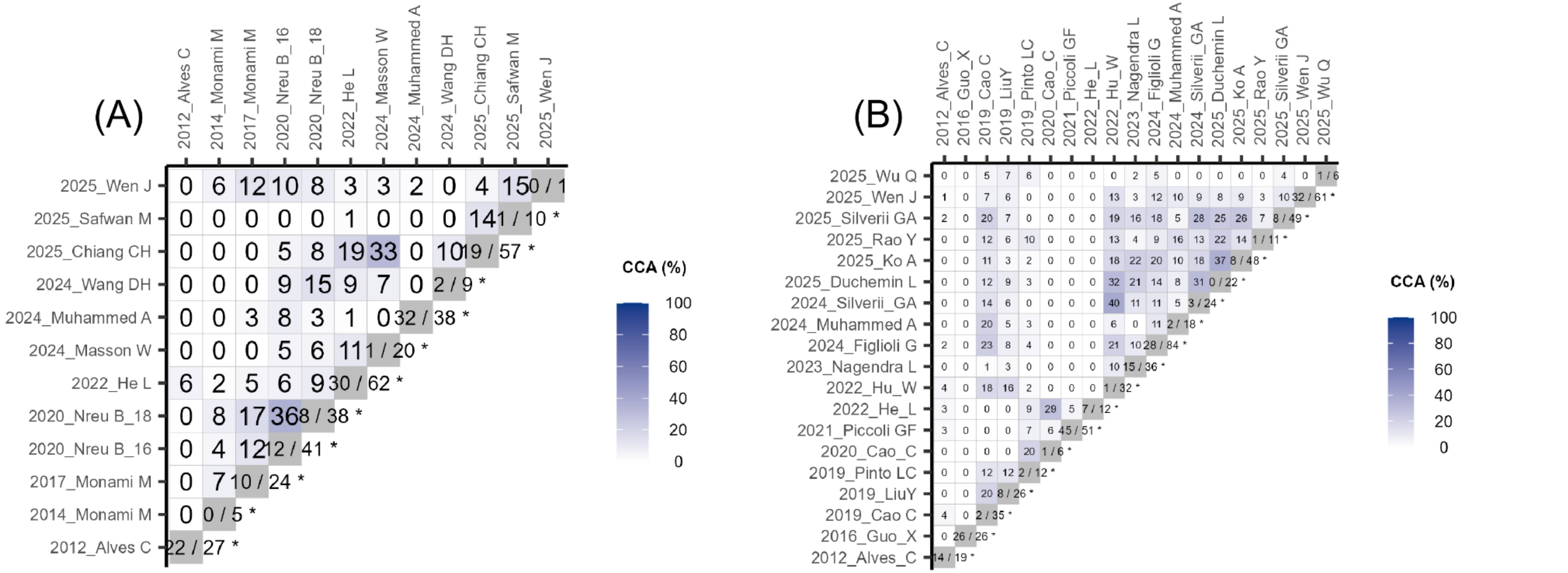

Diagonal cells provide the number of primary studies included in each review (n/N).

© 2026 Yang K et al. *JAMA Network Open*

eFigure 3. Overlap Among Systematic Reviews of Neurological(A), Respiratory(B), Fracture(C), and Psychiatric(D) Outcomes, Quantified by Corrected Covered Area.

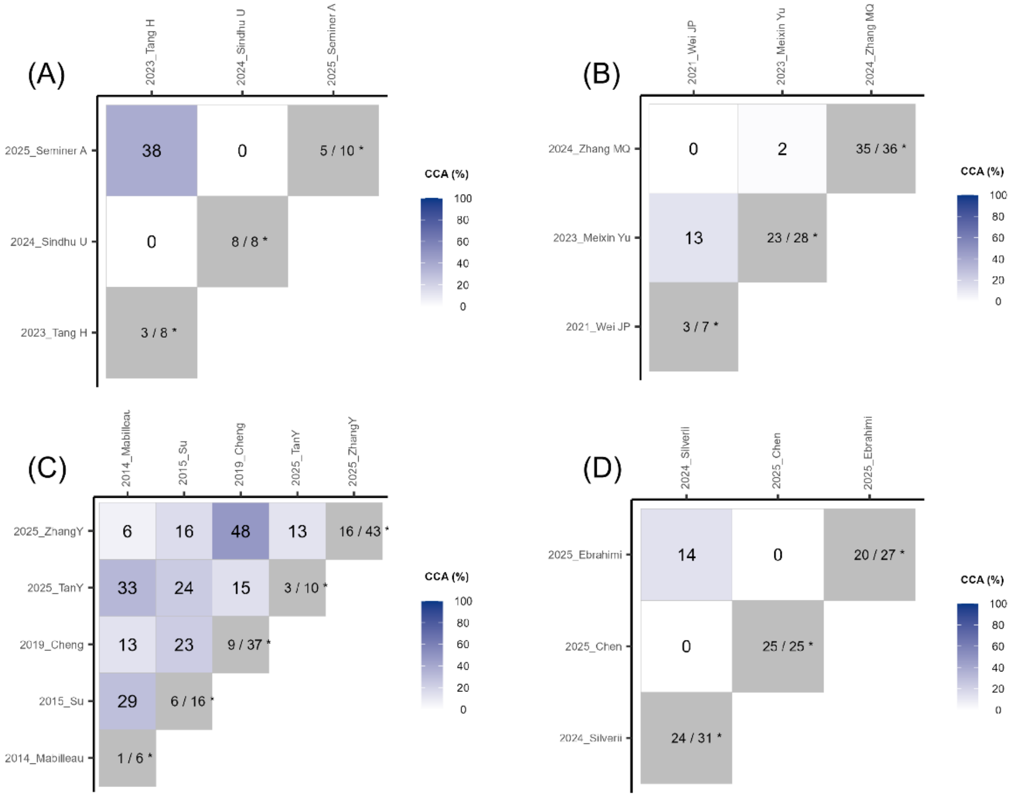

Diagonal cells provide the number of primary studies included in each review (n/N).

© 2026 Yang K et al. *JAMA Network Open*

eReferences

1. Higgins JP, Thompson SG, Deeks JJ, et al. Measuring inconsistency in meta-analyses. *BMJ (Clinical research ed)* 2003;327(7414):557-60. doi: 10.1136/bmj.327.7414.557 [published Online First: 2003/09/06]
2. Higgins JP, Thompson SG, Spiegelhalter DJ. A re-evaluation of random-effects meta-analysis. *Journal of the Royal Statistical Society Series A, (Statistics in Society)* 2009;172(1):137-59. doi: 10.1111/j.1467-985X.2008.00552.x [published Online First: 2009/04/22]
3. Ioannidis JP, Trikalinos TA. An exploratory test for an excess of significant findings. *Clinical trials (London, England)* 2007;4(3):245-53. doi: 10.1177/1740774507079441 [published Online First: 2007/08/24]

© 2026 Yang K et al. *JAMA Network Open*
