# Supplementary material for: GLP-1 Receptor Agonists and Noncardiometabolic Outcomes: An Umbrella Review of Meta-Analyses
Source: JAMA Netw Open. 2026 Mar 31;9(3):e264722. doi: 10.1001/jamanetworkopen.2026.4722 (PMC13040404; doi:10.1001/jamanetworkopen.2026.4722)
Supplement: Supplement 2. — Data Sharing Statement [file jamanetwopen-e264722-s002.pdf]

Data Sharing Statement

Yang. GLP-1 Receptor Agonists and Noncardiometabolic Outcomes. *JAMA Netw Open*. Published April 02, 2026. doi:10.1001/jamanetworkopen.2026.4722

Data
Data available: Yes
Data types: Other (please specify)
Additional Information: The datasets supporting the conclusions of this article are included within the article and its additional files.
How to access data: The datasets supporting the conclusions of this article are included within the article and its additional files.
When available: With publication

Supporting Documents
Document types: None

Additional Information
Who can access the data: Researchers whose proposed use of the data has been approved
Types of analyses: For any purpose
Mechanisms of data availability: After approval of a proposal
